# Smart transition pathways and development incentive mechanism of China’s smart community elderly care industry under market dominance: Considering a multi-subjective behavior game

**DOI:** 10.1371/journal.pone.0297696

**Published:** 2024-05-31

**Authors:** Qinghua Mao, Yining Mao, Qilong Sun, Linyao Xu

**Affiliations:** School of Economics and Management, Yanshan University, Qinhuangdao, China; Zhejiang Gongshang University, CHINA

## Abstract

Against the backdrop of an aging population, China is actively experimenting with an innovative elderly care model, so smart community elderly care has recently received widespread attention. However, the results of the implementation of the model have not yet met the expectation due to the variety of interests among the relevant participants. In this study, we identified the most core stakeholders in smart community elderly care, developed a four-party evolutionary game model including local governments, communities, service supply enterprises and households with elderly members. By applying the system dynamics method, we simulate the evolutionary paths and explore the complex interactions at the multiparticipant level in order to facilitate the transition of community elderly care services from traditional to smart, and then propose managerial insights for accelerating the construction of smart community elderly care. The results suggest that: (1) the four players in the game influence each other and are intimately related, and the benign interaction between them will further stimulate the vitality of the smart community elderly care industry; (2) appropriate improvement in policy support will strongly promote smart community elderly care, and the incentive effect on the demand side (households with elderly members) is more significant; (3) when households’ preference for smart services increases, and the perceived value to communities and enterprises reaches a certain threshold, communities and enterprises will actively adopt smart solution and technology as well as develop stable portfolio strategy; (4) measures such as simultaneously increasing the level of smart and resource synergy will promote the system evolution toward smart services, and the system is more sensitive to the internal behavior of the enterprise than the external behavior between community and enterprise.

## 1 Introduction

According to the World Health Organization’s (WHO) definition of population, people over the age of 60 are categorized as elderly. In recent decades, the elderly population has grown in almost every country, population aging has become a global trend [1]. The ever-expanding demand for nursing services has increased the requirements for quality and efficiency geriatric services worldwide. China is a rapidly aging and developing country; by 2025, the total number of people aged 60 year and above is expected to exceed 300 million, indicating a moderately aging society [2]. The progression of population aging has led to annually increasing pressure on society to provide elderly care, and the structural disequilibrium of the elderly care industry has been highlighted. Effectively meeting the needs of society based on the synergy of multiple resources has become the focus of academic attention [3].

Community-based care can meet the care needs of older groups at home and is an important model to help the elderly adapt to social development trends and improve the quality of life in old age [4]. However, with the further aggravation of aging and the progress of society, the shortcomings of traditional community elderly care services have become more and more obvious in different countries and regions. For example, service and management are generally inefficient, the contradiction between diversified demand and single supply is prominent, financing for community elderly service projects is hard, with social capital being reluctant to enter the market [5, 6]. Therefore, to adequately respond to the challenges produced by the aging population, the Chinese government has issued corresponding policies to promote the extension of professional institutional services to the community, integrate and use stock resources to develop community-based care for the elderly, and further create a new model of smart and healthy elderly care [7, 8]. Then the application of smart community elderly care in China has begun to emerge.

The smart community elderly originated as an organic integration of traditional community care and smart senior care. The concept of smart elderly care was first proposed by Unit Trust in the UK, referring to the provision of elderly care services through the use of information technology and intelligent control technology, which initially centered around smart home [1]. As early as the beginning of the 21st century, European countries established active and assisted living (AAL) research and development programs to promote the smart senior care industry [9]. In the following years, countries have begun to try to integrate smart elderly care into traditional community care services, for example, the United States has been exploring the application of information technology to elderly care services to form a smart service provision network [10]; Japan has deepened its smart medical services into the community and created the "Tokyo model" of smart community elderly care [11]. With the rapid development of science and technology, on the basis of the original smart home and health care, experts organically embedded the new science and technology of Internet of Things, big data, cloud computing and wearable devices into the community elderly service system, so that the new technology is integrated into the door-to-door service and community care service [3, 12]. A more modern and mature smart community elderly care model has developed at a fast pace. The model encourages technological, process, and content innovation in the supply elements as well as the supply mode of senior care services through information technology [3], and increases service responsiveness, coordination and feedback by integrating and synergizing multiple subjects, which realizes a highly efficient packet-to-point elderly care model as well as a more complete supply network and functional aggregation [13]. At the same time, smart community elderly care services have largely overcome the problems of unbalanced supply and demand, fragmentation of resource distribution, and decentralization of management subjects [14], thus promoting the elderly to enjoy their senior care, and enhancing the sense of well-being and security of the elderly and their children [15]. Therefore, smart community elderly care is the mainstream trend of the future development of the community care industry, as well as a national strategic choice in the context of the established modernization, digitization and intelligence [16].

Driven by a series of government policies, the smart elderly care service has witnessed significant growth in major Chinese cities, and the market size has grown from 0.17 trillion yuan in 2014 to over 4 trillion yuan by 2020 [17]. After 2020, the network-based intangible market size will gradually approach the size of the traditional tangible market, and the smart elderly industry will progressively enter the maturity period and form a perfect structural system [16]. Based on this, China’s smart community elderly care has shown a new development trend and broad development prospects. Currently, China’s smart community care has presented the coexistence of government-led public, market-led private, and social-led mutual assistance models [14]. However, the government-led public model has to a certain extent crowded out the space for other participating subjects, leading to the solidification of the center-edge power structure among participants, which affects their enthusiasm and innovation [18]. The single service model has also overburdened and pressured government services. In turn, the social-led mutual assistance model suffers from deficiencies in system building, professionalism, and unclear rights and responsibilities of multiple subjects [19]. Therefore, numerous governments have encouraged private enterprises to join the creation, operation, and maintenance of smart community elderly care, adopting a market-oriented operation mode of government subsidy, independent operation, and self-financing, forming a new elderly care system involving "government subsidy, community assistance, enterprise supply, and elderly consumption" [14], such us the Community Health Care Home in Futian District, Shenzhen; the Senior Citizen Centre in Beishan Street, Xihu District, Hangzhou, etc. Although this market-led model has been carried out in some areas of the pilot project, but its implementation has not reached the ideal expectations, how to establish new type of mechanisms to promote the construction of smart community elderly care, and then more organizations and users to join, to accelerate the realization of the transition from the traditional community elderly care model to the smart, is the urgent need in front of the decision-making departments and service provider.

Currently, research on smart community elderly care focuses on service and industry at the macro level, smart technology and application at the meso-level, and user analysis at the micro level [20]. In terms of the service model, scholars focus on such models as "Internet + community elderly services", "Artificial Intelligence + community elderly services", "healthcare-integrated smart community elderly services" and other modes [21–23], or form matching services around living needs, medical needs and psychological needs, such as life care model and personalized health management model [24, 25]. At the same time, studies have shown that the number of caregivers working in nursing homes and engaged in daily care services is declining in many parts of the world, especially in developed countries such as Europe and the United States [26], due in part to the fact that smarter elderly care services are being applied and the traditional model of elderly care is gradually scaled down. On the smart technology and application, scholars focus on new technologies such as artificial intelligence, Internet of Things and mobile health technology, as well as applications of smart devices like robots and wearables, in order to explore the impact of these technologies in the field of elderly care. Melkas Helina et al. collected data from the implementation of the care robot in a Finnish municipal elderly care service, and showed that the elderly can be successfully exercised with the help of the robot and promising signs were observed in terms of motor skills, communication skills and cognitive skills [27]. Muangprathu et al. used machine learning and integration of multiple techniques to obtain a novel tracking system for the elderly, with more than 50% of users having the highest level of satisfaction from the system satisfaction questionnaire [28]. Qian et al. consider the persistence of infectious diseases and their impact on the elderly in recent years, pointing out that AI-based applications may pose a huge demand [29]. Although the use of novel technologies in geriatric care is a young field, most of the current studies show encouraging results. Finally, as end-users, the behavior and feedback of the elderly on the technology and services should not be neglected, with special attention to the elderly cognition and sense, the degree of difficulty in operation, privacy and security [8], and how to make the elderly users break through the resistance to the use of emerging technologies and enhance the willingness to participate in smart community elderly care is the focus of scholars’ attention. Jaana et al. conducted a cross-sectional survey on mobile health technology and digital self-tracking and pointed out the substantial differences between the elderly and general adults in the use of smart technology, but at the same time, older adults and the general adult population are virtually identical in terms of satisfaction and willingness to continue using these technologies [30]. Zhang investigated the intention of older adults to use smart technologies and the influencing factors based on the digital divide theory, and the results show that facilitation enhances the interaction between perceived value and digital capability, and thus positively affects the willingness to use smart services [31]. It is widely recognized by scholars that inducing a transition in elderly people’s perceptions of traditional care is necessary so as to meet the expectations of elderly care in the face of rapid technological advances and demographic changes.

On the other hand, research on smart transition and development has been increasing in recent years, and many cities are improving the quality and performance of urban services through digitization and intelligence [32], with a focus on improving people’s lives, environmental efficiency, safety and sustainability [33]. Current studies mainly focus on administration, manufacturing, agriculture, transportation and other aspects [34]. For example, in the administrative sphere, governments are adapting to fundamental changes in the social environment through active transformation [35]. By providing intelligent, integrated, personalized and interactive public services, it is not only modernizing the public sector, but also seeking to rethink the way government operates to a large extent [36]. Hujran, O using data obtained from smart government clients in the United Arab Emirates to extend the unified theory of acceptance and use of technology to develop and validate integrated models for smart government [37]. Guo et al. concluded that promoting the deep integration of new technologies and government transformation by the paths of institutional safeguard, cultural aid, technological innovation, and ethical security is an inevitable choice for realizing a service-oriented government [38]. In the field of logistics, smart logistics cross fertilizes a number of cutting-edge technologies, such as the Internet of Things, big data analytics and artificial intelligence, with logistics technology, combining them in a cyber-physical system to enable real time monitoring and decision making, responsive communication and smoother material flow [39]. Liu et al. explored the incentives for logistics service supply chain to undergo smart transformation from a logistics service supply chain perspective [40]; Sun et al. proposed a conceptual framework for smart logistics transformation from a reverse logistics perspective, linking Industry 4.0 enablers, smart services and operations transformation, and targeted sustainable development goals [39]. In agriculture and manufacturing, the digital revolution has led to the transformation and upgrading of industries with information, knowledge and equipment as the core elements, which makes production smarter and becomes an important strategic support for promoting high-quality development of the economy [41, 42]. Yin et al. based on the ecological niche theory, established a new niche domain model for the selection of digital green innovation partners for enterprises in the advanced equipment manufacturing system in agriculture, and assisted them to carry out the practice of smart transformation in agriculture [43]. Yin et al. theoretically analyzed the pressure-state-response model of digital technology to promote smart transformation and green innovation in the manufacturing industry and pointed out that its driving mechanism mainly involves the degree of the application of digital technology, the structure of energy consumption, and the level of regional economy [44]. It can be seen that research on smart construction and transformation has been carried out in many places and many fields with some achievements. Its experience has significant reference to the field of elderly care, and to a certain extent, it also promotes the attempt to build the smart elderly care, then accelerate the development of modern and smart cities.

Combing through the above literature, it can be found that scholars have adopted a variety of models and methods for smart community elderly care as well as smart transition, and have also provided supportive insights for the construction of smart community elderly care, but the model is currently still small in scope, new in form and weak in motivation. In the smart community elderly care development, its effectiveness depends on the comprehensive role of multiple subjects and factors, the existing research in the perspective and subject is relatively single, even if the consideration of multi-body is also less to explore the complex interaction mechanism at the level of many participants, and it is difficult to reflect the decision making evolution in the process of smart community elderly construction. In addition, few scholars have approached the study of smart transition from the perspective of senior care. Therefore, in order to further realize the smart transition of community elderly care, it is necessary to construct a multi-principal synergistic system to study the promotion and development of smart community elderly care services.

Moreover, in the existing literature, we found that the top-level promotion of the creation of elderly care services has received considerable attention [45–47]. Government implementation of the concept and policy framework of active aging is urgently required to scientifically and prudently formulate supportive policies to promote the development of elderly care [48]. However, the effectiveness of government promotion is affected by the uncertainty and divergent attitudes of the other actors in the industry chain, which may hinder the degree of engagement in smart community elderly care. As such, the first study questions was: Do government measures dynamically affect the degree of smart of the remaining participants and thus the shift from traditional to smart elderly care, and if so, how? In addition to government support, the active role of the family of the elderly in the provision of elderly care has been examined. Some scholars have studied the need, satisfaction, as well as the ability and degree of use with digital technologies of elderly people with smart community care [49–51]. Nevertheless, few scholars have explored the role of older adults’ preferences in elderly services in this context. Therefore, we introduced preferences into the smart community elderly care model to explore how they impact the subject’s strategy choice under their long-term influence. At the same time, government currently does not have mandatory requirements for community and service supply enterprise to become smart. Although pilot projects have been conducted in various provinces and to varying degrees, due to the limitation of smart community senior-care services, many people are concerned about resource or the reluctance of older people to accept the new model, and the investment in smart community senior care construction may not be recovered [52], which has led to communities and service enterprises waiting to see the development between traditional and smart senior care. This is a pressing issue that deserves to be addressed. Therefore, in this study, we constructed a four-party evolutionary game model around the market-driven model of government subsidy—community assistance—enterprise supply—elderly consumption. We aimed to find an equilibrium point through the evolutionary game to enable all subjects to actively participate in the transition toward community elderly care and to establish a synergistic framework of multiple subjects of smart elderly care to describe the complex relationships among participants. Additionally, the dynamic evolutionary process of smart community elderly care was inspected from a finite rationality viewpoint, and the evolutionary mechanism of group behavior was elucidated by considering several factors such as strength of implementation, elderly care preference, and level of smartness, providing a theoretical basis for the macro regulation of elderly care and widens the application of evolutionary game in the field of social governance and public health.

The rest of this paper is structured as follows: In Section 2, we review the evolutionary game theory and its applications, and construct the evolutionary game theoretical framework. Then in Section 3, the specific hypotheses and variables are described, while the evolutionary game model matrix is constructed. In Section 4, the stability of the strategies of each game player is outlined. Next, Section 5 simulates the ESS and external parameters with Vensim and possible scenarios. Sections 6 and 7 provide feasible suggestions and short conclusions for managers, respectively.

## 2 Evolutionary game theory and the main players of the game

### 2.1 Evolutionary game and its application

In terms of research framework, game theory has become an effective tool to study the mechanism of synergy, cooperation and to find the optimal strategy, which is extensively used in the field of complex economic, engineering and management science systems [53–55]. As a branch of game theory, evolutionary game, which is an extension of game theory in the study of dynamic conflict, is a theoretical model that combines game theoretical analysis with dynamic evolutionary processes, starting from individuals and taking groups as the object of study [56]. The theory suggests that in reality individuals are not behavioral optimizers, and that decisions are made through a dynamic process of imitation, learning, and mutation between individuals [57]. Compared with traditional game theory, evolutionary game theory is particularly suitable for exploring individual behavioral strategies in large-scale interactive systems and verifying the evolutionary advantage of various strategies over those of competitors [58]. And in reality, the elderly care subject decision-making process facing the incompleteness of information and their own rationality defects, the evolutionary game theory has the advantages of limited rationality assumption and incomplete information, so that it applies to the coordination process of multiple elderly care subjects in the dynamic decision making [59]. On this basis, some scholars have studied the games involved in the senior care process. For example, Zhou et al. constructed a evolutionary game model between the government and private elderly care institution, and then analyzed the policy orientation of supporting private institutions based on the concept of "active ageing" and the policy framework to provide management insights [48]; Li et al. built a senior care game from the perspective of enhancing the resilient development of the emerging senior care technology industry, which effectively matches resource elements to cope with risks internally and maintains the operation of the structure of the emerging elderly care technology industry externally [60]. They also noted the need for further research on elderly care decision making behavior to enrich and reinforce the overall increase in the public value of elderly care services. Therefore, in this study, we use evolutionary game model to study in depth the cooperative and win-win mechanism of multiple subjects in the smart community elderly care system, as well as the influence of its main factors on the evolution of the system, according to which the proposed policy recommendations can effectively stimulate the rapid, healthy and sustainable development of the smart community elderly care industry.

### 2.2 The main players of the game

**Fig 1:**
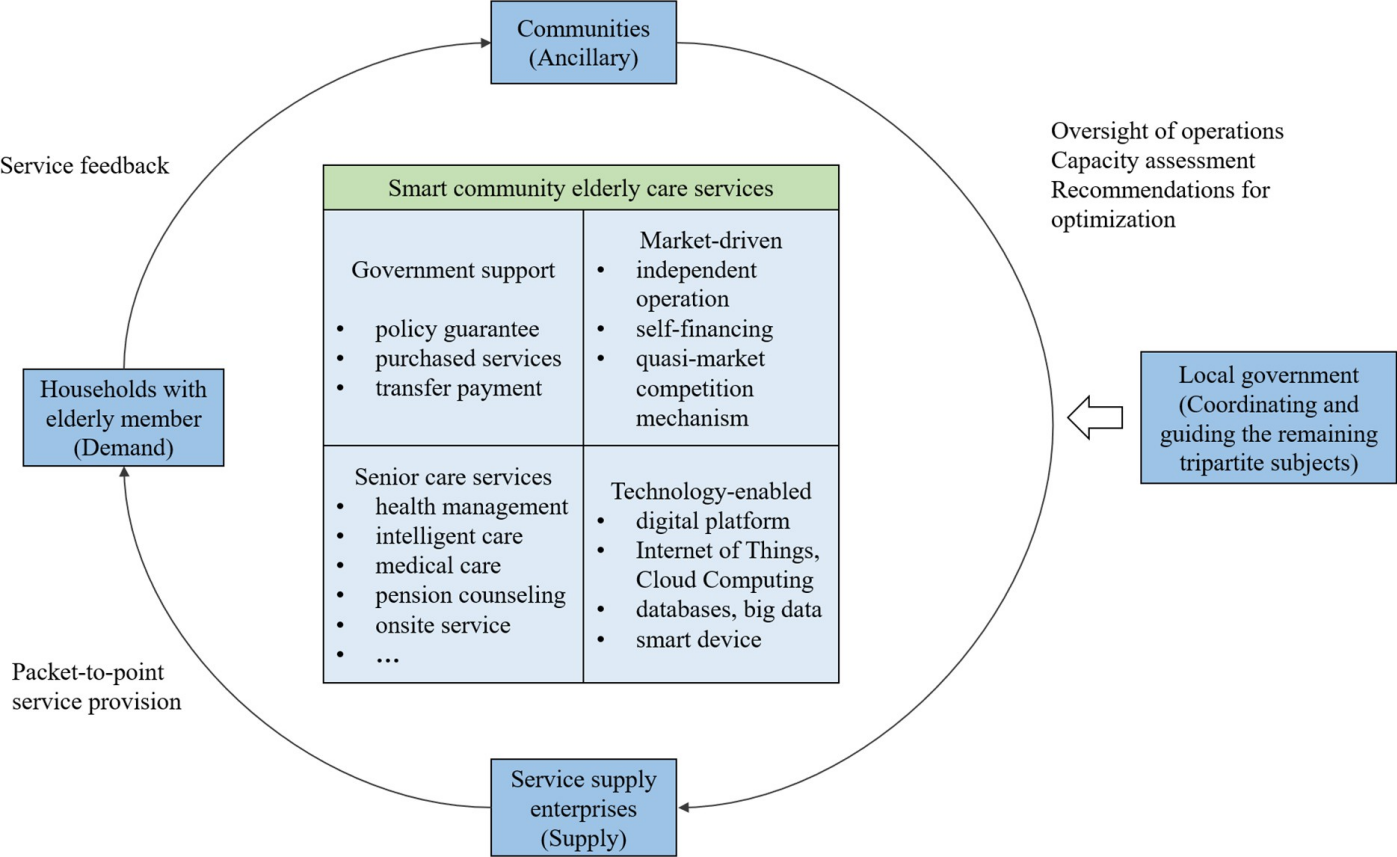
Smart community elderly care service model.

**Fig 2:**
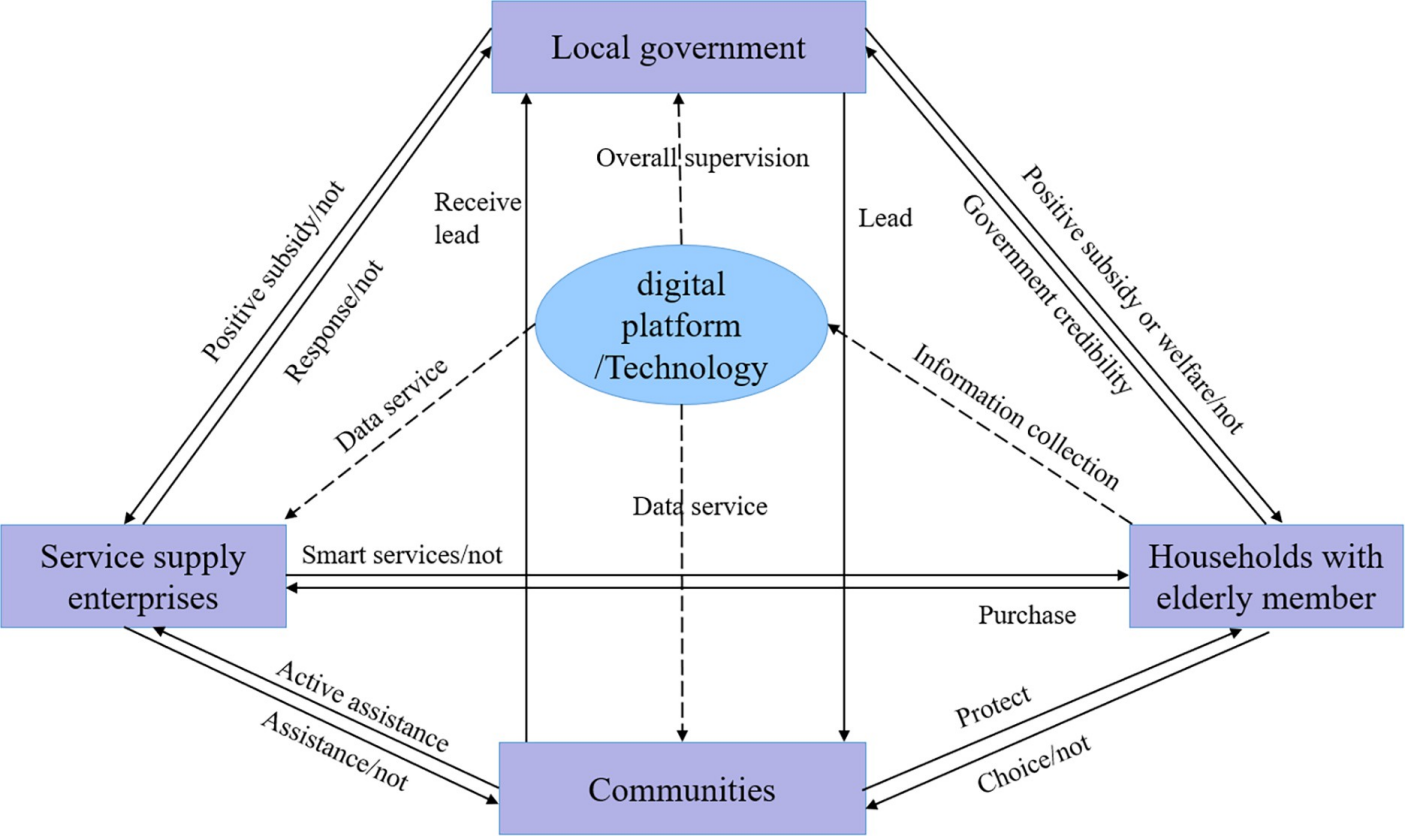
The theoretical framework for the evolutionary game model of smart community elderly care.

An industry or organization can be viewed as a system linked by a multilateral contract between all stakeholders, in the process of operation, a single subject acting alone can hardly achieve the overall Pareto Optimality, and when it comes to multiple stakeholders, the participating subjects will be based on their own status and role in the interest of the game [61]. In smart community elderly services, the stakeholders are complex, including the government, various organizations and the elderly, and so on, which means that the coordination between different stakeholders is quite difficult [62]. According to the different degree of interest involved in each supplying subject, we find that there is a discrepancy between the strength of interest and the level of status of each subject due to the difference in their own attributes. Therefore, combining with previous research [61, 63, 64], we classify smart community elderly care participants into three levels of core stakeholders, sub-core stakeholders, and marginal stakeholders according to Mitchell’s equalization method about the classification and definition of stakeholders, as shown in Table 1. Considering the importance of each subject, in this paper we only discuss the interest game of core stakeholders and sub-core stakeholders, which is the game of local governments, communities, service supply enterprises and households with elderly members. Specifically, in the process of smart community elderly care services, the four main subjects assume different social roles. Elderly people are the demanders and beneficiaries of services, and enterprises are the service suppliers; at the same time, under the macro-policies enacted by the central government to promote elderly care, local governments are the direct recipients of the central policy, playing a top-down role in guiding and incentivizing them; the community’s primary roles are to assist enterprises and households, for example, through community "satisfaction surveys" and "elderly care capacity assessment", to provide feedback to enterprises and governments on the elderly’s view on services, so as to achieve the optimization and upgrading of elderly care. The service model constituted by the four main subjects is shown in Fig 1. Despite the different roles, driven by smart technology, the smart community elderly care services is conducive to the formation of a diversified gathering of subjects with converging interests, and each subject can seek cooperation through close interaction, thus achieving the maximization of individual and organizational goals, and ultimately realizing the optimization and sustainability of smart elderly care services. In doing so, a theoretical framework that simultaneously involves the local government, the community, the service supplying enterprise, and the household with elderly members was developed to describe the relationship between these subjects, as shown in Fig 2.

**Table 1:** Segmentation of stakeholders in the main subject of smart community elderly care.

Stakeholder classification basis	Core stakeholder	Sub-core stakeholders	Marginal stakeholder
**Stakeholder delineation category**	Elderly people and their children in the community	Government, market and service enterprises, community	Social or non-profit organization
**Importance and interest**	Close and direct interest	General close relations	Lower demand, participation and importance of benefits

## 3 Evolutionary game models

### 3.1 Question description

In the quadrilateral gaming process, the local government strategies involve both "positive and negative support". Local governments can create financial and incentive policies to develop smart community elderly care but may not actively provide support policies to the market or may only symbolically implement them. The strategy of the community as a facilitator is "active and passive assistance"; taking active measures to provide the elderly in the community with the necessary supplies for smart services (such as venues), monitoring and giving feedback to service providers, or they are reluctant to invest in the support of the construction of smart community elderly care. As a direct service provider, the strategy of the enterprise is to "provide smart or non-smart services". For smart services, service supply enterprise apply integrated community senior-care platform and accelerate the implementation of big data technology and artificial intelligence to provide smart services through integration and collaboration, thereby providing more efficient and high-quality services. However, enterprises may choose to apply only a basic digital information system, with fewer online services, with a low level of intelligent implementation and low investment in smart facilities. As the direct beneficiaries of the elderly care service, the household with elderly members implements a strategy of "choosing smart and traditional services", which represents the preference and tendency of the elderly and their children to use smart or traditional services.

### 3.2 Model assumptions

**Hypothesis 1**: First, the government’s attitude toward the smart construction of community elderly care is relatively conservative, community assistance is not available, and the smart degree of the elderly care service supply enterprise is low. Although the social development process is currently slow and the service supply enterprise are not highly competitive, as long as social elderly care is still operating, certain benefits can be achieved by the enterprise and other subjects. *G*,*C*,*P* and *E* denote the basic benefits of the local government, community, service supply enterprise, and household with elderly members, respectively.
**Hypothesis 2**: Regarding smart community elderly care, the local government faces the choice of positive or negative support, corresponding to probabilities of *x* and 1—*x*, respectively. Referring to the domestic form of support, let the cost of the government’s infrastructure for the smart elderly care system be *C*_1_. The local government gives a construction subsidy *T*_*p*_ to the community for their active assistance, and the enterprises of high smart services are paid in the form of government-purchased services, which is set as *C*_*s*_. To promote and popularize the participation of elderly groups, the local government implements incentive mechanisms to directly subsidize the elderly to purchase services or indirectly incentivize the elderly to participate in smart community elderly care through the community and enterprise. This government input is considered as *A*_*l*_. And set the support strength of local government as *r*_1_ ∈ [0,1]. In addition, when the local government introduces positive support policies, the proactive response and implementation of the community and the service supply enterprise will create certain social benefits for the government, which are set to *Sb*_*c*_ and *Sb*_*p*_, respectively. With reference to the relevant literature, when local government support is weak, the external development conditions of community and service supply enterprise are relatively immature, the comparatively low rate of return and small number of supply services have not significantly increased the local government’s social benefits relative to the government’s ambitious expenditures on the entire senior care industry [48].
**Hypothesis 3**: The community can choose between active and passive assistance with probabilities *y* and 1—*y*, respectively. The cost of community active implementation, *C*_2_, includes supervision and feedback costs, resource support, and so on, with the degree of implementation coefficient of *r*_2_ ∈ [0,1]. First, through the community’s active assistance in smart elderly care system construction, the willingness of highly smart enterprises to cooperate with the community and the satisfaction of households who choose smart elderly care increase, while generating value-added benefits to the community as *ρRc*_*p*_ and *Rc*_*e*_, *ρ* is the coefficient of the synergy of resource allocation between enterprise and community. Moreover, regardless of the community’s decision to adopt a certain attitude, the highly smart services of the enterprise will enable the community to enjoy additional benefits from the smart services, *R*_*a*_, such as the reputation of the community in the region, the improvement in service quality, and economic benefits. In addition, if the local government is positive while the community is slack, because Chinese community have the obligation to assist government departments in social management, the community will deviate from the government’s administrative direction and hinder the construction of smart elderly care, which will be criticized or even punishment by the government; its negative value perception is −*N*_*g*_.
**Hypothesis 4**: The service supply enterprise can choose to provide highly smart services and low- or non-smart services with probability *z* or 1—*z*, respectively. Assume that the basic service cost of the elderly service enterprise is *C*_3_. Referring to the relevant literature [65], the additional smart cost $\Delta C=q\left(c_{p}+\frac{1}{2}S^{2}\right)$ is invested when the smart level is higher, where *c*_*p*_ is the quantity cost, $\frac{1}{2}S^{2}$ denotes the quality cost, and *S* is the service level of the enterprise. *q* ∈ [0,1] is the smart degree coefficient. Among them, higher degree of smart are reflected in the scale of application of smart hardware and network, the scope of service content, and other "quantity" aspects; and in wearable devices, intelligent monitoring service equipment, and other smart hardware functions, efficiency, service quality, and other "quality" aspects. Higher smartening inputs lead to significant quality improvement and efficiency gain for the enterprise, where the total benefit increase compared with the original model is *qP*_*s*_.
  Moreover, the enterprise gain from adopting smartness is influenced by the synergy of resource allocation between the enterprise and community *ρ*. The synergy gain by the highly smart enterprise with active community assistance is *ρRp*_*a*_; it also has a guiding and promoting effect on the low- or non-smart enterprise, which is *ρRp*_*n*_ (*Rp*_*a*_ > *Rp*_*n*_). Because the synergistic smart gain of the enterprise with passive community assistance is relatively weak, we assumed here that the gain by the service supply enterprise when they form a weak synergistic relationship with the community is zero.
**Hypothesis 5**: The probability that the household with elderly members choose smart or traditional when selecting community elderly care services is *w* and 1—*w*, respectively. Suppose *α*(0 < *α* < 1) is the sensitivity of households with elderly members to the service level, where a larger *α* indicates the poorer health of the elderly. *L* is the basic service payment for households; *β*(0 < *β* < 1) indicates the price sensitivity, where a larger *β* means a weaker ability to pay. Then, (1 - *β*) represents the proportion of households willing to pay more for additional smart because of the more valuable services generated by additional smart. When the household choose smart elderly care, *θ* and *γ* represent the proportion of the household gaining additional health benefits because of the value of service efficiency enhancement, convenience, and diversity brought by favorable community environment and high smart services [66]. On the contrary, households with preferences for traditional services are not willing to pay more and therefore do not use smart services.
**Hypothesis 6**: Households’ preference will also contribute to the choice of supply-side strategies, so we set the perceived value of community and enterprise for household preferences as *Nc*_*s*_, *Nc*_*t*_ and *Np*_*s*_, *Np*_*t*_, respectively, where *s* denotes smart preference, and *t* denotes traditional preference. When household preferences are consistent with the supply-side strategy (that is, the community and enterprise actively participate in smart construction and households prefer smart services; or when the community and enterprise negatively treat smart construction and households prefer traditional services), the supply side is incentivized with increased household loyalty in both of the above two cases. On the contrary, when preferences and supply-side strategies are not aligned, this leads to negative effect due to a series of negative feedbacks such as declining ratings and loss of potential customers. Moreover, because smartness is the mainstream trend in future social development, and the current market share of smart services is quickly rising, so here the perception of smart preference by the community and enterprise is higher than the perception of traditionalization preference, which is *Nc*_*s*_ > *Nc*_*t*_, *Np*_*s*_ > *Np*_*t*_.

As mentioned above, we can obtain the payoff matrix for the quadrilateral subjects, as shown in Tables 2 and 3. For convenience, let *A*,*B*,*C* and *D* correspond to the local government, community, service supply enterprise and household in the payment matrix, respectively. Meanwhile, the four strategy combinations are counted as numbered 1–16 in order according to the payment matrix, where the household with smart preference (Table 2) is recorded as odd and the household with traditional preference (Table 3) is recorded as even.

**Table 2:** Payoff matrix when household is smart preference.

				C	
				Provide highly smart services	Provide low- or non-smart services
**A**	**Positive**	**B**	**Active**	*G* − *C*_1_ − *T*_*p*_ − *C*_*s*_ − *A*_*l*_ + *Sb*_*c*_ + *Sb*_*p*_	*G* − *C*_1_ − *T*_*p*_ − *A*_*l*_ + *Sb*_*c*_
				*C* − *C*_2_ + *T*_*p*_ + *ρRc*_*p*_ + *Rc*_*e*_ + *R*_*a*_ + *Nc*_*s*_	*C* − *C*_2_ + *T*_*p*_ − *Nc*_*t*_
				*P* + *C*_*s*_ − *C*_3_ − *ΔC* + *qP*_*s*_ + *ρRp*_*a*_ + *Np*_*s*_	*P* − *C*_3_ + *ρRp*_*n*_ − *Np*_*s*_
				*E*(1 + *θ* + *γ*) + *αS*(1 + *q*) − *L*(1 + 1 − *β*) + *A*_*l*_	*E*(1 + *θ*) + *αS* − *L*(1 + 1−*β*)*A*_*l*_
			**Passive**	*G* − *C*_1_ − *C*_*S*_ − *A*_*l*_ + *Sb*_*p*_	*G* − *C*_1_ − *A*_*l*_
				*C* − *r*_2_ *C*_2_ + *R*_*a*_ − *N*_*g*_ − *Nc*_*s*_	*C* − *r*_2_ *C*_2_ − *N*_*g*_ − *Nc*_*s*_
				*P* + *C*_*s*_ − *C*_3_ − *ΔC* + *qP*_*s*_ + *Np*_*s*_	*P* − *C*_3_ − *Np*_*s*_
				*E*(1 + *γ*) + *αS*(1 + *q*) − *L*(1 + 1 − *β*) + *A*_*l*_	*E* + *αS* − *L*(1 + 1 − *β*) + *A*_*l*_
	**Negative**	**B**	**Active**	*G* − *C*_1_ − *r*_1_ (*T*_*p*_ + *C*_*S*_ + *A*_*l*_)	*G* − *C*_1_ − *r*_1_ (*T*_*p*_ + *A*_*l*_)
				*C* − *C*_2_ + *r*_1_ *T*_*p*_ + *ρRc*_*p*_ + *Rc*_*e*_ + *R*_*a*_ + *Nc*_*s*_	*C* − *C*_2_ + *r*_1_ *T*_*p*_ + *Rc*_*e*_ + *Nc*_*s*_
				*P* + *r*_1_ *C*_*s*_ − *C*_3_ − *ΔC* + *qP*_*s*_ + *ρRp*_*a*_ + *Np*_*s*_	*P* − *C*_3_ + *ρRp*_*n*_ − *Np*_*s*_
				*E*(1 + *θ* + *γ*) + *αS*(1 + *q*) − *L*(1 + 1 − *β*) + *r*_1_ *A*_*l*_	*E*(1 + *θ*) + *αS* − *L*(1 + 1 − *β*) + *A*_*l*_
			**Passive**	*G* − *C*_1_ − *r*_1_ (*C*_*S*_ + *A*_*l*_)	*G* − *C*_1_ − *r*_1_ *A*_*l*_
				*C* − *r*_2_ *C*_2_ + *R*_*a*_ − *Nc*_*s*_	*C* − *r*_2_ *C*_2_ − *Nc*_*s*_
				*P* + *r*_1_ *C*_*s*_ − *C*_3_ − *ΔC* + *qP*_*s*_ + *Np*_*s*_	*P* − *C*_3_ − *Np*_*s*_
				*E*(1 + *γ*) + *αS*(1 + *q*) − *L*(1 + 1 − *β*) + *r*_1_ *A*_*l*_	*E* + *αS* − *L*(1 + 1 − *β*) + *r*_1_ *A*_*l*_

**Table 3:** Payoff matrix when household is traditional preference.

				C	
				Provide highly smart services	Provide low- or non-smart services
**A**	**Positive**	**B**	**Active**	*G* − *C*_1_ − *T*_*p*_ − *C*_*S*_ + *Sb*_*c*_ + *Sb*_*p*_	*G* − *C*_1_ − *T*_*p*_ + *Sb*_*c*_
				*C* − *C*_2_ + *T*_*p*_ + *ρRc*_*p*_ + *R*_*a*_ − *Nc*_*t*_	*C* − *C*_2_ + *T*_*p*_ − *Nc*_*t*_
				*P* + *C*_s_ − *C*_3_ − *ΔC* + *qP*_*s*_ + *ρRp*_*a*_ − *Np*_*t*_	*P* − *C*_3_ + *ρRp*_*n*_ + *Np*_*t*_
				*E* + *αS* − *L*	*E* + *αS* − *L*
			**Passive**	*G* − *C*_1_ − *C*_*S*_ + *Sb*_*p*_	*G* − *C*_1_
				*C* − *r*_2_ *C*_2_ + *R*_*a*_ − *N*_*g*_ + *Nc*_*t*_	*C* − *r*_2_ *C*_2_ − *N*_*g*_ + *Nc*_*t*_
				*P* + *C*_*s*_ − *C*_3_ − *ΔC* + *qP*_*s*_ − *Np*_*t*_	*P* − *C*_3_ + *Np*_*t*_
				*E* + *αS* − *L*	*E* + *αS* − *L*
	**Negative**	**B**	**Active**	*G* − *C*_1_ − *r*_1_ (*Tp* + *C*_*S*_)	*G* − *C*_1_ − *r*_1_ *T*_*p*_
				*C* − *C*_2_ + *r*_1_ *T*_*p*_ + *ρRc*_*p*_ + *R*_*a*_ − *Nc*_*t*_	*C* − *C*_2_ + *r*_2_ *T*_*p*_ − *Nc*_*t*_
				*P* + *r*_1_ *C*_s_ − *C*_3_ − *ΔC* + *qP*_*s*_ + *ρRp*_*a*_ − *Np*_*t*_	*P* − *C*_3_ + *ρRp*_*n*_ + *Np*_*t*_
				*E* + *αS* − *L*	*E* + *αS* − *L*
			**Passive**	*G* − *C*_1_ − *r*_1_ *C*_*s*_	*G* − *C*_1_
				*C* − *r*_2_ *C*_2_ + *R*_*a*_ + *Nc*_*t*_	*C* − *r*_2_ *C*_2_ + *Nc*_*t*_
				*P* + *r*_1_ *C*_*s*_ − *C*_3_ − *ΔC* + *qP*_*s*_ − *Np*_*t*_	*P* − *C*_3_ + *Np*_*t*_
				*E* + *αS* − *L*	*E* + *αS* − *L*

### 3.3 Dynamic evolution results analysis

#### 3.3.1 Evolutionary stable strategy of the local government

The expected payoff of the local government choosing positive and negative support are *U*_*A*1_ and *U*_*A*2_. Then, the average expected payoff is *U*_*A*_.


$$U_{A1}=yzwa_{1}+yz(1-w)a_{2}+y(1-z)wa_{3}+y(1-z)(1-w)a_{4}+(1-y)zwa_{5}+(1-y)z(1-w)a_{6}+(1-y)(1-z)wa_{7}+(1-y)(1-z)(1-w)a_{8}$$ (1)

$$U_{A2}=yzwa_{9}+yz(1-w)a_{10}+y(1-z)wa_{11}+y(1-z)(1-w)a_{12}+(1-y)zwa_{13}+(1-y)z(1-w)a_{14}+(1-y)(1-z)wa_{15}+(1-y)(1-z)(1-w)a_{16}$$ (2)

$$U_{A}=xU_{A1}+(1-x)U_{A2}$$ (3)

Therefore, the dynamic evolution equation of the local government is:

$$\begin{array}{c} F(x)=\frac{dx}{dt}=x\left(U_{A1}-U_{A}\right)\\ =x(1-x)\left[\left(Sb_{c}-T_{P}+r_{1}T_{P}\right)y+\left(Sb_{P}-C_{S}+r_{1}C_{S}\right)z+\left(-A_{L}+r_{1}A_{L}\right)w\right] \end{array}$$ (4)

$$\frac{d(F(x))}{dx}=(1-2x)\left[\left(Sb_{c}-T_{P}+r_{1}T_{P}\right)y+\left(Sb_{P}-C_{S}+r_{1}C_{S}\right)z+\left(-A_{L}+r_{1}A_{L}\right)w\right]$$ (5)

According to the theorem of differential equation stability, *x* is the stability point of the local government’s strategy when the probability *x* of local government choosing positive support satisfies *F*(*x*) = 0 and *F*′ (*x*) < 0. The same applies to the remaining three subjects. Next, we discuss the influence of the probability *y* of active assistance by the community on the local government’s strategy choice.

Let $G(y,z,w)=\left(Sb_{c}-T_{P}+r_{1}T_{P}\right)y+\left(Sb_{P}-C_{S}+r_{1}C_{S}\right)z+\left(-A_{L}+r_{1}A_{L}\right)w$. From G(y,z,w)=0, we can get: when $y=\frac{\left(Sb_{P}-C_{S}+r_{1}C_{S}\right)z+\left(-A_{L}+r_{1}A_{L}\right)w}{T_{P}-Sb_{c}-r_{1}T_{P}}$, $\frac{d(F(x))}{dx}\equiv 0$; when $y\neq \frac{\left(Sb_{P}-C_{S}+r_{1}C_{S}\right)z+\left(-A_{L}+r_{1}A_{L}\right)w}{T_{P}-Sb_{c}-r_{1}T_{P}}$, let *F*(*x*) = 0, then *x* = 0 and *x* = 1 are two equilibrium points, so we need to discuss them in categories.

**Proposition 1**: If the probability of the community choosing the active assistance strategy is higher than $\frac{\left(Sb_{P}-C_{S}+r_{1}C_{S}\right)z+\left(-A_{L}+r_{1}A_{L}\right)w}{T_{P}-Sb_{c}-r_{1}T_{P}}$, then the probability that the local government chooses to positive support stabilizes at 1. Conversely, if the probability of the community choosing the active assistance strategy is less than $\frac{\left(Sb_{P}-C_{S}+r_{1}C_{S}\right)z+\left(-A_{L}+r_{1}A_{L}\right)w}{T_{P}-Sb_{c}-r_{1}T_{P}}$, then the probability that the local government selects positive support stabilizes at 0.

**Proof**: $\frac{d(G(y,z,w))}{dy}=Sb_{c}-T_{P}+r_{1}T_{P}>0$; therefore, *G*(*y*,*z*,*w*) is an increasing function with respect to *y*. When $y>\frac{\left(Sb_{P}-C_{S}+r_{1}C_{S}\right)z+\left(-A_{L}+r_{1}A_{L}\right)w}{T_{P}-Sb_{c}-r_{1}T_{P}}$, *G*(*y*,*z*,*w*) > 0, and hence there are ${\left.\frac{d(F(x))}{dx}\right|}_{x=0}>0$, ${\left.\frac{d(F(x))}{dx}\right|}_{x=1}<0$. At this point, the local government chooses to positive support strategy. When $y<\frac{\left(Sb_{P}-C_{S}+r_{1}C_{S}\right)z+\left(-A_{L}+r_{1}A_{L}\right)w}{T_{P}-Sb_{c}-r_{1}T_{P}}$, *G*(*y*,*z*,*w*) < 0, then ${\left.\frac{d(F(x))}{dx}\right|}_{x=0}<0$, ${\left.\frac{d(F(x))}{dx}\right|}_{x=1}>0$. At this point, the local government chooses a negative support strategy.

Proposition 1 suggests that when the probability of active community assistance decreases, the local government are more inclined to adopt a conservative fiscal policy and negative support. When the probability of active community assistance increases, the local government changes from negative to positive support. At this time, if the local government still adopts negative support, although it relieves a certain financial burden in the short term, it will place pressure on people’s livelihoods and elderly care and will face the risk of lagging behind in the construction of smart urban services, which is not worth the loss. Therefore, with the gradual advancement of the smart development process and the increasing willingness of the participants to engage a healthier and more advanced way of elderly care, positive support will become the mainstream strategy of the local government.

In summary, the response function for the local government’s choice of strategy *x* is:

$$x=\left\{\begin{array}{cc} 0 & if\;y<\frac{\left(Sb_{P}-C_{S}+r_{1}C_{S}\right)z+\left(-A_{L}+r_{1}A_{L}\right)w}{T_{P}-Sb_{c}-r_{1}T_{P}}\\ \left[0,1\right] & if\;y=\frac{\left(Sb_{P}-C_{S}+r_{1}C_{S}\right)z+\left(-A_{L}+r_{1}A_{L}\right)w}{T_{P}-Sb_{c}-r_{1}T_{P}}\\ 1 & if\;y>\frac{\left(Sb_{P}-C_{S}+r_{1}C_{S}\right)z+\left(-A_{L}+r_{1}A_{L}\right)w}{T_{P}-Sb_{c}-r_{1}T_{P}} \end{array}\right.$$ (6)

**Fig 3:**
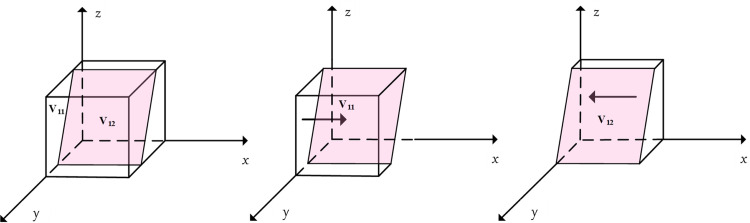
Phase diagram of the evolution of local government strategies.

According to Proposition 1, the phase diagram of positive support strategies chosen by the local government was drawn and is shown in Fig 3, where *V*_11_ represents the probability of *x* = 1 and *V*_12_ represents the probability of *x* = 0.

#### 3.3.2 Evolutionary stable strategy of the community

The expected payoff for the community choosing active and passive assistance are *U*_*B*1_ and *U*_*B*2_; the average expected payoff is *U*_*B*_. Then,

$$U_{B1}=xzwb_{1}+xz(1-w)b_{2}+x(1-z)wb_{3}+x(1-z)(1-w)b_{4}+(1-x)zwb_{9}+(1-x)z(1-w)b_{10}+(1-x)(1-z)wb_{11}+(1-x)(1-z)(1-w)b_{12}$$ (7)

$$U_{B2}=xzwb_{5}+xz(1-w)b_{6}+x(1-z)wb_{7}+x(1-z)(1-w)b_{8}+(1-x)zwb_{13}+(1-x)z(1-w)b_{14}+(1-x)(1-z)wb_{15}+(1-x)(1-z)(1-w)b_{16}$$ (8)

$$U_{B}=yU_{B1}+(1-y)U_{B2}$$ (9)

Therefore, the dynamic evolution equation for the community is:

$$\begin{array}{c} F(y)=\frac{dy}{dt}=y\left(U_{B1}-U_{B}\right)=y(1-y)\left(U_{B1}-U_{B2}\right)\\ =y(1-y)\left[\left(2Nc_{s}+2Nc_{t}+Rc_{e}\right)w+\left(N_{g}+T_{P}-r_{1}T_{P}\right)x+\rho Rc_{P}z+r_{2}C_{2}-2Nc_{t}-C_{2}+r_{1}T_{P}\right] \end{array}$$ (10)

$$\left.\frac{d(F(y))}{dy}\right|=(1-2y)\left[\left(2Nc_{s}+2Nc_{t}+Rc_{e}\right)w+\left(N_{g}+T_{P}-r_{1}T_{P}\right)x+\rho Rc_{P}z+r_{2}C_{2}-2Nc_{t}-C_{2}+r_{1}T_{P}\right]$$ (11)

Next, we discuss the impact of changes in the probability *w* of choosing smart services for the household with elderly members on the choice of community strategy.

Let $G(x,z,w)=\left(2Nc_{s}+2Nc_{t}+Rc_{e}\right)w+\left(N_{g}+T_{P}-r_{1}T_{P}\right)x+\rho Rc_{P}z+r_{2}C_{2}-2Nc_{t}-C_{2}+r_{1}T_{P}.$ From *G*(*x*,*z*,*w*) = 0, when $w=\frac{\left(N_{g}+T_{P}-r_{1}T_{P}\right)x+\rho Rc_{P}z+r_{2}C_{2}-2Nc_{t}-C_{2}+r_{1}T_{P}}{-2Nc_{s}-2Nc_{t}-Rc_{e}},$ $\frac{d(F(y))}{dy}\equiv 0$; when $w\neq \frac{\left(N_{g}+T_{P}-r_{1}T_{P}\right)x+\rho Rc_{P}z+r_{2}C_{2}-2Nc_{t}-C_{2}+r_{1}T_{P}}{-2Nc_{s}-2Nc_{t}-Rc_{e}},$ let *F*(*y*) = 0, then *y* = 0 and *y* = 1 are two equilibrium points, so we need to discuss them in categories.*G*(*x*,*z*,*w*) = 0

**Proposition 2**: If the probability of the household opting for smart services is higher than $\frac{\left(N_{g}+T_{P}-r_{1}T_{P}\right)x+\rho Rc_{P}z+r_{2}C_{2}-2Nc_{t}-C_{2}+r_{1}T_{P}}{-2Nc_{s}-2Nc_{t}-Rc_{e}}$, then the probability of the community opting for active assistance stabilizes at 1. Instead, if the probability that an elderly household opting for smart services is lower than $\frac{\left(N_{g}+T_{P}-r_{1}T_{P}\right)x+\rho Rc_{P}z+r_{2}C_{2}-2Nc_{t}-C_{2}+r_{1}T_{P}}{-2Nc_{s}-2Nc_{t}-Rc_{e}}$, then the probability that the community chooses active assistance stabilizes at 0.

**Proof**: $\frac{dG(x,z,w)}{dw}=2Nc_{s}+2Nc_{t}+Rc_{e}>0$; therefore, *G*(*x*,*z*,*w*) is an increasing function with respect to *w*. When $w>\frac{\left(N_{g}+T_{P}-r_{1}T_{P}\right)x+\rho Rc_{P}z+r_{2}C_{2}-2Nc_{t}-C_{2}+r_{1}T_{P}}{-2Nc_{s}-2Nc_{t}-Rc_{e}}$, *G*(*x*,*z*,*w*) > 0, so ${\left.\frac{d(F(y))}{dy}\right|}_{y=0}>0$, ${\left.\frac{d(F(y))}{dy}\right|}_{y=1}<0$. The community chooses an active assistance strategy at this time. When $w<\frac{\left(N_{g}+T_{P}-r_{1}T_{P}\right)x+\rho Rc_{P}z+r_{2}C_{2}-2Nc_{t}-C_{2}+r_{1}T_{P}}{-2Nc_{s}-2Nc_{t}-Rc_{e}}$, *G*(*x*,*z*,*w*) < 0, there are ${\left.\frac{d(F(y))}{dy}\right|}_{y=0}<0$, ${\left.\frac{d(F(y))}{dy}\right|}_{y=1}>0$. At this point, the community chooses passive assistance strategy.

Proposition 2 suggests that when the demand for community smart elderly care from households increases and the willingness to be smart improves, the increased perception of value promotes a shift in community decision making from passive to active assistance. If the household’s willingness to adopt smart services is weaker and they are less receptive to new things, the community resists smart construction, and decision making changes from active to passive assistance.

In summary, the response function for the community’s choice of strategy *y* is:

$$y=\left\{\begin{array}{cc} 0 & if\;w<\frac{\left(N_{g}+T_{P}-r_{1}T_{P}\right)x+\rho Rc_{p}z+r_{2}C_{2}-2Nc_{t}-C_{2}+r_{1}T_{P}}{-2Nc_{s}-2Nc_{t}-Rc_{e}}\\ \left[0,1\right] & if\;w=\frac{\left(N_{g}+T_{p}-r_{1}T_{p}\right)x+\rho Rc_{p}z+r_{2}C_{2}-2Nc_{t}-C_{2}+r_{1}T_{p}}{-2Nc_{s}-2Nc_{t}-Rc_{e}}\\ 1 & if\;w>\frac{\left(N_{g}+T_{P}-r_{1}T_{P}\right)x+\rho Rc_{p}z+r_{2}C_{2}-2Nc_{t}-C_{2}+r_{1}T_{p}}{-2Nc_{s}-2Nc_{t}-Rc_{e}} \end{array}\right.$$ (12)

**Fig 4:**
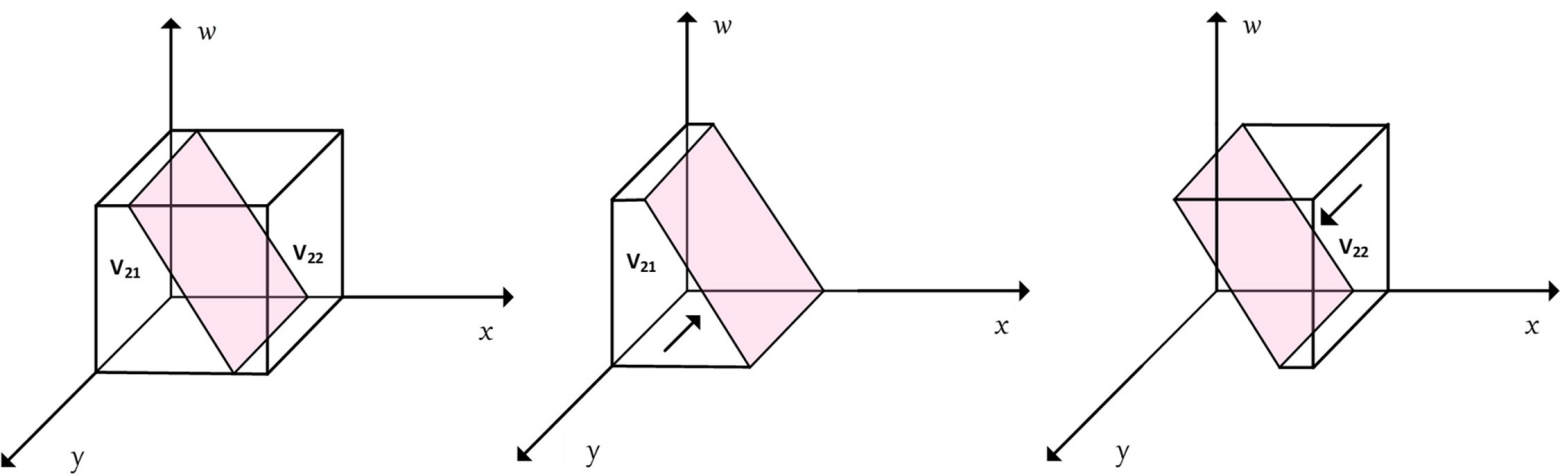
Phase diagram of the evolution of community strategies.

According to Proposition 2, the phase diagram of active assistance strategies chosen by the community was drawn and is shown in Fig 4, where *V*_21_ represents the probability of *y* = 0, and *V*_22_ represents the probability of *y* = 1.

#### 3.3.3 Evolutionary stable strategy of the service supply enterprise

Let the expected payoff of a service supply enterprise with high smart be *U*_*C*1_ and the expected payoff of an enterprise with low- or non- smart be *U*_*C*2_. The average expected payoff is *U*_*C*_, then

$$U_{C1}|=xywc_{1}+xy(1-w)c_{2}+x(1-y)wc_{5}+x(1-y)(1-w)c_{6}+(1-x)ywc_{9}+(1-x)y(1-w)c_{10}+(1-x)(1-y)wc_{13}+(1-x)(1-y)(1-w)c_{14}$$ (13)

$$U_{C2}=xywc_{3}+xy(1-w)c_{4}+x(1-y)wc_{7}+x(1-y)(1-w)c_{8}+(1-x)ywc_{11}+(1-x)y(1-w)c_{12}+(1-x)(1-y)wc_{15}+(1-x)(1-y)(1-w)c_{16}$$ (14)

$$U_{C}=zU_{C1}+(1-z)U_{C2}$$ (15)

Therefore, the dynamic evolution equation of the service supply enterprise can be written as:

$$F(z)=\frac{dz}{dt}=z\left(U_{C1}-U_{C}\right)=z(1-z)\left(U_{C1}-U_{C2}\right)=z(1-z)\left[\left(C_{s}-r_{1}C_{s}\right)x+\left(pRp_{a}-pRp_{n}\right)y+\left(2Np_{s}+2Np_{t}\right)w+r_{1}C_{s}-q\left(C_{p}+\frac{1}{2}S^{2}-P_{s}\right)-2Np_{t}\right]$$ (16)

$$\frac{d(F(z))}{dz}=(1-2z)\left[\left(C_{s}-r_{1}C_{s}\right)x+\left(pRp_{a}-pRp_{n}\right)y+\left(2Np_{s}+2Np_{t}\right)w+r_{1}C_{s}-q\left(C_{p}+\frac{1}{2}S^{2}-P_{s}\right)-2Np_{t}\right]$$ (17)

Then, we discuss the impact of the change in the probability *w* of choosing smart services for the household with elderly members on the strategic choices of service supply enterprise.

We have $G(x,y,w)=\left(C_{s}-r_{1}C_{s}\right)x+\left(pRp_{a}-pRp_{n}\right)y+\left(2Np_{s}+2Np_{t}\right)w+r_{1}C_{s}-q\left(C_{p}+\frac{1}{2}S^{2}-P_{s}\right)-2Np_{t}.$ From *G*(*x*,*y*,*w*) = 0, we conclude that when $w=\frac{\left(C_{s}-r_{1}C_{s}\right)x+\left(pRp_{a}-pRp_{n}\right)y+r_{1}C_{s}-q\left(C_{p}+\frac{1}{2}S^{2}-P_{s}\right)-2Np_{t}}{-2Np_{s}-2Np_{t}}$, $\frac{d(F(z))}{dz}\equiv 0$. When $w\neq \frac{\left(C_{s}-r_{1}C_{s}\right)x+\left(pRp_{a}-pRp_{n}\right)y+r_{1}C_{s}-q\left(C_{p}+\frac{1}{2}S^{2}-P_{s}\right)-2Np_{t}}{-2Np_{s}-2Np_{t}}$, let *F*(*z*) = 0, then *z* = 0 and *z* = 1 are two equilibrium points, so we need to categorize the discussion.

**Proposition 3**: If the probability of the household with elderly members preferring smart services is higher than $\frac{\left(C_{s}-r_{1}C_{s}\right)x+\left(pRp_{a}-pRp_{n}\right)y+r_{1}C_{s}-q\left(C_{p}+\frac{1}{2}S^{2}-P_{s}\right)-2Np_{t}}{-2Np_{s}-2Np_{t}}$, then the service supply enterprise’s probability of providing highly smart services stabilizes at 1. If the probability of the household tending toward smart services is lower than $\frac{\left(C_{s}-r_{1}C_{s}\right)x+\left(pRp_{a}-pRp_{n}\right)y+r_{1}C_{s}-q\left(C_{p}+\frac{1}{2}S^{2}-P_{s}\right)-2Np_{t}}{-2Np_{s}-2Np_{t}}$, the probability of the service supply enterprise providing highly smart services stabilizes at 0.

**Proof**: Because $\frac{dG(x,y,w)}{dw}=2Np_{s}+2Np_{t}>0$, *G*(*x*,*y*,*w*) is an increasing function with respect to *w*. When $w<\frac{\left(C_{s}-r_{1}C_{s}\right)x+\left(pRp_{a}-pRp_{n}\right)y+r_{1}C_{s}-q\left(C_{p}+\frac{1}{2}S^{2}-P_{s}\right)-2Np_{t}}{-2Np_{s}-2Np_{t}}$, *G*(*x*,*y*,*w*) > 0, so ${\left.\frac{d(F(z))}{dz}\right|}_{z=0}<0$, ${\left.\frac{d(F(z))}{dz}\right|}_{z=1}>0$. Similarly, when *w* is greater than the above threshold, *G*(*x*,*y*,*w*) < 0; at this time ${\left.\frac{d(F(z))}{dz}\right|}_{z=0}>0$, ${\left.\frac{d(F(z))}{dz}\right|}_{z=1}<0.$ Similar to Proposition 2, Proposition 3 shows that if the household tends to have a strong smart service, then the service supply enterprise tends to provide smarter services, and their decisions are closely related to the decision-making behavior of the local government and community. Conversely, if the household prefers traditional services, then the enterprise also tries to provide more traditional services. This confirms once again that that the household’s preference plays a role in other subjects’ decision.

Additionally, the response function of the enterprise choosing a highly smart service strategy can be expressed as:

$$z=\left\{\begin{array}{cc} 0 & if\;w<\frac{\left(C_{s}-r_{1}C_{s}\right)x+\left(pRp_{a}-pRp_{n}\right)y+r_{1}C_{s}-q\left(C_{p}+\frac{1}{2}S^{2}-P_{s}\right)-2Np_{t}}{-2Np_{s}-2Np_{t}}\\ \left[0,1\right] & if\;w=\frac{\left(C_{s}-r_{1}C_{s}\right)x+\left(pRp_{a}-pRp_{n}\right)y+r_{1}C_{s}-q\left(C_{p}+\frac{1}{2}S^{2}-P_{s}\right)-2Np_{t}}{-2Np_{s}-2Np_{t}}\\ 1 & if\;w>\frac{\left(C_{s}-r_{1}C_{s}\right)x+\left(pRp_{a}-pRp_{n}\right)y+r_{1}C_{s}-q\left(C_{p}+\frac{1}{2}S^{2}-P_{s}\right)-2Np_{t}}{-2Np_{s}-2Np_{t}} \end{array}\right.$$ (18)

**Fig 5:**
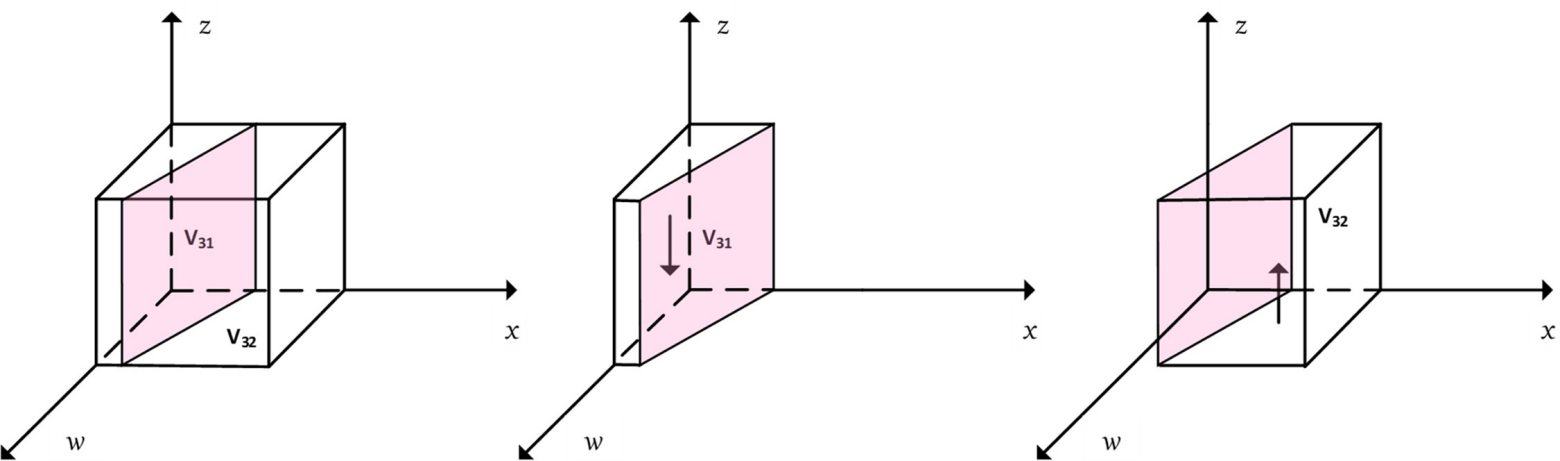
Phase diagram of the evolution of service supply enterprises’ strategies.

According to Proposition 3, the phase diagram of choosing a highly smart service strategy by enterprises was drawn and is shown in Fig 5, where *V*_31_ represents the probability of *z* = 0, and *V*_32_ represents the probability of *z* = 1.

#### 3.3.4 Evolutionary stable strategy of the household with elderly members

The expected payoff of the household with elderly members choosing smart or low- or non-smart services are *U*_*D*1_ and *U*_*D*2_, respectively; and the average expected payoff is *U*_*D*_. Then,

$$U_{D1}=xyzd_{1}+x(1-y)zd_{5}+(1-x)yzd_{9}+(1-x)(1-y)zd_{13}+xy(1-z)d_{3}+x(1-y)(1-z)d_{7}+(1-x)z(1-y)d_{11}+(1-x)(1-y)(1-z)d_{15}$$ (19)

$$U_{D2}|=xyzd_{2}+x(1-y)zd_{6}+(1-x)yzd_{10}+(1-x)(1-y)zd_{14}+xy(1-z)d_{4}+x(1-y)(1-z)d_{8}+(1-x)z(1-y)d_{12}+(1-x)(1-y)(1-z)d_{16}$$ (20)

$$U_{D}=mU_{D1}+(1-m)U_{D2}$$ (21)

Therefore, the dynamic evolution equation of the household can be written as:

$$F(w)=w(1-w)\left[\left(A_{L}-r_{1}A_{L}\right)x+\theta Ey+(\gamma E+aqS)z+\beta L-L+r_{1}A_{L}\right]$$ (22)

$$\frac{d(F(w))}{dw}=(1-2w)\left[\left(A_{L}-r_{1}A_{L}\right)x+\theta Ey+(\gamma E+aqS)z+\beta L-L+r_{1}A_{L}\right]$$ (23)

Let $G(x,y,z)=\left(A_{L}-r_{1}A_{L}\right)x+\theta Ey+(\gamma E+aqS)z+\beta L-L+r_{1}A_{L}$. From *G*(*x*,*y*,*z*) = 0, we conclude that when $z=\frac{\left(A_{L}-r_{1}A_{L}\right)x+\theta Ey+\beta L-L+r_{1}A_{L}}{-\gamma E-aqS}$, $\frac{d(F(w))}{dw}\equiv 0$; when $z\neq \frac{\left(A_{L}-r_{1}A_{L}\right)x+\theta Ey+\beta L-L+r_{1}A_{L}}{-\gamma E-aqS}$, let *F*(*z*) = 0, then *w* = 0 and *w* = 1 are two equilibrium points, thus we need to categorize and discuss two situations.

If the probability of the service supply enterprise choosing to provide highly smart services is higher than $\frac{\left(A_{L}-r_{1}A_{L}\right)x+\theta Ey+\beta L-L+r_{1}A_{L}}{-\gamma E-aqS}$, then the probability of the elderly household choosing smart services stabilizes at 1. Conversely, if the probability is lower than the above threshold, then the probability of the elderly household choosing smart services stabilizes at 0. The proof is the same as the above so will not be repeated here.

Proposition 4 indicates that when the strategic probability of the service supply enterprise choosing to respond positively to the construction of smart services increases, the best strategic choice for the household is to actively participate in smart services at this time to receive better health services. This is corroborated by the findings of the analysis of the evolutionary stability of the service supply enterprise. In turn, they will shift from engaging in smart to traditional elderly care.

At this point, the response function of the household choosing a smart community elderly care strategy can be expressed as:

$$w=\left\{\begin{array}{cc} 0 & if\;z<\frac{\left(A_{L}-r_{1}A_{L}\right)x+\theta Ey+\beta L-L+r_{1}A_{L}}{-\gamma E-aqS}\\ \left[0,1\right] & if\;z=\frac{\left(A_{L}-r_{1}A_{L}\right)x+\theta Ey+\beta L-L+r_{1}A_{L}}{-\gamma E-aqS}\\ 1 & if\;z>\frac{\left(A_{L}-r_{1}A_{L}\right)x+\theta Ey+\beta L-L+r_{1}A_{L}}{-\gamma E-aqS} \end{array}\right.$$ (24)

**Fig 6:**
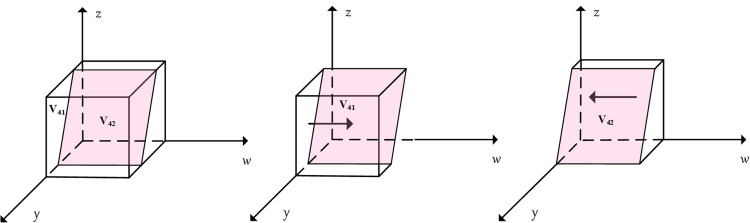
Phase diagram of the evolution of household with elderly members’ strategies.

Thus, the evolutionary dynamics and evolutionary stabilization strategy of the elderly household are shown in Fig 6, where *V*_41_ is the probability of the household choosing *w* = 1, and *V*_42_ is the probability of the household choosing *w* = 0.

## 4 System stability analysis

A set of equations for the replicated dynamic consisting of local government, community, service supply enterprise, and household with elderly members. Making this system of equations 0 yields 16 pure strategic equilibrium points for the replicated dynamic system, that is, *E*_1_(0,0,0,0), *E*_2_(0,0,0,1), *E*_3_(0,0,1,0), *E*_4_(0,0,1,1), *E*_5_(0,1,0,0), *E*_6_(0,1,0,1), *E*_7_(0,1,1,0), *E*_8_(0,1,1,1), *E*_9_(1,0,0,0), *E*_10_(1,0,0,1), *E*_11_(1,0,1,0), *E*_12_(1,0,1,1), *E*_13_(1,1,0,0), *E*_14_(1,1,0,1), *E*_15_(1,1,1,0), and *E*_16_(1,1,1,1), where the stability of the mixed equilibrium point is not considered because it must not be ESS in an asymmetric evolutionary game.

According to Lyapunov Stability Theory [67], the stability of a dynamic evolution system can be judged by the eigenvalues of the Jacobian Matrix composed of the set of dynamic evolution equations. When the real parts of the eigenvalues of the Jacobian Matrix are all negative, the equilibrium has asymptotic stability; if at least one of the eigenvalues is not negative, the equilibrium is unstable, whereupon the Jacobian Matrix is expressed as *J*:

$$J=\left[\begin{array}{cccc} \frac{\partial F(x)}{\partial x} & \frac{\partial F(x)}{\partial y} & \frac{\partial F(x)}{\partial z} & \frac{\partial F(x)}{\partial w}\\ \frac{\partial F(y)}{\partial x} & \frac{\partial F(y)}{\partial y} & \frac{\partial F(y)}{\partial z} & \frac{\partial F(y)}{\partial w}\\ \frac{\partial F(z)}{\partial x} & \frac{\partial F(z)}{\partial y} & \frac{\partial F(z)}{\partial z} & \frac{\partial F(z)}{\partial w}\\ \frac{\partial F(w)}{\partial x} & \frac{\partial F(w)}{\partial y} & \frac{\partial F(w)}{\partial z} & \frac{\partial F(w)}{\partial w} \end{array}\right]$$ (25)

Bringing *E*_1_~*E*_16_ into the Jacobian Matrix, the eigenvalues of the equilibrium points are as shown in Table 4.

**Table 4:** System equilibrium point stability for four-party pure strategy solutions.

Equilibrium point	*λ*_1_	*λ*_2_	*λ*_3_	*λ*_4_	Stabilization point
*E*_1_(0,0,0,0)	0	*r*_2_ *C*_2_ − *C*_2_ − 2*NC*_*t*_ + *r*_1_ *T*_*p*_	*r*_1_ *C*_*s*_ − *q*(*c*_*p*_ + 1/2∙*S*^2^) − 2*Np*_*t*_ + *qP*_*s*_	*βL* − *L* + *r*_1_ *A*_*l*_	(0,×,×,−)
					ESS
					Scenario 1
*E*_2_(0,0,0,1)	r_1_ *A*_*l*_ − *A*_*l*_	2*Nc*_*s*_ + *r*_2_ *C*_2_ − *C*_2_ + *Rc*_*e*_ + *r*_1_ *T*_*p*_	2*Np*_*s*_ − *q*(*c*_p_ + 1/2∙*S*^2^)+*r*_1_ *C*_s_ + *qP*_*s*_	*L* − *βL* − *r*_*1*_ *A*_*l*_	(−,+,+,+)
					Unstable
*E*_3_(0,0,1,0)	*Sb*_*p*_ − *C*_*S*_ + *r*_1_ *C*_*s*_	*r*_2_ *C*_2_ − *C*_2_ − 2*Nc*_*t*_ + *r*_1_ *T*_*p*_ + *ρRc*_*p*_	2*Np*_*t*_ + *q*(*c*_*p*_ + 1/2∙*S*^2^) − *r*_1_ *C*_*s*_ − *qP*_*s*_	*βL* − *L* + *r*_1_ *A*_*l*_ + *γE* + *αS*∙*q*	(+,×,×,−)
					Unstable
*E*_4_(0,0,1,1)	*Sb*_*p*_ − *C*_*S*_ + *r*_1_ *C*_*s*_ − *A_l_* + *r*_1_ *A*_*l*_	2*Nc*_*s*_ − *C*_2_ + *r*_2_ *C*_2_ + *Rc*_*e*_ + *ρRc*_*p*_ + *r*_1_ *T*_*p*_	*q*(*c*_*p*_ + 1/2∙*S*^2^) − 2*Np*_*s*_ − *r*_1_ *C*_*s*_ − *qP*_*s*_	*L* − *βL* − *r*_1_ *A*_*l*_ − *γE* − *αS*∙*q*	(×,+,−,×)
					Unstable
*E*_5_(0,1,0,0)	*Sb*_*c*_ − *T*_*p*_ + *r*_1_ *T*_*P*_	*C*_2_*r*_2_ − *C*_2_ + 2*Nc*_*t*_ − *r*_1_ *T*_*p*_	*r*_1_ *C*_*s*_ − *q*(*c*_*p*_ + 1/2∙*S*^2^) − 2*Np*_*t*_ + *qP*_*s*_ + *ρRp*_*a*_ − *ρRp*_*n*_	*βL* − *L* + *r*_1_ *A*_*l*_ + *θE*	(+,×,×,−)
					Unstable
*E*_6_(0,1,0,1)	*Sb*_*c*_ − *A*_*l*_ + *r*_1_ *A*_*l*_ − *T*_*p*_ + *r*_1_ *T*_*P*_	*C*_2_ − *r*_2_ *C*_2_ − 2*Nc*_*s*_ − *Rc*_*e*_ − *r*_1_ *T*_*p*_	2*Np*_*s*_ − *q*(*c*_*p*_ + 1/2∙*S*^2^) + *r*_1_ *C*_*s*_ + *qP*_*s*_ + *ρRp*_*a*_ − *ρRp*_*n*_	*L* − *βL* − *θE* − *r*_1_ *A*_*l*_	(×,−,+,×)
					Unstable
*E*_7_(0,1,1,0)	*Sb*_*c*_ + *Sb*_*p*_ − *C*_*S*_ + *r*_1_ *C*_*s*_ − *T*_*p*_ + *r*_1_ *T*_*P*_	*C*_2_ − *r*_2_ *C*_2_ + 2*Nc*_*t*_ − *ρRc*_*p*_ − *r*_1_ *T*_*p*_	2*Np*_*t*_ + *q*(*c*_*p*_ + 1/2∙*S*^2^) − *r*_1_ *C*_*s*_ − *qP*_*s*_ − *ρRp*_*a*_ + *ρRp*_*n*_	*βL* − *L* + *θE* + *γE* + *r*_1_ *A*_*l*_ + *αS*∙*q*	(+,+,+,+)
					Unstable
*E*_8_(0,1,1,1)	*Sb*_*c*_ + *Sb*_*p*_ − *C*_*s*_ − *A*_*l*_ − *T*_*p*_ + *r*_1_ *A*_*l*_ + *r*_1_ *C*_s_ + *r*_1_ *T*_*P*_	*C*_2_ − *r*_2_ *C*_*2*_ − 2*Nc*_*s*_ − *Rc*_*e*_ − *ρRc*_*p*_ − *r*_1_ *T*_*p*_	*q*(*c*_*p*_ + 1/2∙*S*^2^) − 2*Np*_*s*_ − *r*_1_ *C*_*s*_ − *qP*_*s*_ − *ρRp*_*a*_ + *ρRp*_*n*_	*L* − *βL* − *θE* − *γE* − *r*_1_ *A*_*l*_ − *αS*∙*q*	(+,−,−,×)
					Unstable
*E*_9_(1,0,0,0)	0	*N*_*g*_ − *C*_2_ + *r*_2_ *C*_2_ − 2*Nc*_*t*_ + *T*_*p*_	*C*_*s*_ − 2*Np*_*t*_ − *q*(*c*_*p*_ + 1/2∙*S*^2^) + *qP*_*s*_	*βL* − *L* + *A*_*l*_	(0,×,−,−)
					ESS
					Scenario 2
*E*_10_(1,0,0,1)	*A*_*l*_ − *r*_1_ *A*_*l*_	2*Nc*_*s*_ − *C2* + *r*_2_*C*_2_ + *N*_*g*_ + *Rc*_*e*_ + *T*_*p*_	*C*_*s*_ + 2*Np*_*s*_ − *q*(*c*_*p*_ + 1/2∙*S*^2^) + *qP*_*s*_	*L* − *βL* − *A*_*l*_	(+,+,+,+)
					Unstable
*E*_11_(1,0,1,0)	*C*_s_ − *r*_1_ *C*_*s*_ − *Sb*_*p*_	*N*_*g*_ − 2*Nc*_t_ − *C*_2_ + *r*_2_*C*_2_ + *T*_*p*_ + *ρRc*_*p*_	2*Np*_*t*_ − *C*_*s*_ + *q*(*c*_*p*_ + 1/2∙*S*^2^) − *qP*_*s*_	*βL* − *L* + *A*_*l*_ + *γE* + *αS*∙*q*	(−,×,+,×)
					Unstable
*E*_12_(1,0,1,1)	*A*_*l*_ + *C*_*s*_ − *Sb*_*p*_ − *r*_1_ *A*_*l*_ − *r*_1_ *C*_*s*_	2*Nc*_*s*_ − *C*_2_ + *r*_2_ *C*_2_ + *N*_*g*_ + *Rc*_*e*_ + *T*_*p*_ + *ρRc*_*p*_	*q*(*c*_*p*_ + 1/2∙*S*^2^) − 2*Np*_*s*_ − *C*_*s*_ − *qP*_*s*_	*L* − *βL* − *A*_*l*_ − *γE* − *αS*∙*q*	(×,+,−,×)
					Unstable
*E*_13_(1,1,0,0)	*T*_*p*_ − *Sb*_*c*_ − *r*_1_ *T*_*P*_	*C*_2_ − *r*_2_ *C*_2_ + 2*Nc*_*t*_ − *N*_*g*_ − *T*_*p*_	*C*_*s*_ − 2*Np*_*t*_ − *q*(*c*_*p*_ + 1/2∙*S*^2^) + *qP*_*s*_ + *ρRp*_*a*_ − *ρRp*_*n*_	*βL* − *L* + *A*_*l*_ + *θE*	(−,×,×,−)
					ESS
					Scenario 3
*E*_14_(1,1,0,1)	*A*_*l*_ − *Sb*_*c*_ + *T*_*P*_ − *r*_1_ *A*_*l*_ − *r*_1_ *T*_*P*_	*C*_2_ − *r*_2_ *C*_*2*_ − 2*Nc*_*s*_ − *N*_*g*_ − *Rc*_*e*_ − *T*_*p*_	*C*_*s*_ + 2*Np*_*s*_ − *q*(*c*_*p*_ + 1/2∙S^2^) + *qP*_*s*_ + *ρRp*_*a*_ − *ρRp*_*n*_	*L* − *βL* − *A*_*l*_ − *θE*	(×,−,+,+)
					Unstable
*E*_15_(1,1,1,0)	*C*_*s*_ − *Sb*_*c*_ − *Sb*_*p*_ + *T*_*p*_ − *r*_1_ *C*_*s*_ − *r*_1_ *T*_*P*_	*C*_2_ − *r*_2_ *C*_2_ + 2*Nc*_*t*_ − *N*_*g*_ − *T*_*p*_ − *ρRc*_*p*_	2*Np*_*t*_ − *C*_*s*_ + *q*(*c*_*p*_ + 1/2∙S^2^) − *qP*_*s*_ − *ρRp*_*a*_ + *ρRp*_*n*_	*βL* − *L* + *A*_*l*_ + *θE* + *γE* + *αS*∙*q*	(−,×,×,+)
					Unstable
*E*_16_(1,1,1,1)	*A*_*l*_ + *C*_*s*_ + *T*_*p*_ − *Sb*_*c*_ − *Sb*_*p*_ − *r*_1_ *A*_*l*_ − r_1_ *C*_*s*_ − r_1_ *T*_*P*_	*C*_2_ − r_2_ C_2_ − 2*Nc*_*s*_ − *N*_*g*_ − *Rc*_*e*_ − *T*_*p*_ − *ρRc*_*p*_	*q*(*c*_*p*_ + 1/2∙*S*^2^) − 2*Np*_*s*_ − 2*Np*_*s*_ − *qP*_*s*_ − *ρRp*_*a*_ + *ρRp*_*n*_	*L* − *βL* − *A*_*l*_ − *θE* − *γE* − *αS*∙*q*	(×,−,−,−)
					ESS
					Scenario 4

For scenarios 1 and 2, the community is passive, the enterprises provide weak smart services, and the household with elderly members choose the traditional elderly care, which prevents the local government from gaining smart utility. (0,0,0,0) is the least desirable stabilization result under $\left(r_{2}-1\right)C_{2}+r_{1}T_{p}<2Nc_{t}$, $-\Delta C+qP_{S}+r_{1}C_{S}<2Np_{t}$ conditions. This suggests that when the local government chooses negative support, the difference between transfers received by community and the cost of community assistance, and the sum of enterprises’ net benefits from smart construction and government subsidies, both of which are lower than the value perception of double preference on households. All players are passive and sluggish and lack of better coordination, the implementation of smart community elderly care is in trouble. For scenario 2, that is, (1,0,0,0), although the local government support attitude is more positive, and the transfer payment and incentive subsidy of the local government have been increased, the relative net benefit of other subjects actively participating in the construction of smart community elderly care is still not greater than zero, or the relative net benefit is less than ideal, so that they cannot bear excessive cost risks and choose conservative strategy.

Scenario 3 is a less desirable stable outcome, where the community unilaterally proceeds to increase the smart level with positive support from the local government and traditionalized services chosen by household. The stability conditions are $\left(1-r_{2}\right)C_{2}-T_{P}-N_{g}+2Nc_{t}<0$, $-\Delta C+qP_{S}+C_{S}+\rho Rp_{n}-\rho Rp_{a}-2Np_{t}<0$. This indicates that the community’s own expenditures are less than the difference between the negative effect of the community’s inversion with the government and the value perception of double preference for households; and the net benefit of enterprise smart construction is less than the sum of the value-added benefit when the community actively assists and the perceived effect of double preference. Compared with scenario 2, in which the community chooses to actively assist because the relative net benefit is greater than zero and more desirable, the local government provides transfer payments to the community considering community behavior. Additionally, despite the community’s active and increased assistance from enterprise in getting smart elderly care, they may still choose to provide low-smart or non-smart services due to cost, efficiency, and social environment factors. The household will be stimulated by the community to some extent, but the improved health benefit of choosing smart elderly care is not apparent, so they are in a state of waiting and seeing.

Scenario 4 is (1,1,1,1), meaning (positive support, active assistance, highly smart services, choosing smart services), which is the ideal result under the condition of $-\left(r_{1}-1\right)\left(T_{P}+C_{s}+A_{L}\right)<Sb_{c}+Sb_{p}$, when all players proactive participate in the construction and service of smart community elderly care. This shows that if government subsidies are adequate and supply side capacity and preparation for smart is sufficient, households with elderly members are more likely to choose smart services to enjoy a more convenient and modern elderly life. Meanwhile, the household will also play a role in influencing community and enterprise to promote smart community elderly care system. Such a stable strategy facilitates the achievement of smart elderly care goals, provides a favorable condition for building smart modern city, and creates the highest social surplus.

## 5. Evolutionary path and strategy combination analysis and simulation

### 5.1 Initial setup of system simulation

System dynamics as the powerful tool to analyse the relationship between the feedback structure of the variables within the modern social and economic system, focusing on revealing the pattern of change of things and provide evolutionary trends in decision-making thinking, and with the combination of evolutionary game theory has been extensively used in a variety of fields [68]. In order to intuitively reveal the local government, community, service supply enterprise and household with elderly members initial evolutionary path and important parameter sensitivity, we further visualise the behaviour of the four parties by means of numerical simulation of system dynamics. Based on Vensim PLE, set the simulation start time as 0, simulation end time as 100, and simulation step size as 0.001. Considering the current situation of smart community elderly care in China, we selected the regional data published by the Anhui provincial government report in 2022 as the main basis [69], and we set the simulation parameters considering market research and the parameter settings in the literature [60, 70, 71]. After data processing, Table 5 was obtained.

**Table 5:** Parameter assignment.

*T*_*p*_	*C*_*s*_	*A*_*l*_	*r*_1_	*Sb*_*c*_	*Sb*_*p*_	*C*_2_	*r*_2_	*Rc*_*p*_	*Rc*_*e*_	*Nc*_*s*_	*Nc*_*t*_	*N*_*g*_	*ρ*
0.6	1.1	3.6	0.6	1.3	1.9	3.5	0.6	1.5	2.5	2.7	1.7	4	0.8
*c*_*p*_	*S*	*P*_*s*_	*q*	*Rp*_*a*_	*Rp*_*n*_	*Np*_*s*_	*Np*_*t*_	*α*	*β*	*θ*	*γ*	*E*	*L*
5.5	1.7	9.3	0.8	2	0.8	5.7	3.8	0.5	0.75	0.1	0.1	70	60

**Fig 7:**
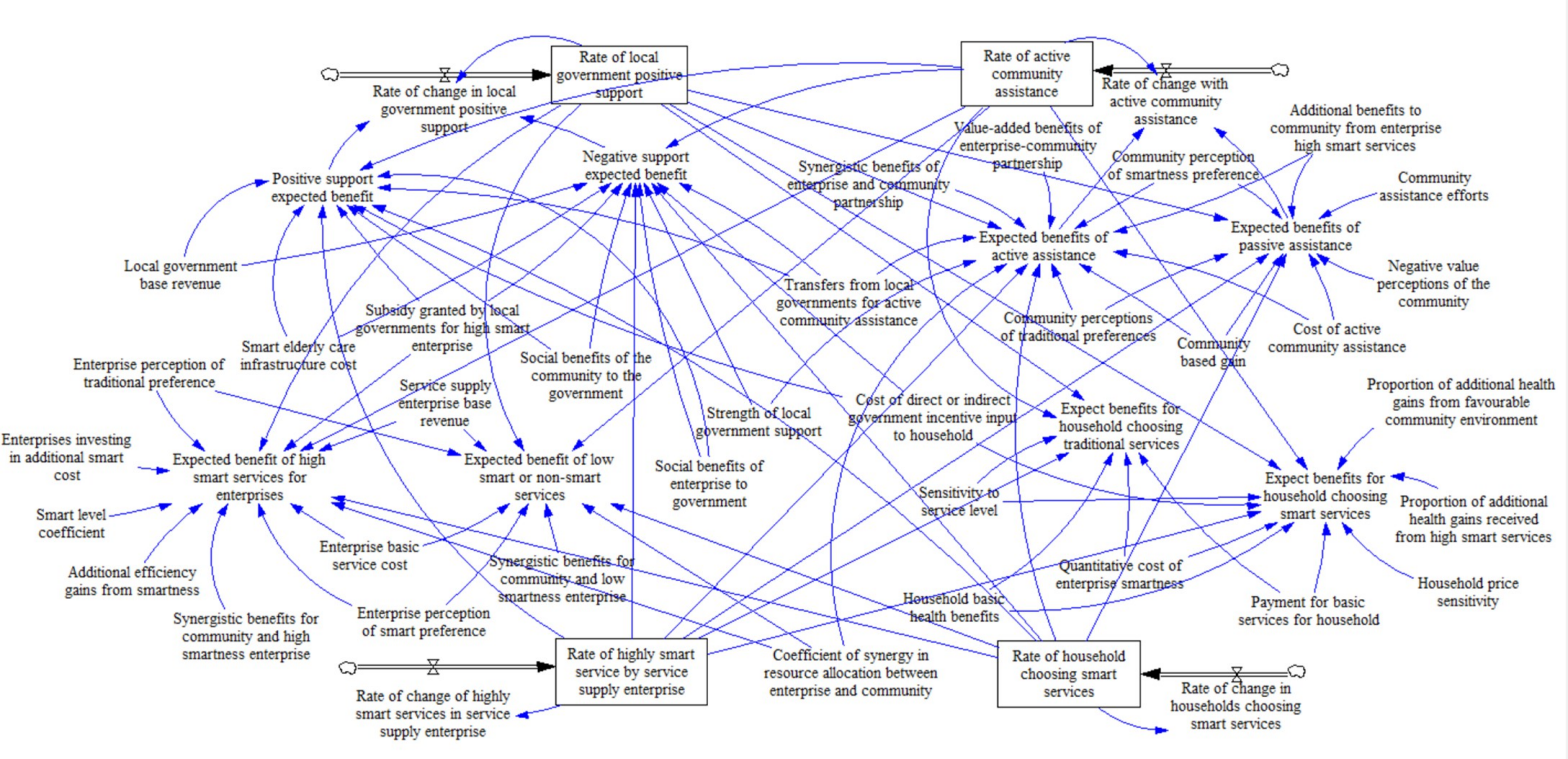
SD-based game model of multi-subject behaviour in smart community elderly care.

After the initial setup of the model, the flow, stock, and auxiliary variables present in the model are considered in order to further construct the flow diagram. Based on the equation of the relationship between the variables, four variables, the rate of active support from local government, active assistance from community, highly smart services from enterprise, and household choosing smart services, are set as stocks. Four parameters, including the rate of change of positive support from government, active assistance from the community, highly smart services from enterprise, and selection of smart services by household, were set as flows. In addition, 39 variables were used as auxiliary variables. The logical relationship between flows and stocks is determined by the set of replicated dynamic equations (*fx*,*fy*,*fz*,*fw*), and the relationship between auxiliary variables and flows is jointly determined by the expected benefit of the quadrilateral subjects and the replicated dynamic equations, resulting in the SD model shown in Fig 7.

**Fig 8:**
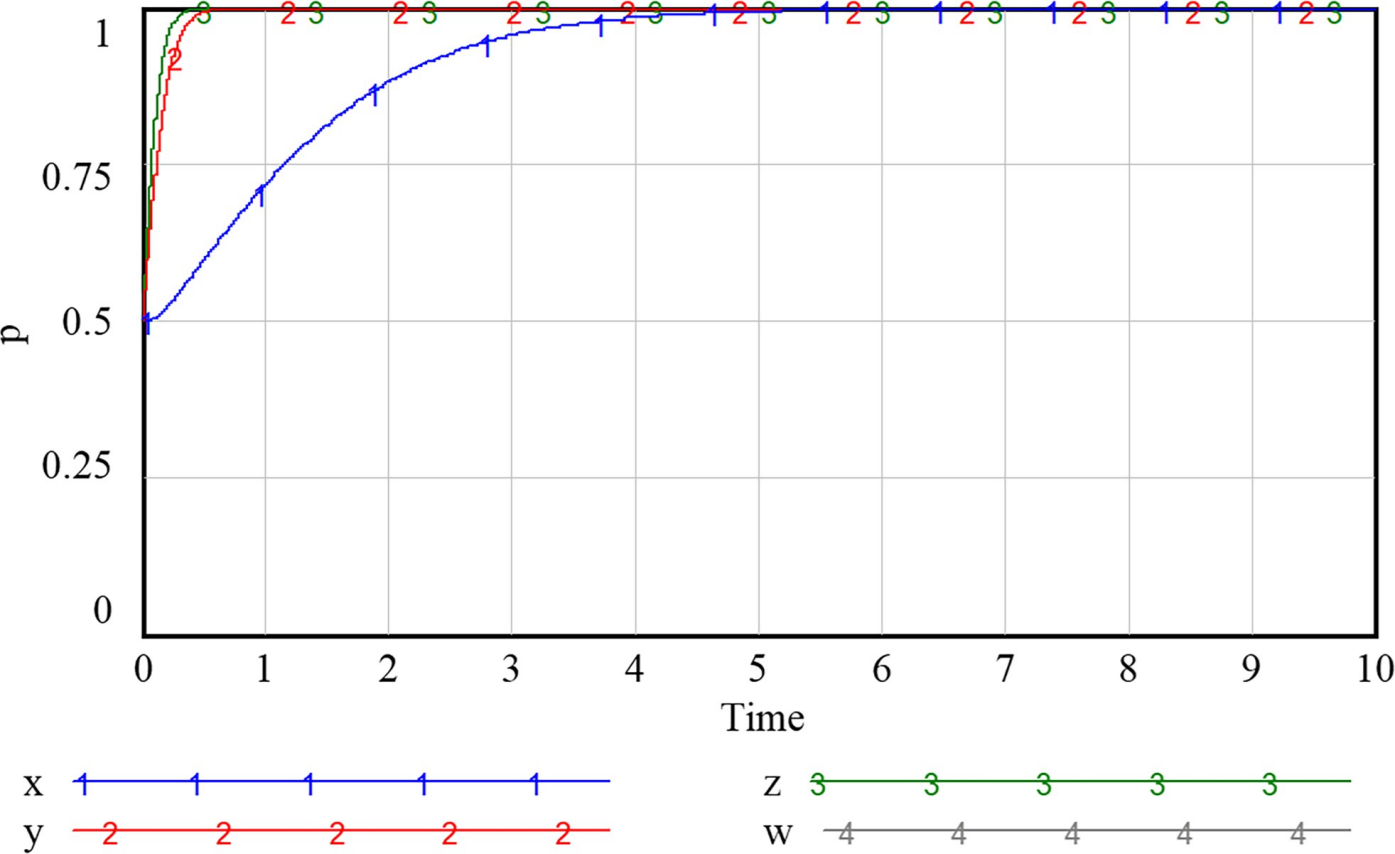
Strategy evolution process of each party under initial assignment.

At the same time, assuming that the probability of government, community, enterprise and household strategy taking value are all 0.5, it is not difficult to see that under the initial assignment and when all four parties adopt mixed strategy, the system exists pure strategy evolution equilibrium point (1,1,1,1). The results verify the reasonableness and stability of the evolution model, and the evolution process is shown in Fig 8.

### 5.2 Impact of government support on system evolution

**Fig 9:**
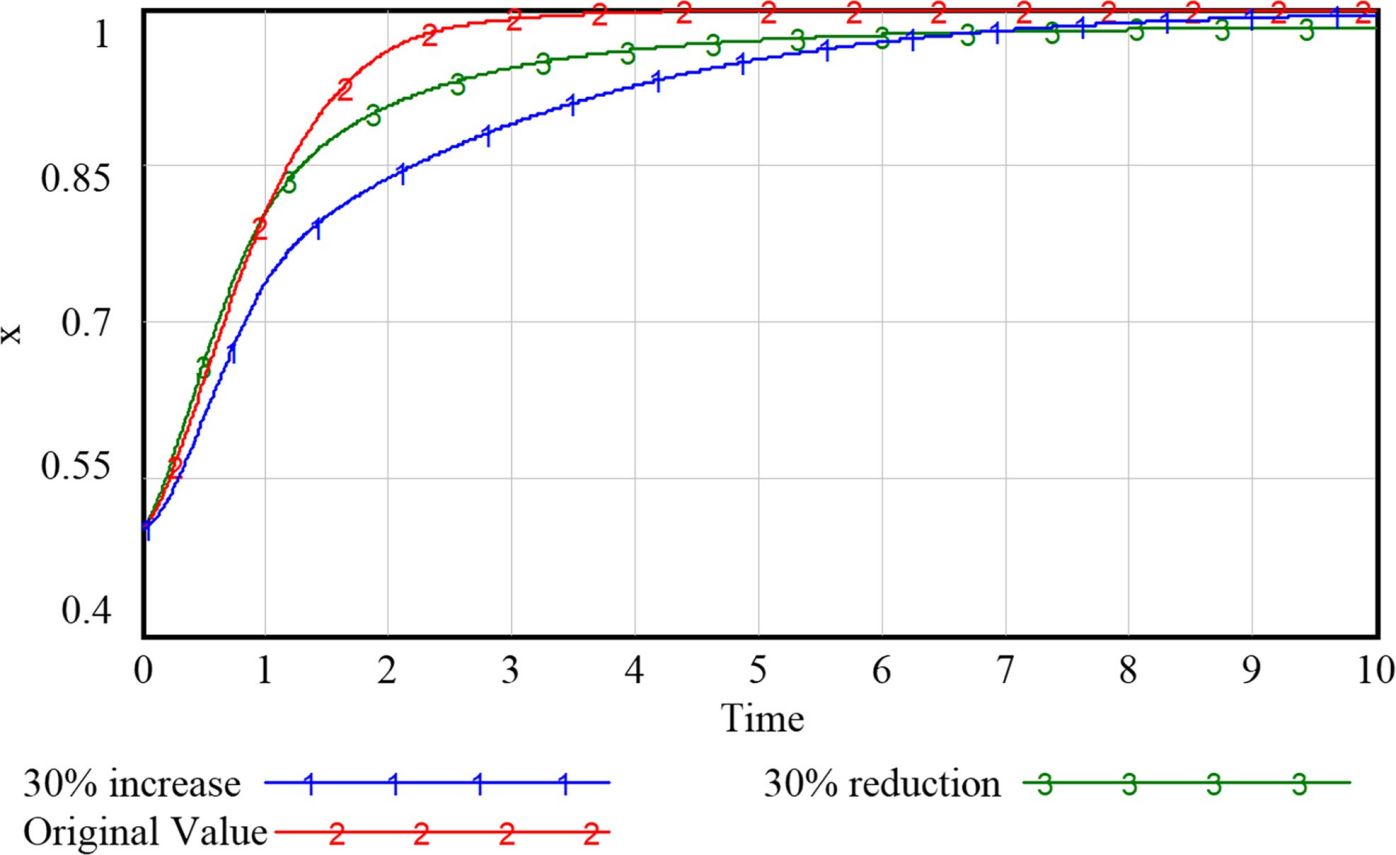
Evolutionary paths of government with different levels of support.

**Fig 10:**
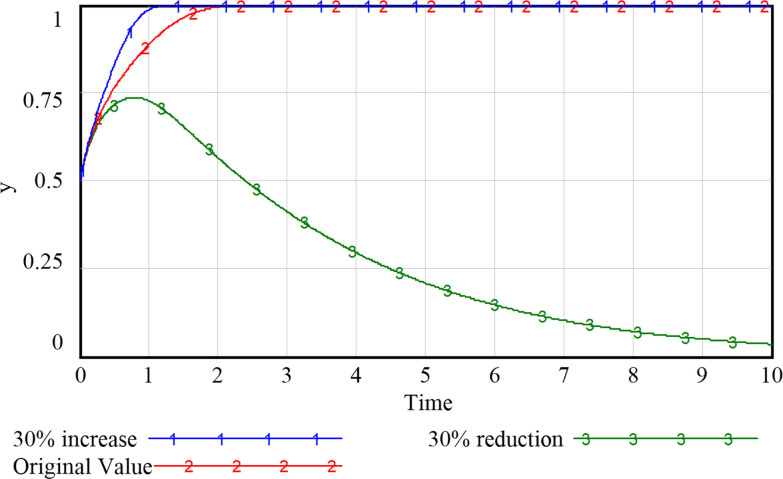
Evolutionary paths of communities with different levels of support.

**Fig 11:**
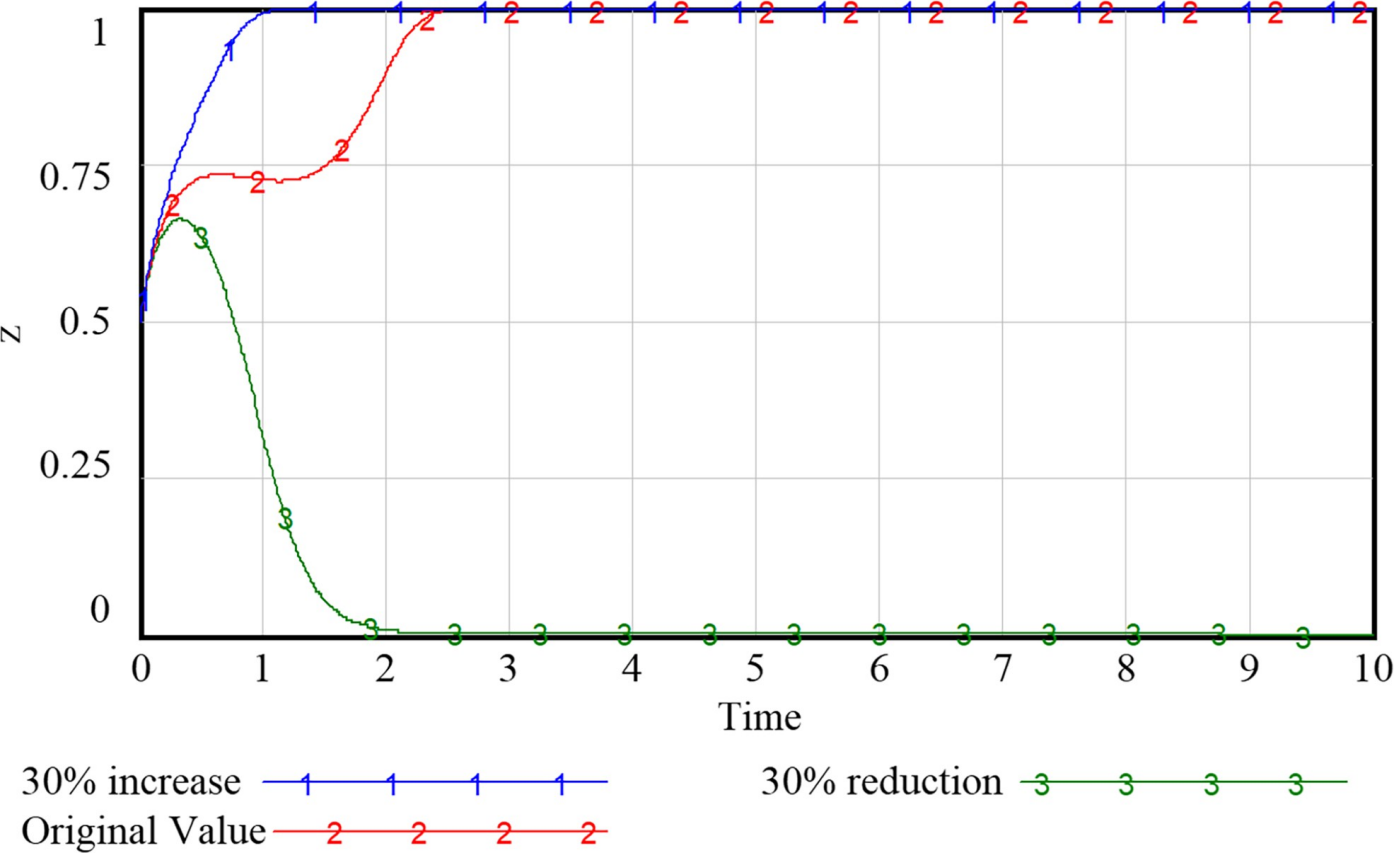
Evolutionary paths of enterprises with different levels of support.

**Fig 12:**
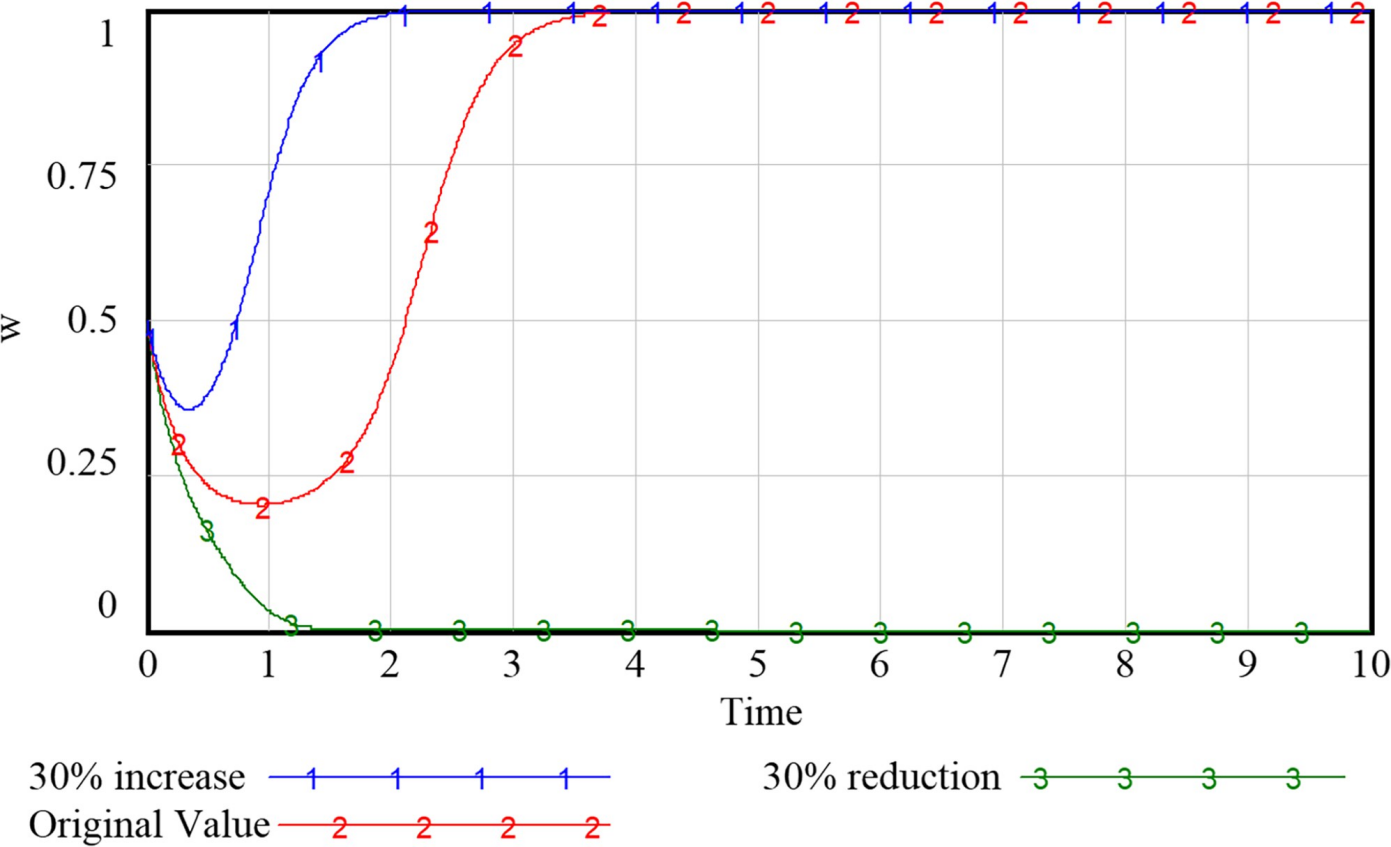
Evolutionary paths of households with different levels of support.

As can be seen from Figs 9–12, the increase in government support has a positive incentive effect on the remaining subjects. By investing financial fund, the government is able to fully stimulate the market vitality of smart community elderly care service and release the consumption potential. Regarding the government’s subsidy strategy (Fig 9), appropriate changes will not shift the government’s positive attitude, but the intention to subsidize continues to decline as the amount of the subsidy increases due to the government’s expectation to obtain the highest return for the least investment. We speculate that if unlimited subsidies are provided without certain social return, the government may eventually choose to abandon the subsidy, and the development of smart elderly services will stagnate. For the community and the enterprise (Figs 10 and 11), despite the positive development of the smart elderly care market, due to the long payback cycle of the industry itself and the difficulty of avoiding additional cost and risk in smart construction, the increased government subsidy and incentive relieve the pressure on their capital flow in time, incentivizing the community and enterprise to provide quality services. For the household with elderly members (Fig 12), with increased government subsidy and the readiness of the community and enterprise to proactively provide smart elderly services, the satisfaction of elderly group increases with these better services and they are more inclined to purchase smart services for higher health benefit. Therefore, it is suitable for the government to appropriately enhance the subsidies to the other three parties, so that the interests of the subjects in the system can be satisfied to varying degrees.

Further, do differences in government incentive recipients have different impacts on the final benefits? To this end, based on the evolutionary results of government support in Figs 9–12, by adjusting the incentive allocation ratio $\omega =\frac{A_{L}}{A_{L}+C_{S}+T_{P}}$ in order to explore the influence of heterogeneity in government subsidy, the numerical results are shown in Table 6. Table 6 shows that when the government’s subsidy allocation ratio is at $\omega =\frac{1}{4}$, which means that the subsidy received by the elderly household accounts for $\frac{1}{4}$ of the total subsidy of the community, service supply enterprise and household with elderly members, all three of them evolve towards low- or non-smart community elderly care. When the allocation of government subsidy is at $\omega =\frac{1}{2}$, it is obvious that an increase in the allocation of household subsidy makes the system values converge more slowly to the negative strategy. If the subsidy percentage is further raised, all three will rapidly converge on smart elderly care behaviours, with a significant positive incentive effect. Therefore, it can be obtained that the heterogeneity of subsidy not only affects the convergence rate of the system, but also directly affects the convergence state when the proportion of subsidy allocated to the household is uneven. At the same time, with a lower allocation ratio for the household, it will hinder the promotion of smart elderly care, which is caused by the fact that the starting point of smart community elderly care still belongs to the elderly eventually, and it also confirms the effectiveness of the demand-side incentive in the subsidy for elderly care services. However, excessive allocation will not be conducive to the government’s own performance of its duty, so the amount of subsidy should be reasonably allocated, so as to motivate the whole system to advance towards smart elderly services.

**Table 6:** The effect of government subsidy heterogeneity on evolution of parties’ strategies.

t	$\omega =\frac{1}{4}$				$\omega =\frac{1}{2}$				$\omega =\frac{3}{4}$			
	*x*	*y*	*z*	*w*	*x*	*y*	*z*	*w*	*x*	*y*	*z*	*w*
0	0.5	0.5	0.5	0.5	0.5	0.5	0.5	0.5	0.5	0.5	0.5	0.5
1	0.7694	0.5710	0.1445	0.0013	0.7893	0.7160	0.2973	0.0156	0.5527	0.999	1	0.9606
2	0.8488	0.3787	0.0030	0	0.8886	0.5961	0.0104	0	0.4771	1	1	0.997
3	0.8837	0.2553	0.00005	0	0.932	0.4989	0.00021	0	0.3911	1	1	1
4	0.904	0.1744	0	0	0.9561	0.4346	0	0	0.3145	1	1	1

### 5.3 Impact of perceived value of household preferences on system evolution

**Fig 13:**
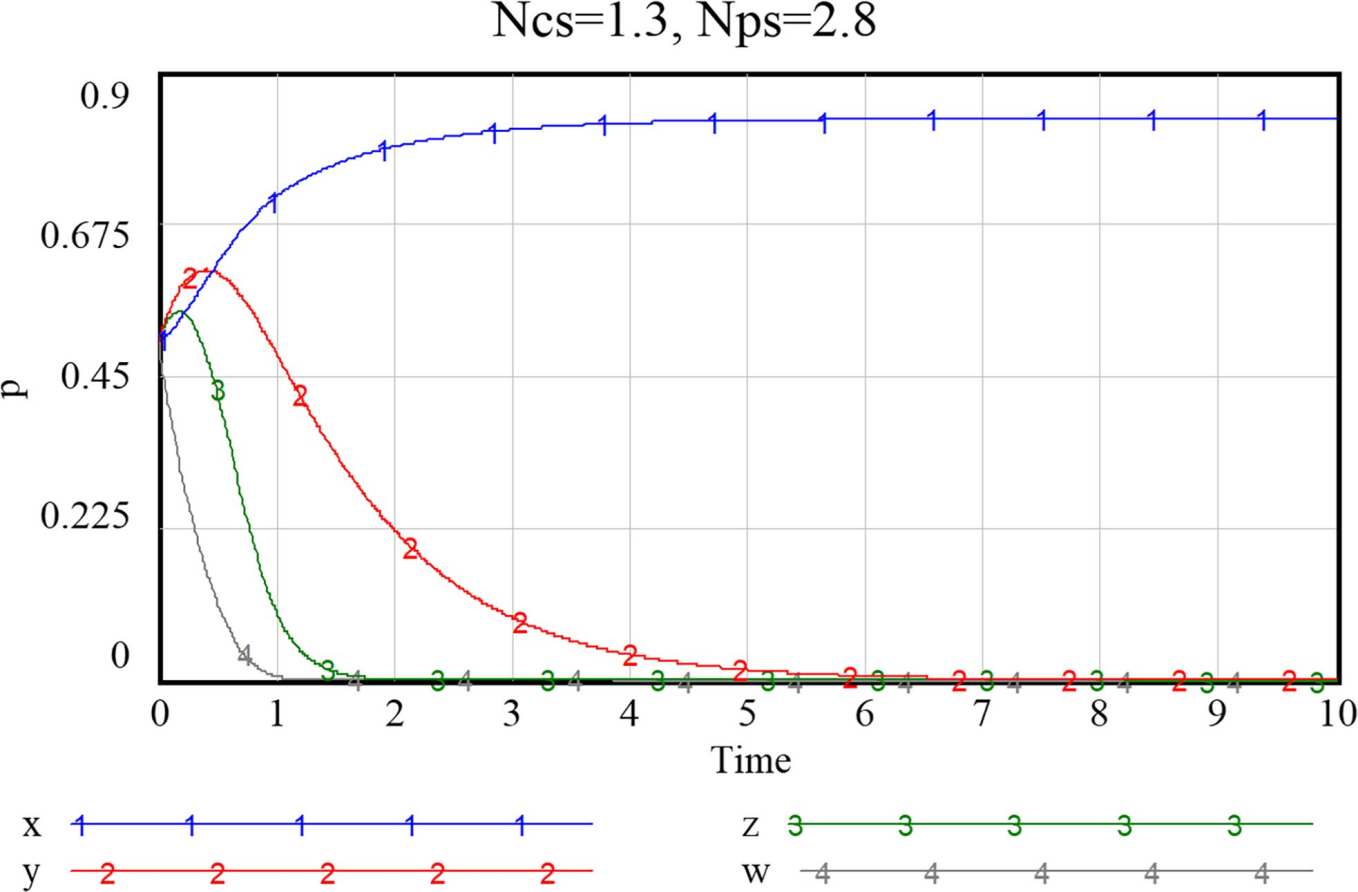
Evolutionary paths when both community and enterprise perceived value of smart preference is low.

**Fig 14:**
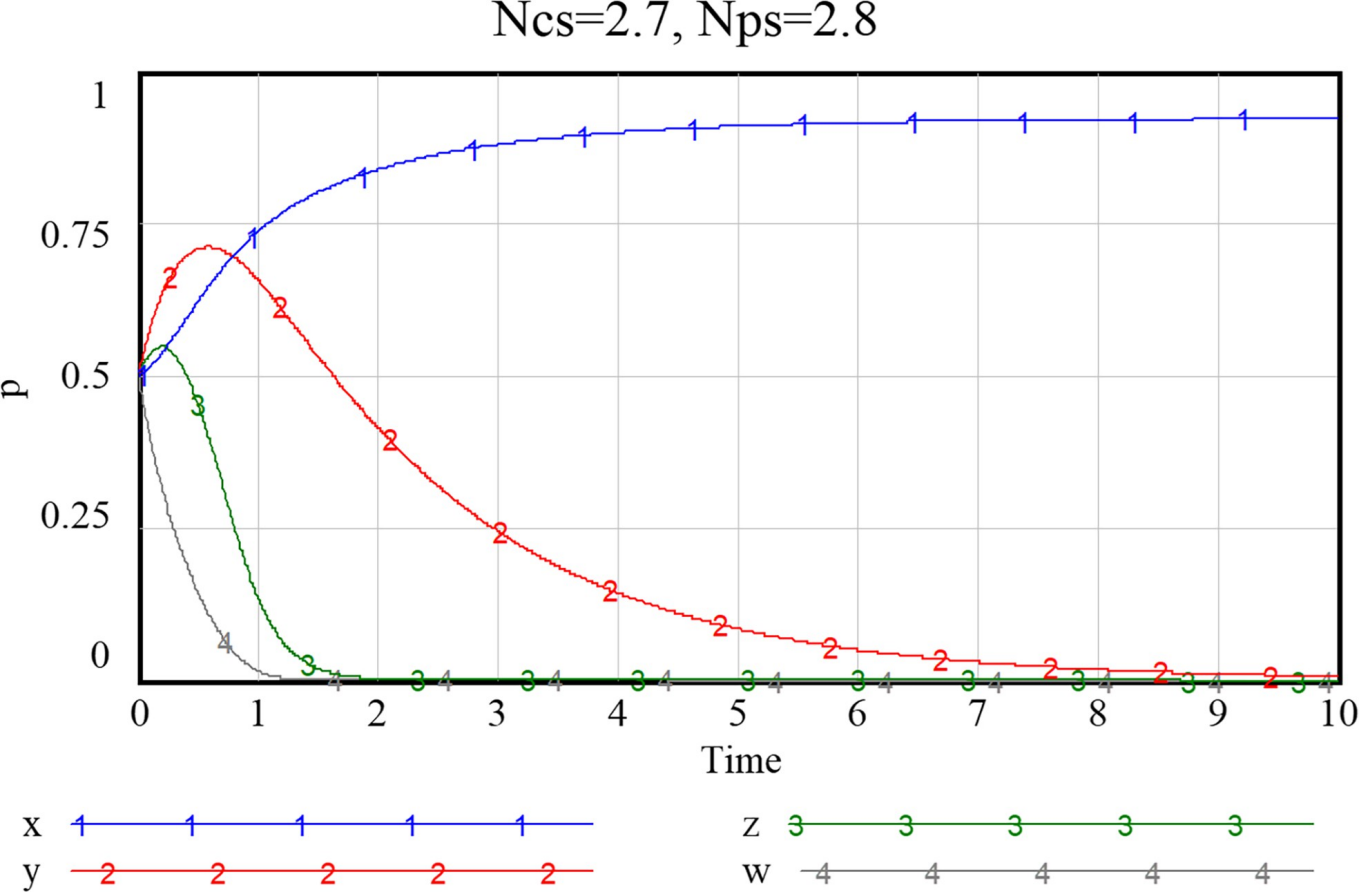
Evolutionary paths as the perceived value of community to smart preference increase.

**Fig 15:**
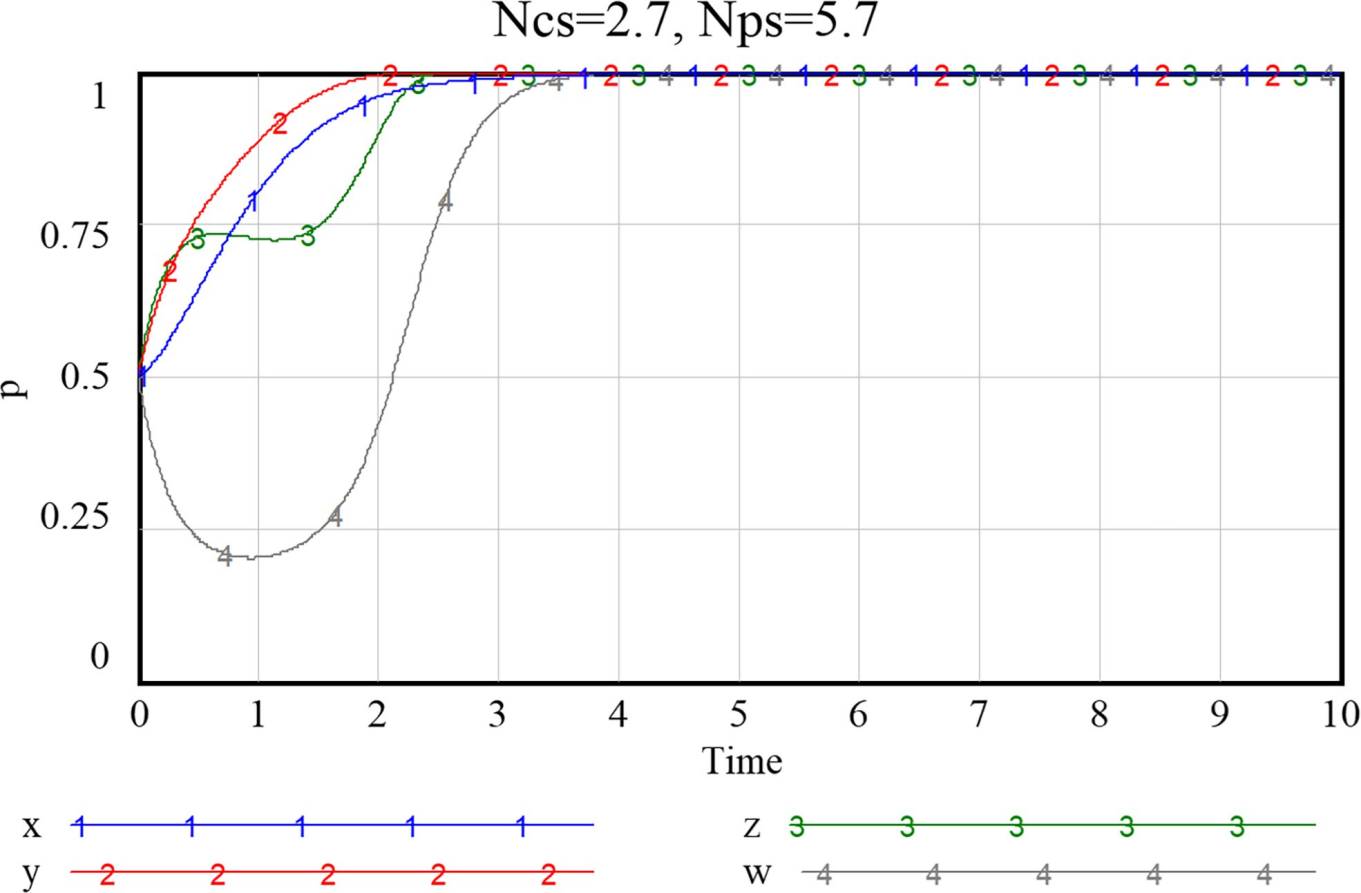
When both community and enterprise perceived value of smart preference is high.

Given the three combinations of smart service value perception scenarios, as shown in Figs 13–15, namely {*Ncs*, *Nps*} = {{1.3,2.8},{2.7,2.8},{2.7,5.7}}, the system will stabilize at a satisfactory level so that the smart transition of elderly services can be properly handled. The results showed that higher preference perception has a significant effect on the evolutionary path of all four subjects.

**Fig 16:**
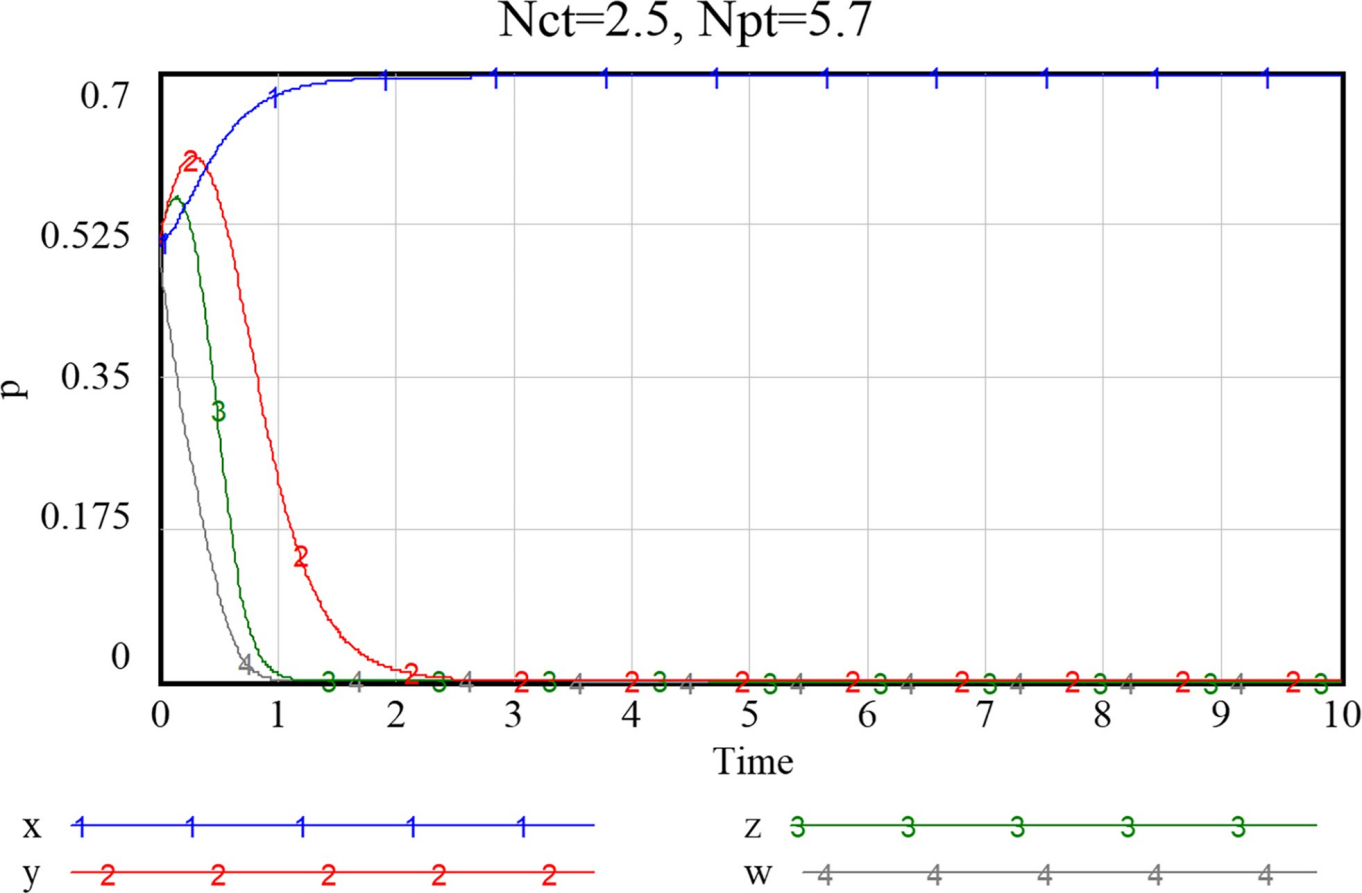
When both community and enterprise perceived value of traditional preference is high.

When the perceived value of smart services is low for both community and the service supply enterprise, the community does not adopt an active assistance strategy, and the service supply enterprise only choose to provide more traditional senior-care services. The probability of both choosing a negative smart construction strategy converges fast, implying that both parties are more decisive in choosing a conservative strategy. Additionally, the community, in the case of increased value perception of smart elderly services, although the strategy at this point still evolving towards zero, they also increase their assistance to some extent, prolonged time required for evolution toward non-smart strategies. When the value perception of both community and enterprise regarding elderly smart services rise, both of them proactively choose the smart strategy, and the probability that the government as well as households choose an active strategy also increases to a certain extent. Conversely, as Fig 16 shows, if the perceived value of traditional preference by community and enterprise increases, evolutionary stabilization strategies will proceed in the opposite direction. This implies that the impact of household preference value perceptions on community and enterprise is important, and that the higher the perceived value of the community’s or enterprise’s smartness preference, the more it will facilitate its smart service provision. Since the willingness of the elderly to participate in smart elderly care services affects the perceived value of communities and enterprises to a great extent. Therefore, from the perspective of the elderly group, by adopting strategies to promote consumption, such as effective and credible marketing, a favorable consumption environment, and direct or indirect senior care subsidies as mentioned above, can also in turn prompt communities and service supply enterprises to choose highly smart strategies.

### 5.4 Impact of external and internal parameters on system evolution

**Fig 17:**
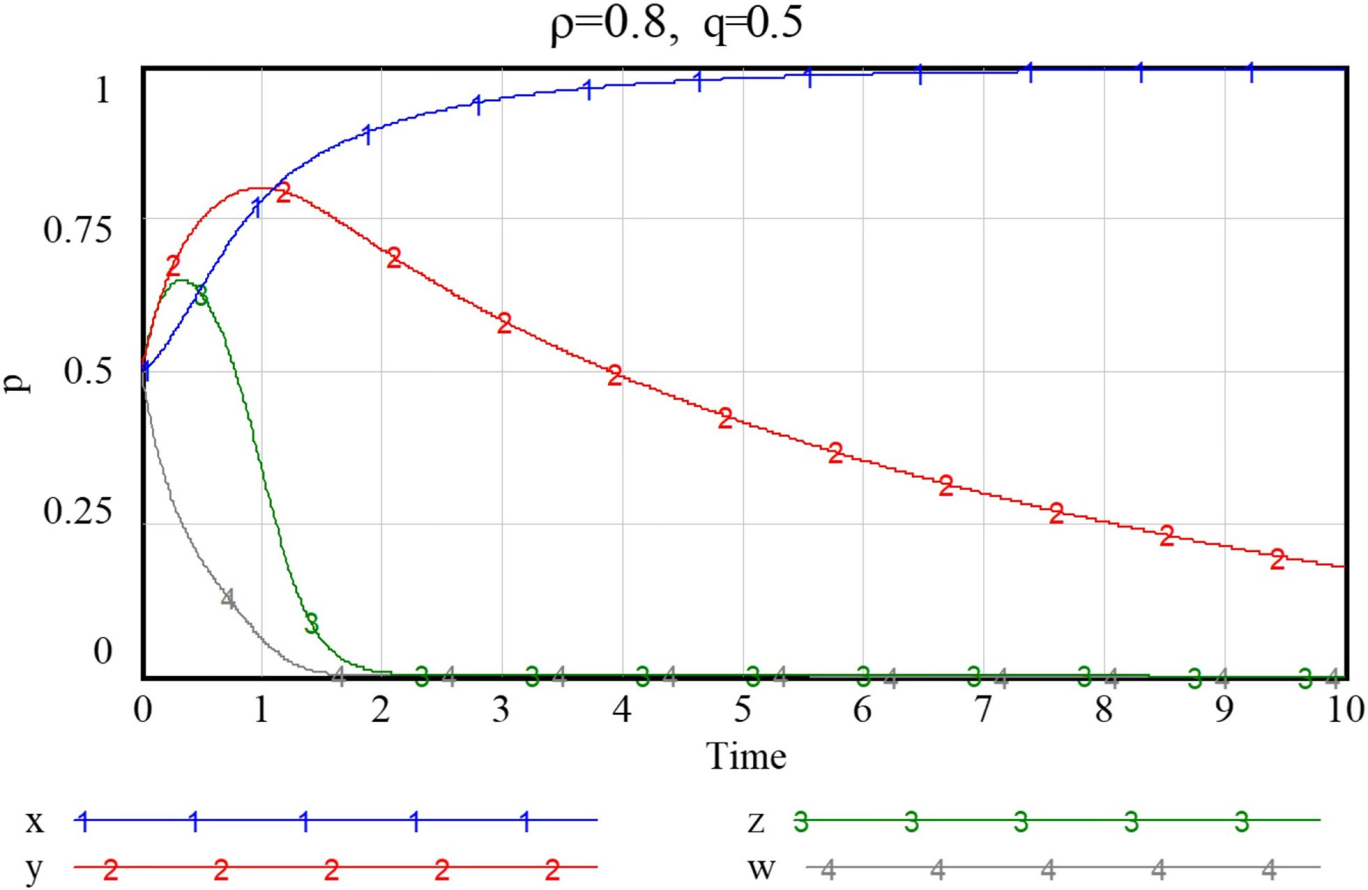
Evolutionary paths of the system at a higher resource synergy between communities and enterprises.

**Fig 18:**
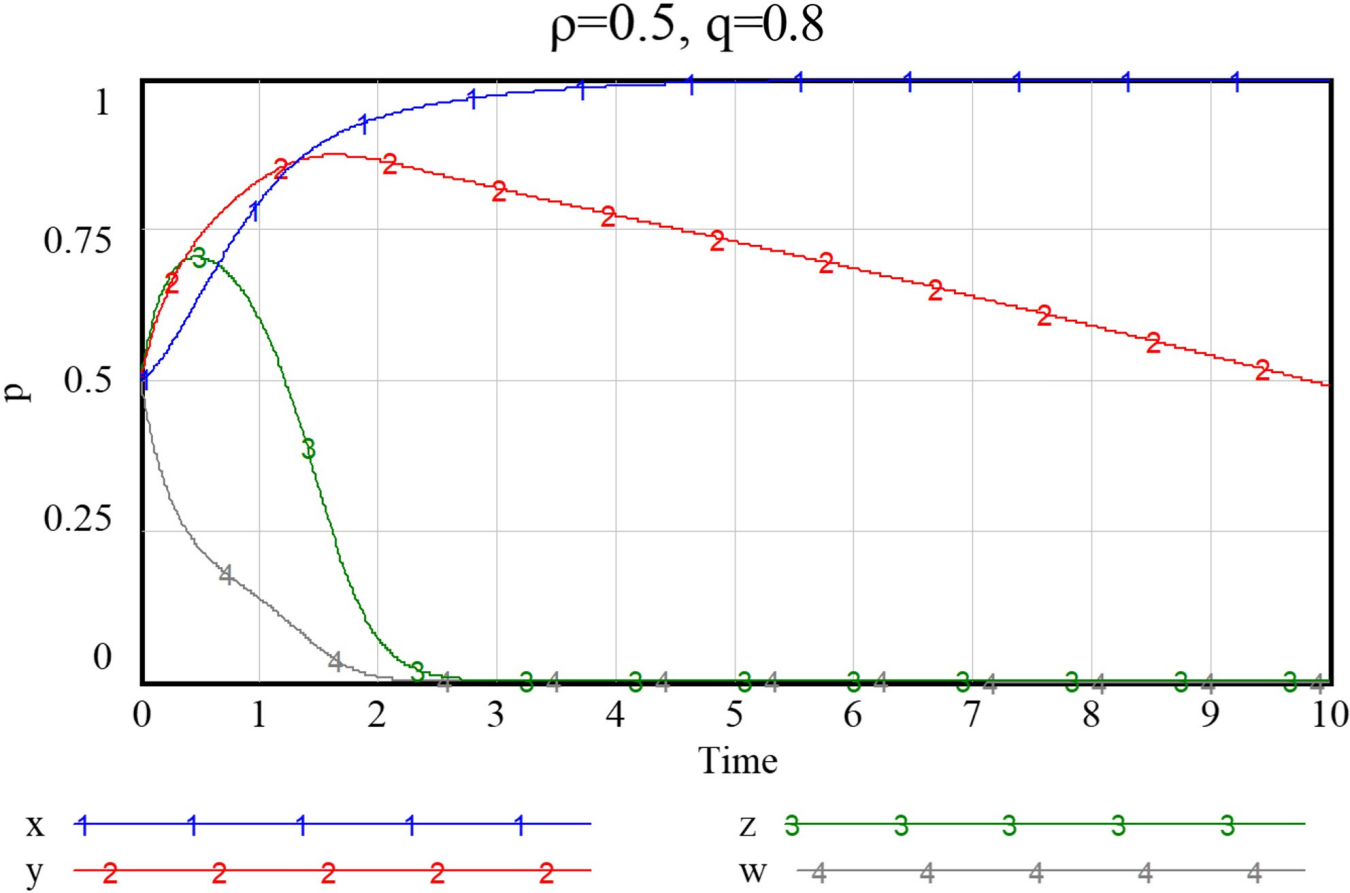
Evolutionary paths of the system at a higher level of enterprise smart.

**Fig 19:**
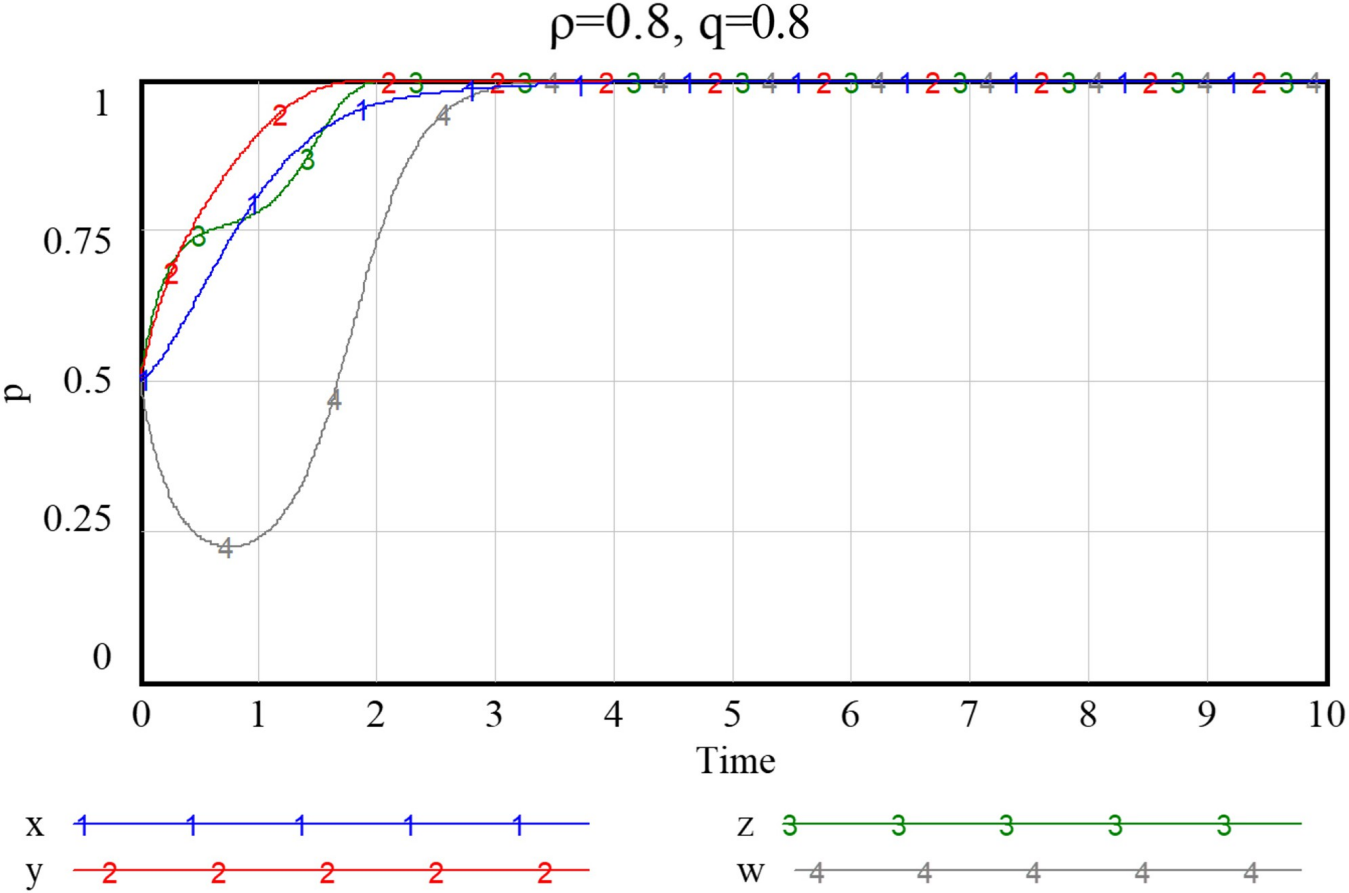
System evolution with high levels of smart and resource synergy.

Figs 17–19 shows the simulation results of the smart level of the enterprise *q* for the main endogenous dynamic factor, and the resource synergy *ρ* for the main exogenous dynamic factor on the evolution of the strategy among the four participants. Fig 17 (*ρ* = 0.8, *q* = 0.5) and Fig 18 (*ρ* = 0.5, *q* = 0.8) show that the final stabilization of the strategy are unsatisfactory for both the lower internal and external dynamic factors. When both *q* and *ρ* are higher (Fig 19), the convergence of each party becomes faster and eventually stabilizes at (1,1,1,1). This implies that increases in smart level of the enterprise and resource synergy values create not only more benefits than risks for the enterprise in the creation of smart services and more mature interaction between the community and enterprise, but also appropriate incentives to the household to stimulate their participation in smart services for the elderly. It puts a higher standard on the close collaboration between enterprise and community. As the main service provider in the smart community elderly service, it is obviously insufficient for communities or enterprises to be unilaterally active. At this point, a buffer exists in the convergence of the household with elderly members because, during the initial selection, the households are skeptical of the new elderly care model and face unknown risks, but as the tendency of other subjects to choose "positive" strategy grows, the evolutionary stabilization strategy of the household eventually converges on the smart services. The simulation results also showed that the system is more sensitive to the level of smart than the level of resource synergy since the service supply enterprise interfaces with the end household demand through face-to-face approach, which is a closer connection than between the community and the enterprise.

## 6 Discussion

With the accelerated aging of the population, high-quality elderly care services urgently need to be established to improve the social service system. As such, studies of the conversion path toward the new model of smart community elderly care are crucial to the scientific and rational construction of elderly care services. Several elderly care phenomena and management recommendations based on the findings of this study seem to be clear and straightforward, thus further energizing market-led smart elderly care in the community.

We clarified the role of the decision-making mechanism of the four participants in smart community elderly care under market domination, finding an evolutionarily stable equilibrium strategy regarding the behavioural choices of the game participants, that is, {positive support, active assistance, providing smart services, choosing smart elderly care}. The participation willingness of all gaming subjects interacts with each other, and multi-subject synergy has been a major trend in the development of the field of smart elderly service [72]. This puts forward higher requirements for the formulation of the interaction mechanism of the main bodies. Therefore, collaborative governance should be achieved by strengthening top-level design, based on the main function of different subjects on the smart community elderly care service industry and their role positioning, dynamically integrating the links between each subject and its business, eliminating as much as possible the information barriers between the policy makers, service practitioners and the end-consumers, and establishing a trust mechanism for mutual collaboration. So as to enable the government, the market and the social mechanism to make concerted efforts in smart community elderly care services, and to realise the collaboration of supply-side of the smart community elderly care service, the participation of the demand-side as well as the synergy between the supply and the demand, thereby jointly committing themselves to promote the rapid and sustainable development of smart community elderly care model.

The local government, as the dominant player in urban administration and social security, play a substantial role in promoting the construction of smart elderly care services by appropriately increasing policy and financial support. Here, household are the most sensitive to increases in subsidy, followed by enterprise and community, which confirms the effectiveness of demand-side incentives. In practice, China’s current subsidies for senior care services take into account both the supply side and the demand side, and there is an imbalance between the "weak demand and strong supply", but meanwhile, the transition of the supply structure from primarily supply-side to primarily demand-side is becoming more and more pronounced [73]. Direct or indirect subsidy to the elderly can enhance their purchasing power, thus expanding the market demand for elderly care services, and subsidy to service supply enterprise can alleviate the crowding-out effect of fiscal investment policies on private enterprise [74], which has a similar effect on the smart community elderly care industry. Therefore, the local government should actively lead all parties involved to promote the construction of smart community elderly care and the transition to smart, and improve the system and operation standards of smart community elderly care. At the same time, governments should flexibly and reasonably control the capital incentive cost input, further clarify the positioning of financial subsidy, strengthen the adjustment of the structure of financial expenditure at the supply-side level on senior care services, enhance service subsidy system for the accurate construction at the demand-side level, and appropriately promote the demand-side subsidy on the basis of the combination of supply and demand, so as to better incentivise the smart community elderly care market to improve quality and increase speed.

Communities’ and enterprises’ perceived value of household preferences facilitates their choice of strategy. When the perceived value reaches a certain threshold, communities and enterprises will actively adopt smart solutions and technologies, and the degree of acceptance and recognition of smart services by elderly households is an important factor affecting the perceived value of enterprises and communities. As consumers of elderly care services, the role of consumerist characteristics of households (including personal perception, characteristic and awareness) and external environmental factors (such as government policy, service level, and changes in the social environment) induces the selection of household preferences [75, 76]. In the meantime, under the digital technology driven smart community elderly care model, the two-way empowerment of information technology on the service supplying body and the service receiving body has prompted the transformation from "supply-oriented" to "demand-oriented", and the elderly are no longer the "passive recipients" of the services but the "active creators" of the services, which highlights the importance of the elderly’s role in the smart community elderly care industry. Hence, it is all the more necessary for policymakers to shape the consumer environment for smart community elderly care, strengthen the education of digital literacy for the elderly, popularise the concept of smart elderly care, promote the research and development of ageing-friendly technology and ageing-friendly targeted service, and thus increase the smart preference of households with elderly members. Moreover, the government should collaborate with enterprises, communities and even social organizations to make use of new technology such as big data to fully explore the actual needs of elderly groups and open up various feedback channels for the household, so as to improve the appropriateness between the needs and the groups under the smart elderly care.

Finally, the two key factors of enterprise smart level and enterprise-community resource synergy together promote the smart transition and construction of the supply side, systems are more sensitive to the level of smart than resource synergy, and enterprise and community at the same time effective implementation is more conducive to the realization of the smart community elderly care goal. Internally, as the most important service providers, enterprises should follow the trend and fully grasp the opportunity, utilize digital and information technology to build a smart elderly service platform, and emphasize the learning and application of new technologies as well as the construction of talent teams. Meanwhile, despite the fact that service supply enterprises have a certain foundation in carrying out smart elderly care services, some of them also have problems such as shortage of resources and lack of specialization, and are bound to face some challenges in penetrating into the field of smart elderly care services. Therefore, in practice, enterprises need to advance from shallow to deep according to their own situation and objective environment, so as to realize the transition from low-threshold services to specialized services, and from standard services to smart customized services. On this basis, enterprises should further improve the age-appropriateness of intelligent products and services, pay attention to the experiences of elderly people with smart services, and overcome the experience of coldness and rationality of digitalization and intelligence by providing an inclusive and friendly service environment. Outwardly, when introducing service supply enterprises, communities are expected to stipulate access thresholds, industry standards and other preliminary preparations in advance, in order to enhance the fitness of communities and enterprises as much as possible, and lay the foundation for subsequent cooperation. Communities should also establish a sound monitoring and feedback mechanism, and make full use of digital technology to achieve integrated information decision making, so as to reduce the problems of low synergy in the resource system and low utilization of resources. Then, under the government’s overall planning, it actively coordinates smart community elderly care services, and facilitate the system’s transition to smart elderly care.

## 7 Conclusion

Smart community elderly care is important for the innovative development of the senior-care industry ensure the growing demand for senior-care services is met. In this study, we constructed an evolutionary game model that includes the local government, community, service supply enterprise, and household with elderly members, then applied system dynamics to explore the pathways for the smart community elderly care system to reach an ideal equilibrium state during the smart transition process. This study demonstrates the positive effect of government stimulation on the smart community elderly care and the evolutionary impact of the main internal and external factors that energise the market, as well as confirming the strategic choices made by household preferences for the community and enterprise. These findings provide new insights into the evolutionary toward smart services in aging society, which is conducive to promoting the scale and commercial development of China’s smart community elderly care industry, and further provide references for the formulation of development incentive mechanism.

However, this study has some limitations. For example, although smart community elderly care has received certain attention, it may produce different effects and mechanisms between different regions due to factors such as regional planning, level of economic development, population characteristic, and customary and cultural difference. Future studies can further elaborate the construction of smart community elderly care in each geographic region and provide more targeted management recommendations. Secondly, smart technology that are key element in smart community elderly care, such as the smart elderly care integrated platform, have not yet been analysed in depth in this paper. In future research, smart technology can be included in the decision-making model to further emphasize its important impact in the smart community elderly care system.

## Supporting information

S1 Checklist Human participants research checklist. (DOCX)

S1 Fig (ZIP)

## References

[1] Liu XY, Chau KY, Liu XX, Wan Y. The progress of smart elderly care research: A scient metric analysis based on CNKI and WOS. International Journal of Environmental Research and Public Health. 2023; 20:1086. 10.3390/ijerph20021086. 36673842 PMC9859223

[2] Lu JH, Lin JK. Demographic feature of a severe aging society, risk identification and strategic response. Studies on Socialism with Chinese Characteristics. 2023; 169(01):59–68.

[3] Wang C, Li DY, Zhou YP. The supply of community-based smart elderly care services: responsibility network, realistic constraints and mechanism construction. Population and Economy. 2023; 256(01):120–138.

[4] Wang QX, Liu J, Zheng Y. Evolutionary game analysis of community elderly care service regulation in the context of "Internet +". Frontiers in Public Health. 2022; 10:1093451. doi: 10.3389/fpubh.2022.1093451 36620239 PMC9815532

[5] Willemse E, Anthierens S, Farfan-Portet MI, Schmitz O, Macq J, Bastiaens H, et al. Do informal caregivers for elderly in the community use support measures? A qualitative study in five European countries. BMC Health Services Research. 2016; 16:1–10. doi: 10.1186/s12913-016-1487-2 27423182 PMC4947246

[6] Yang LC, Shi WK. Reform of community-based home care services in China: practice, challenges and recommendations. Sub National Fiscal Research. 2022; 09:84–91.

[7] Huang J. Integration and empowerment: A study on the optimization of community embedded senior care service model. Academia. 2022; 288(05):151–160. doi: 10.3969/j.issn.1002-1698.2022.05.014

[8] Liang CY, Hong WJ, Ma YM. Comprehensive elderly care: A new model for the development of smart elderly care in the new era. Journal of Beijing University of Technology (Social Science Edition). 2022; 24(06):116–124. doi: 10.15918/j.jbitss1009-3370.2022.6126

[9] Courtney KL, Demiris G, Rantz M, Skubic M. Needing smart home technologies: The perspectives of older adults in continuing care retirement communities. Informatics in Primary Care. 2008; 16(3):195–201. doi: 10.14236/jhi.v16i3.694 19094406

[10] Cherie BN, Sajda SQ. Adaptations of electronic health records to activate physicians’ knowledge: How can patient centered care be improved through technology? Health Technol. 2014; 4:59–73. doi: 10.1007/s12553-013-0072-5

[11] Schnell MW. The wisdom of the elderly. Z Gerontol Geriatr. 2010; 43:393. doi: 10.1007/s00391-010-0119-4 21125373

[12] Hou HP, Wei H, Wang YC, Yu J, Qiu GX. Research on the construction path of China’s elderly smart health care platform. Strategic Study of CAE. 2022; 24(02):170–178.

[13] Sun Q, Hao G, Ding YY. Digital platform drives supply structure optimization of smart elderly care services: game analysis based on the perspective of value co-creation theory commercial research. 2023; 03:58–69. doi: 10.13902/j.cnki.syyj.2023.03.015

[14] Liu Y, Li XN. Comparison and optimization path research of smart community elderly care model in China in digital era. E-Government. 2022; 233(05):112–124. doi: 10.16582/j.cnki.dzzw.2022.05.011

[15] Zhang JT, Rui GQ. Big data-driven smart and healthy elderly care: realistic representation, internal elements, and optimization paths. Social Sciences in Hunan. 2023; 05:132–141. http://kns.cnki.net/kcms/detail/43.1161.C.20231010.1551.034.html.

[16] Wu X. Development trend, realistic dilemma, and optimization path of the smart elderly care industry. East China Economic Management. 2021; 35(07):1–9. doi: 10.19629/j.cnki.34-1014/f.210311013

[17] gov.cn [Internet]. Smart healthy senior care industry to surpass 4 trillion RMB; c2020 [cited 2023 Jun 5]. Available from: https://www.gov.cn/xinwen/2020-01/04/content_5466410.htm.

[18] He W. The current situation and optimization of the supply structure of home care services. Journal of Hubei University (Philosophy and Social Science Edition). 2020; 47(06):155–165. doi: 10.13793/j.cnki.42-1020/c.2020.06.018

[19] Zhao HH. Rethinking of community mutual aid service for the aged from the perspective of cooperative production. Administrative Tribune. 2023; 30(05):149–155. doi: 10.16637/j.cnki.23-1360/d.2023.05.001

[20] Liu CJ, Li S, Guo JY. Comparative analysis of domestic and international research on smart elderly care: topic selection, theoretical differentiation and methodological orientation. E-Government. 2023; 09:90–104. doi: 10.16582/j.cnki.dzzw.2023.09.008

[21] Zhang B. The intelligent community nursing service mode in the view of "Internet +". Contemporary Economic Management. 2019; 41(06):45–50. doi: 10.13253/j.cnki.ddjjgl.2019.06.007

[22] Zhu WP, Lin Y. The construction of the community-based elderly care mode with "combination of medical care and elderly care" in Japan and its enlightenment to China-based on the perspective of institutional analysis. Southwest Finance. 2022; 01:76–87.

[23] Zhang L, Xu XY. Construction of smart older adults care service model driven by primary health care. Front. Public Health. 2023; 11:1157758. doi: 10.3389/fpubh.2023.1157758 37139396 PMC10150925

[24] McDonald T, Russell F. Long-term care quality-of-life scale utility in community home care. Nursing & Health Sciences. 2019; 21(4): 494–500. 10.1111/nhs.12628 31286647

[25] Powell J, Li X. Integrated, data-driven health management: A step closer to personalized and predictive healthcare. Cell Systems. 2022; 13(3): 201–203. 10.1016/j.cels.2022.02.001 35298911

[26] de Jong L, Plöthner M, Stahmeyer JT, Eberhard S, Zeidler J, Damm K. Informal and formal care preferences and expected willingness of providing elderly care in Germany: protocol for a mixed-methods study. BMJ OPEN. 2019; 9:e023253. doi: 10.1136/bmjopen-2018-023253 30647033 PMC6340479

[27] Melkas H, Hennala L, Pekkarinen S, Kyrki V. Impacts of robot implementation on care personnel and clients in elderly-care institutions. International Journal of Medical Informatics. 2020; 134:104041. 10.1016/j.ijmedinf.2019.104041 31838291

[28] Muangprathub J, Sriwichian A, Wanichsombat A, Kajornkasirat S, Nillaor P, Boonjing V. A novel elderly tracking system using machine learning to classify signals from mobile and wearable sensors. International Journal of Environmental Research and Public Health. 2021; 18(23):12652. 10.3390/ijerph182312652 34886377 PMC8656729

[29] Qian K, Zhang ZX, Yamamoto Y, Schuller. Artificial intelligence Internet of Things for the elderly: from assisted living to health-care monitoring. IEEE Signal Processing Magazine. 2021; 38(4):78–88. doi: 10.1109/MSP.2021.3057298

[30] Jaana M, Pare G. Comparison of mobile health technology use for self-tracking between older adults and the general adult population in Canada: cross-sectional survey. JMIR Mhealth and Uhealth. 2020; 8(1):e24718. https://preprints.jmir.org/preprint/24718. doi: 10.2196/24718 33104517 PMC7717921

[31] Zhang Z, Mao YH, Hu YC. A study on willingness to use intelligent elderly care services from the perspective of elderly digital divide. Northwest Population. 2023; 44(01):104–115. doi: 10.15884/j.cnki.issn.1007-0672.2023.01.008

[32] Kumar H, Singh MK, Gupta MP, Madaan J. Moving towards smart cities: Solutions that lead to the smart city transformation framework. Technological Forecasting & Social Change. 2020; 153:119281. 10.1016/j.techfore.2018.04.024.

[33] Neirotti P, De Marco A, Cagliano AC, Mangano G, Scorrano F. Current trends in Smart City initiatives: some stylised facts. Cities. 2014; 38:25–36. 10.1016/j.cities.2013.12.010.

[34] Lee J, Lee H. Developing and validating a citizen-centric typology for smart city services. Government Information Quarterly. 2014; 31(1):S93–S105. 10.1016/j.giq.2014.01.010.

[35] Wang F. Organization transformation of government in the context of smart society. Chinese Public Administration. 2019; (07):89–93. doi: 10.19735/j.issn.1006-0863.2019.07.12

[36] Schedler K, Guenduez AA, Frischknecht R. How smart can government be? Exploring barriers to the adoption of smart government. Information Polity. 2019; 24:3–20.

[37] Hujran O, Al-Debei MM, Al-Adwan AS, Alarabiat A, Altarawneh N. Examining the antecedents and outcomes of smart government usage: An integrated model. Government Information Quarterly. 2023; 40:101783. 10.1016/j.giq.2022.101783.

[38] Guo X, Li ZR. A government transformation driven by the new generation of information technology-from the network government to the data government and the smart government. Administrative Tribune. 2018; 25(04):56–60. doi: 10.16637/j.cnki.23-1360/d.2018.04.009

[39] Sun X, Yu H, Solvang WD. Towards the smart and sustainable transformation of reverse logistics 4.0: A conceptualization and research agenda. Environmental Science and Pollution Research. 2022; 29:69275–69293. 10.1007/s11356-022-22473-3 35972653 PMC9378263

[40] Liu H, Chen H, Zhang H, Liu H, Yu X, Zhang S. Contract design of logistics service supply chain based on smart transformation. Sustainability. 2022; 14(10):6261. 10.3390/su14106261.

[41] Senthil Kumar A, Suresh G, Lekashri S, Babu Loganathan G, and Manikandan R. Smart agriculture system with E–carbage using IoT. International Journal of Modern Agriculture. 2021; 10:928–931.

[42] Okano MT, Antunes SN, Fernandes ME. Digital transformation in the manufacturing industry under the optics of digital platforms and ecosystems. Independent Journal of Management & Production. 2021; 12:1139–1159. 10.14807/ijmp.v12i4.1375.

[43] Yin S, Wang Y and Xu J. Developing a conceptual partner matching framework for digital green innovation of agricultural high-end equipment manufacturing system toward agriculture 5.0: A novel niche field model combined with fuzzy VIKOR. Front. Psychol. 2022; 13:924109. doi: 10.3389/fpsyg.2022.924109 35874394 PMC9304958

[44] Yin S, Zhang N, Ullah K, Gao S. Enhancing digital innovation for the sustainable transformation of manufacturing industry: A pressure-state-response system framework to perceptions of digital green innovation and its performance for green and intelligent manufacturing. Systems. 2022; 10:72. 10.3390/systems10030072.

[45] Ren Y, Zhu Y, Guan BW. The role of government in community home care services. Study & Exploration. 2022; 09:16–26.

[46] Li LY, Li L, Deng YY. Theoretical logic, content attribute and experience inspiration of public policies of family support for the elderly in developed countries. Social Security Studies. 2020; 06:57–67.

[47] Herd P, Favreault M, Meyer MH, Smeeding TM. A targeted minimum benefit plan: A new proposal to reduce poverty among older social security recipients. RSF-The Russell Sage Journal of The Social Sciences. 2018; 4(2):74–90. doi: I10.7758/RSF.2018.4.2.04

[48] Zhou C, Han ZY, Qian ZJ. Government support and the development of private elderly care institutions under the perspective of active aging- An analysis based on dynamic evolutionary game. Contemporary Economic Management. 2022; 44(08):73–81. doi: 10.13253/j.cnki.ddjjgl.2022.08.010

[49] Liao CH, Chen J. Research on the influencing factors of user adoption intention of smart elderly care services under the background of comprehensive health industry: based on the perspective of perceived quality. Modern Management Science. 2021; 328(05):109–120.

[50] Liu HL. Research on smart community elderly care service under the hierarchy of needs theory: A case study of Taiyuan city. Legality Vision. 2019; (05):58–59.

[51] De Santis KK, Mergenthal L, Christianson L, Busskamp A, Vonstein C, Zeeb H. Digital technologies for health promotion and disease prevention in older people: Scoping review. Journal of Medical Internet Research. 2023; 25:e43542. doi: 10.2196/43542 36951896 PMC10131689

[52] Xu XY. Resource shortage or resource dependence: resource dilemma in intelligent community old-age. Lanzhou Academic Journal. 2019; 308(05):196–208.

[53] Yu YY, Yin S. Incentive mechanism for the development of rural new energy industry: New energy enterprise-village collective linkages considering the quantum entanglement and benefit relationship. International Journal of Energy Research. 2023; 2023:1675858. doi: 10.1155/2023/1675858

[54] Mao QH, Xu LY, Wu RW. Evolutionary game of stakeholders’ behavioral strategies in wetland ecosystems from the vulnerability perspective. Environmental Science and Pollution Research. 2023; 30:43419–43439. 10.1007/s11356-023-25300-5 36658314

[55] Yin S, Li B. A stochastic differential game of low carbon technology sharing in collaborative innovation system of superior enterprises and inferior enterprises under uncertain environment. Open Mathematics. 2018; 16(1): 607–622. 10.1515/math-2018-0056.

[56] Smith J, Price GR. The logic of animal conflict. Nature. 1973; 246: 15–18.

[57] Simon HA. Theories of decision-making in economics and behavioral science. American Economic Review. 1959; 49:253–283.

[58] Adami C, Schossau J, Hintze A. Evolutionary game theory using agent-based methods. Physics of Life Reviews. 2016; 19:1–26. doi: 10.1016/j.plrev.2016.08.015 27617905

[59] Yang ZJ, Liu B, Bi KX. Evolutionary game study of green innovation diffusion in domestic and foreign firms under government control. Soft Science. 2019; 33(12):86–91. doi: 10.13956/j.ss.1001-8409.2019.12.14

[60] Li XD, Yuan Y, Huang LC. Evolutionary game research on the resilience development of the emerging gerontechnology industry. Journal of Harbin Engineering University. 2023; 44(02):300–306.

[61] Tang J, He T. From "fragmented supply" to "collaborative governance": Logical reconstruction of the good governance of the main suppliers of the community-based medical-care integration in the perspective of the stakeholder theory. Journal of Yunnan Minzu University. 2022; 39(05):52–59. doi: 10.13727/j.cnki.53-1191/c.20220905.015

[62] Shao QH, Yuan JF, Ma JW, Ding HX, Huang W. Exploring the determinants of synergetic development of social organizations participating in home-based elderly care service: An SEM method. PLoS ONE. 2021; 15(12):e0244880. doi: 10.1371/journal.pone.0244880 33382827 PMC7775099

[63] Tang J, Peng G. The path reconstruction of governance for socialized elderly care in rural areas-An analysis based on stakeholder theory. Rural Economy. 2019; 08:136–142.

[64] Wu L, Xu JL. Study on the realization mechanism of social organizations’ responsibility in the government’s purchase of public services-A stakeholder theory perspective. Theory Monthly. 2017; 09:130–136. doi: 10.14180/j.cnki.1004-0544.2017.09.023

[65] Cui X, Cheng M, Dun S. A game analysis of service supply of residential care facilities under the background of market-oriented reform. Operations Research and Management Science. 2022; 31(10):61–67. doi: 10.12005/orms.2022.0320

[66] He Q, Hu B, Wang RY. Platform dynamic incentive, consumer adoption and digital content innovation- A three-party evolutionary game analysis. Operations Research and Management Science. 2022; 31(09):41–48. https://kns.cnki.net/kcms/detail/34.1133.G3.20211209.0326.002.html.

[67] Ritzberger K, Weibull JW. Evolutionary selection in normal-form games. Econometrica. 1995; (6). doi: 10.2307/2171774

[68] Zolfagharian M, Romme A, Walrave B. Why, when, and how to combine system dynamics with other methods: towards an evidence-based framework. Journal of Simulation. 2018; 12(2):98–114. 10.1080/17477778.2017.1418639.

[69] luan.gov.cn [Internet]. Lu’an City 2022 Implementation Plan for Elderly Services and Smart Elderly Care; c2022 [cited 2016 May 30]. Available from: http://jkq.luan.gov.cn/msgc/zcjd/24601531.html.

[70] Yue XH, Lin YM. The effectiveness of the feedback from elderly users in elderly care services: an evolutionary game. Journal of Jiangxi University of Finance and Economics. 2022; 142(04):71–82. doi: 10.13676/j.cnki.cn36-1224/f.2022.04.001

[71] Tian TT, Sun SH. Low-carbon transition pathways in the context of carbon-neutral: A quadrilateral evolutionary game analysis. Journal of Environmental Management. 2022; 322:116105. doi: 10.1016/j.jenvman.2022.116105

[72] Long YQ. Logic mechanism and practice path of collaborative governance of smart home-based elderly care services. Administration Reform. 2023; (07):50–58. doi: 10.14150/j.cnki.1674-7453.2023.07.005

[73] Li CY. Theoretical analysis and practical exploration of improving the quality and efficiency of financial policies for elderly care services: from the perspective of supply and demand. Qinghai Social Sciences. 2022; (03):107–116. doi: 10.14154/j.cnki.qss.2022.03.018

[74] Hu X. Analysis of fiscal and tax policies supporting the development of old-age service industry in China. Social Sciences in Hunan. 2017; 182(4):143–148.

[75] Jiang QP, Liu YY, Xu BH. Industrial digital transformation and upgrading of residents’ consumption structure- effects, pathways and mechanisms. Review of Industrial Economics. 2023; (04):67–89. doi: 10.19313/j.cnki.cn10-1223/f.20230606.001

[76] Xu KL, Qian Q, Xu GJ. Equalization of basic public services and expansion and upgrade of consumption: An analysis based on panel model and panel quantile regression model. Inquiry into Economic Issues. 2020; (06):28–42.

