# Supplementary figures and images for: Smart transition pathways and development incentive mechanism of China’s smart community elderly care industry under market dominance: Considering a multi-subjective behavior game
Source: PLoS One. 2024 May 31;19(5):e0297696. doi: 10.1371/journal.pone.0297696 (PMC11142596; doi:10.1371/journal.pone.0297696)

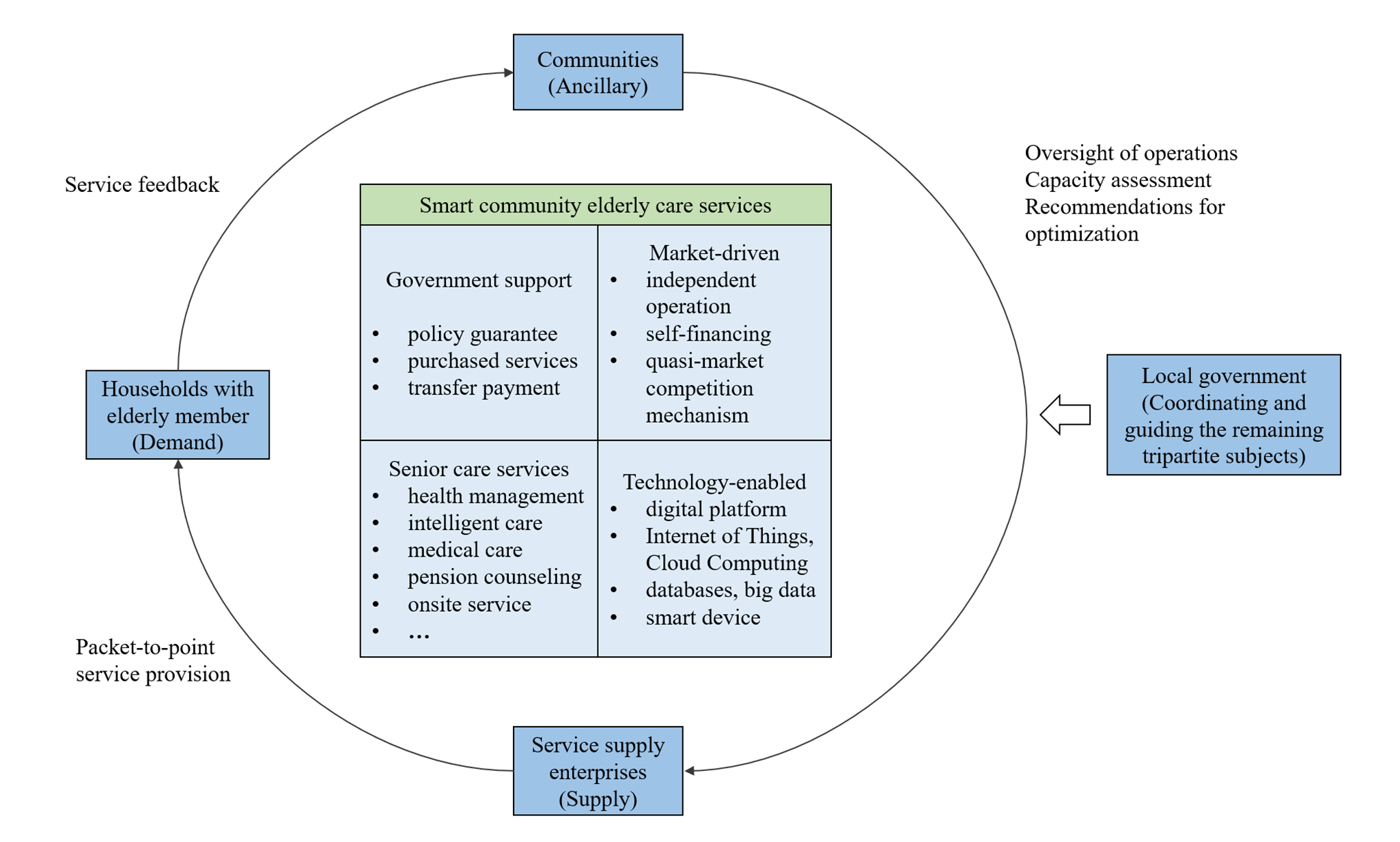

Supplement: S1 Fig — (ZIP) [file pone.0297696.s002.zip › S1_Figs/Fig 1.tif]

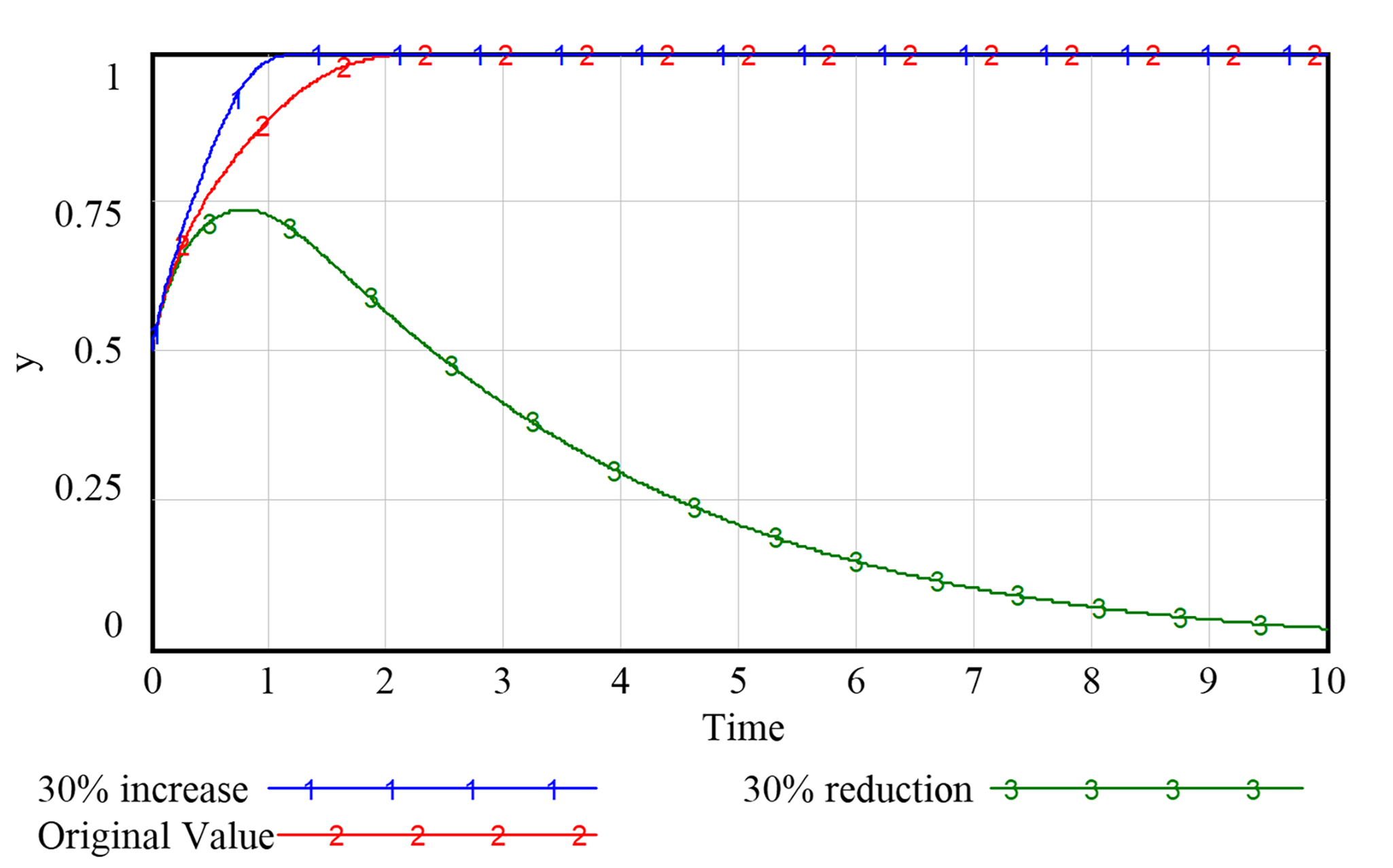

Supplement: S1 Fig — (ZIP) [file pone.0297696.s002.zip › S1_Figs/Fig 10.tif]

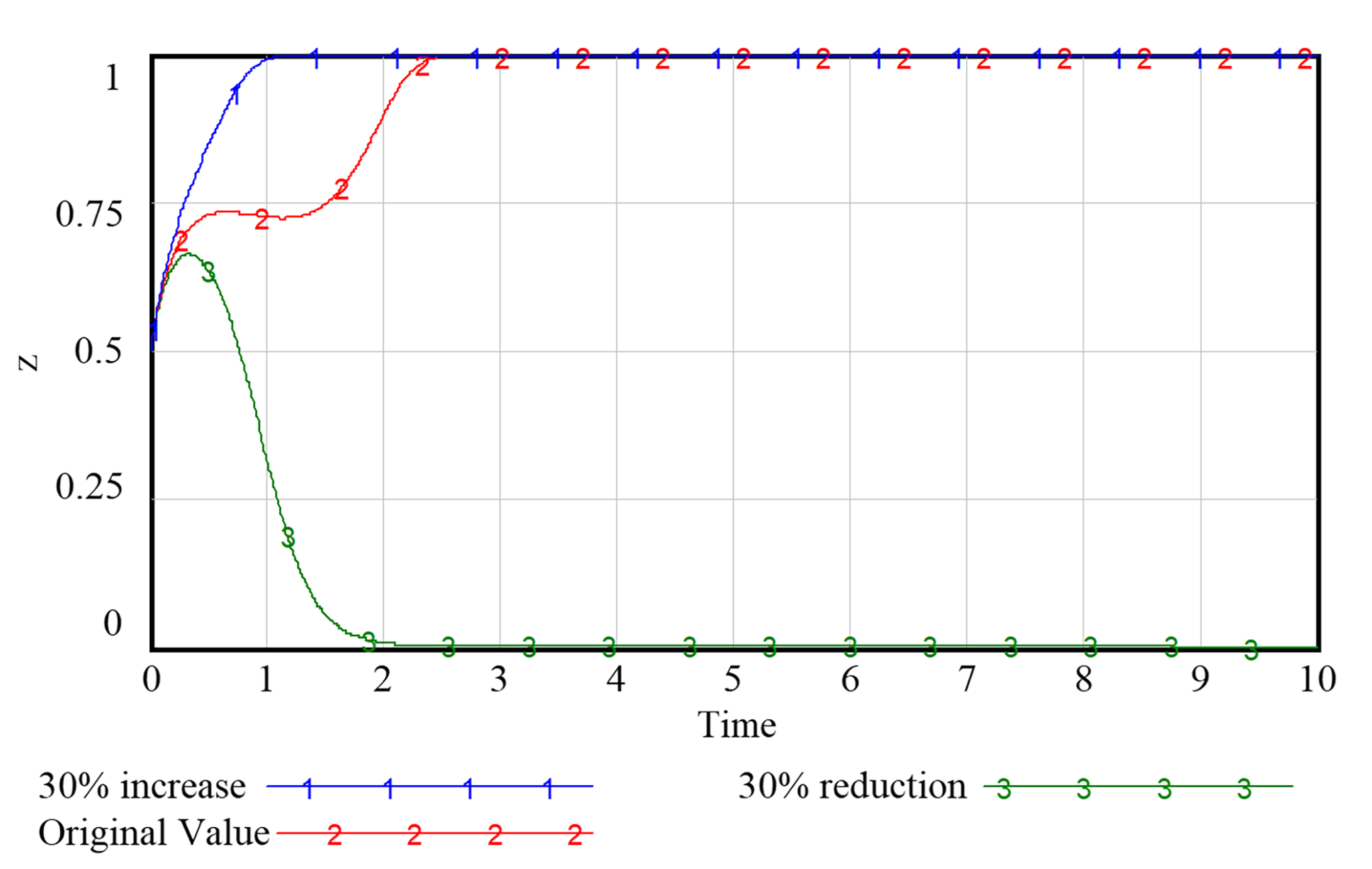

Supplement: S1 Fig — (ZIP) [file pone.0297696.s002.zip › S1_Figs/Fig 11.tif]

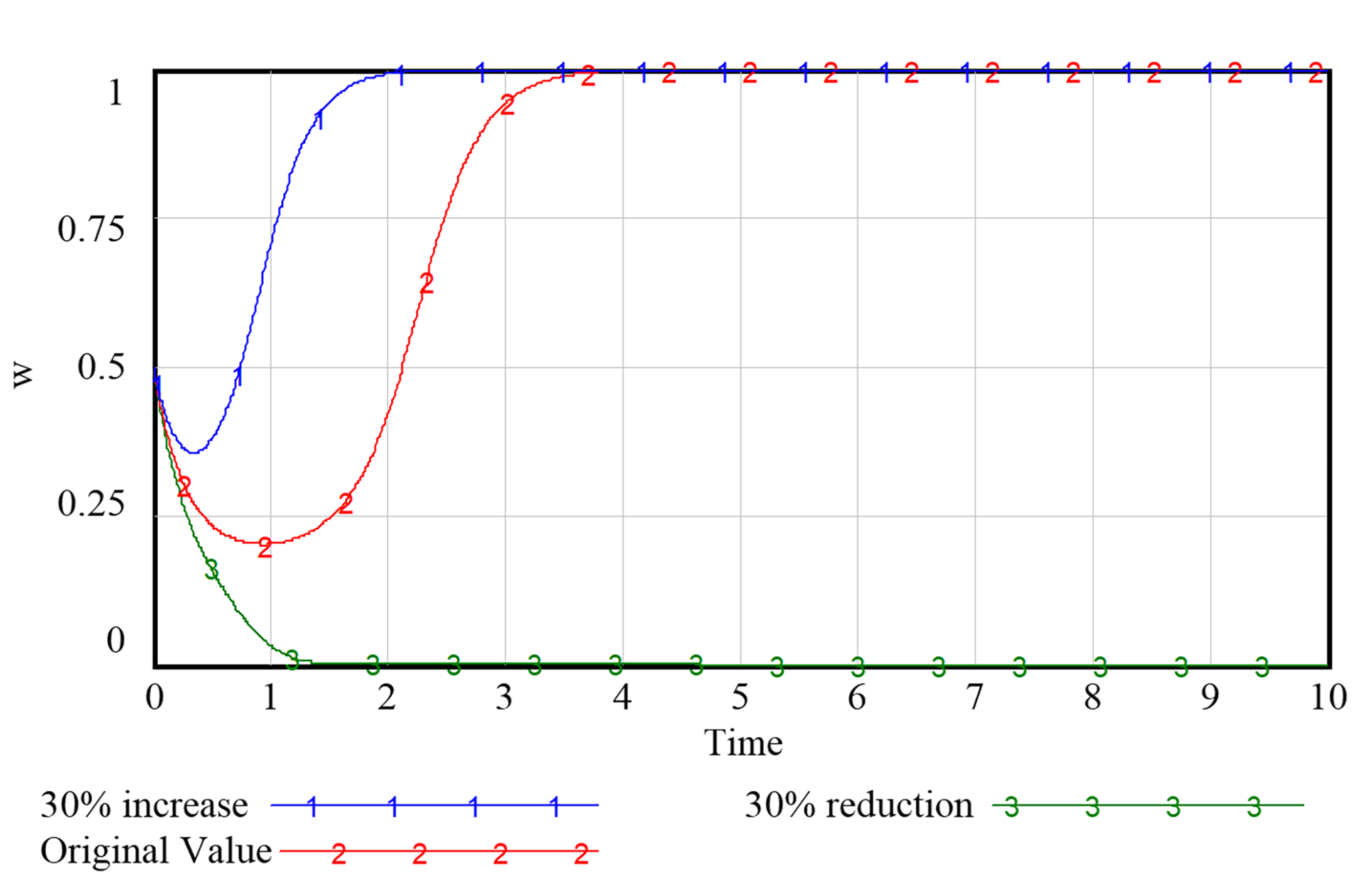

Supplement: S1 Fig — (ZIP) [file pone.0297696.s002.zip › S1_Figs/Fig 12.tif]

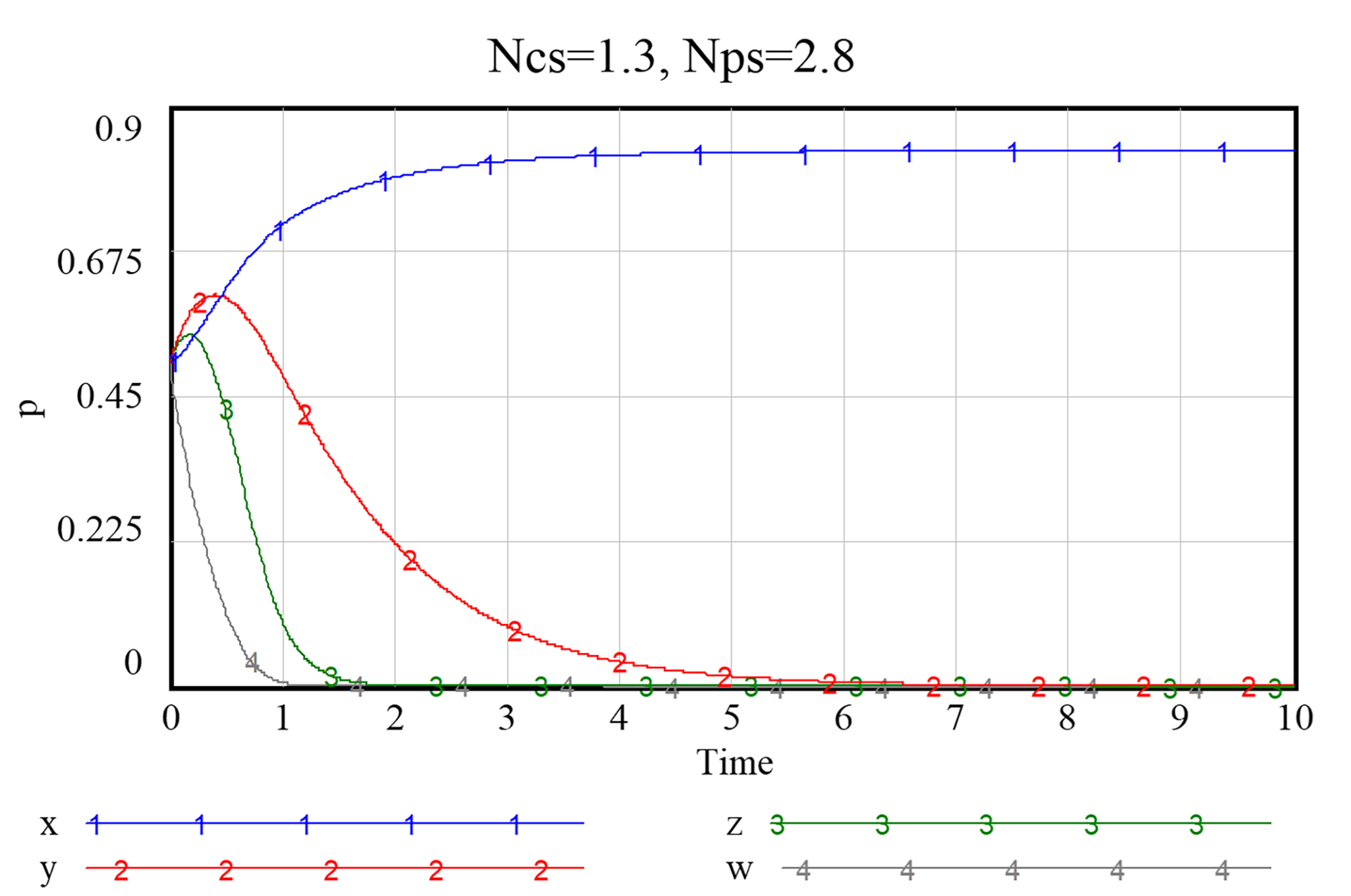

Supplement: S1 Fig — (ZIP) [file pone.0297696.s002.zip › S1_Figs/Fig 13.tif]

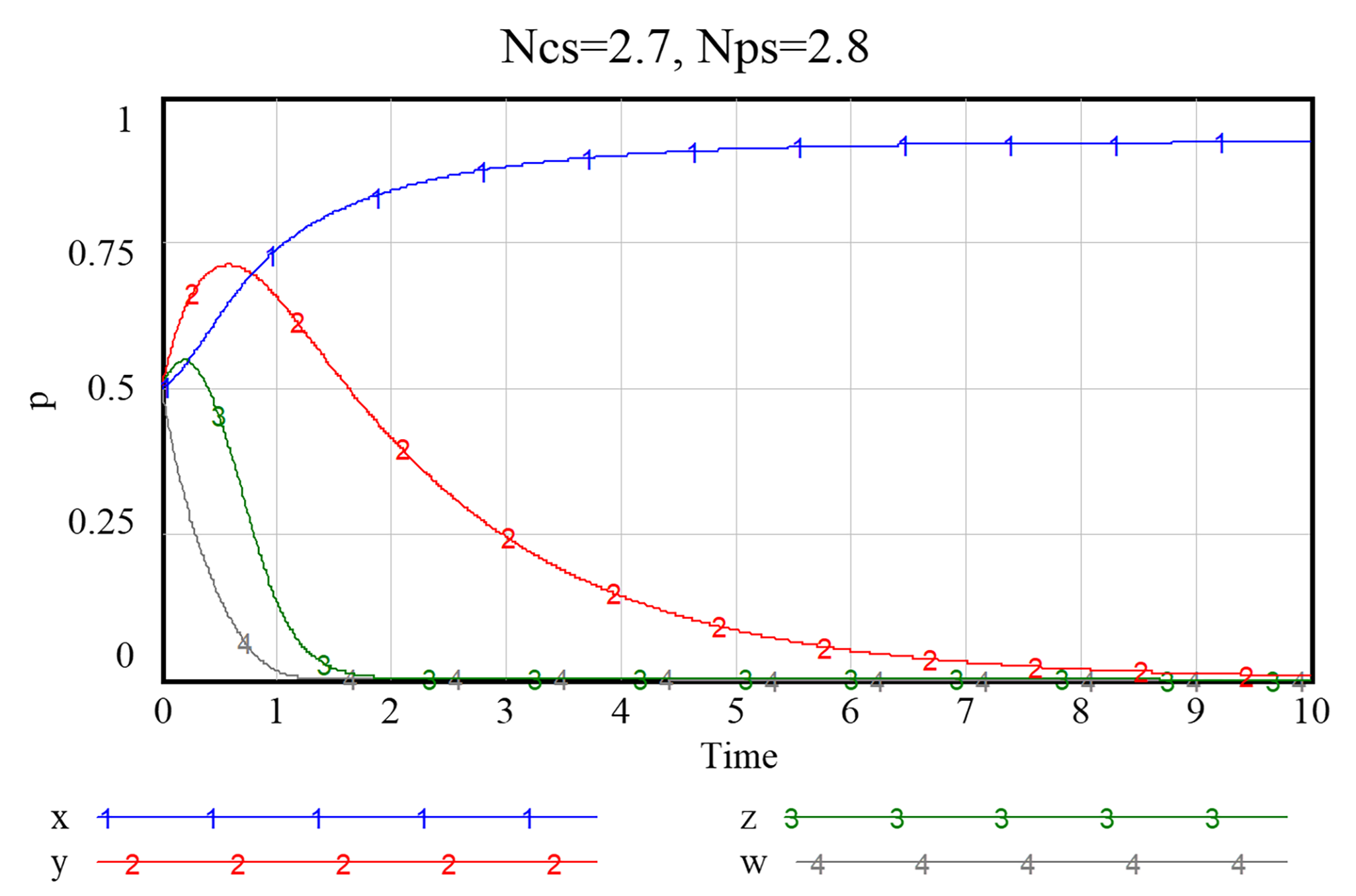

Supplement: S1 Fig — (ZIP) [file pone.0297696.s002.zip › S1_Figs/Fig 14.tif]

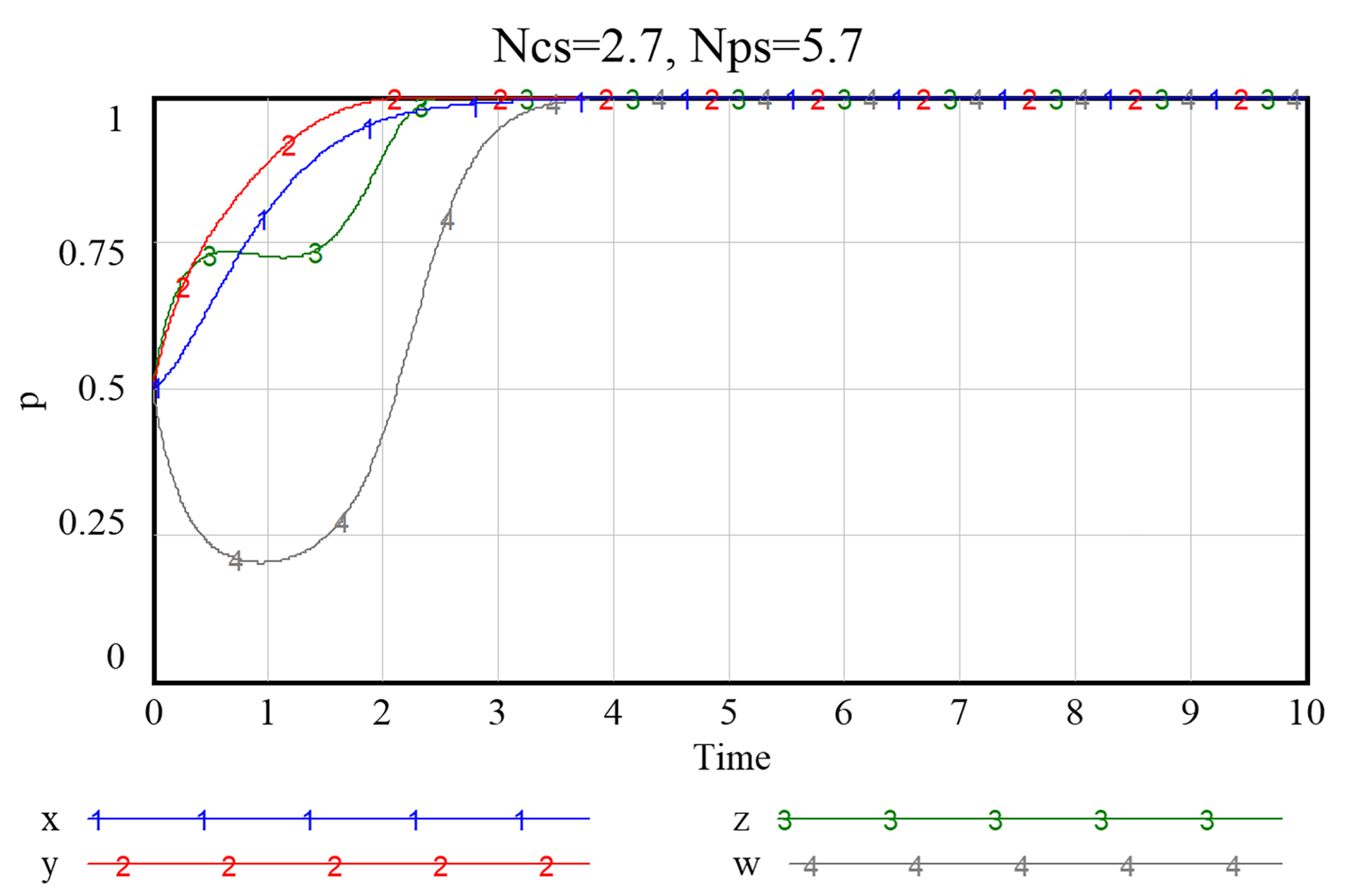

Supplement: S1 Fig — (ZIP) [file pone.0297696.s002.zip › S1_Figs/Fig 15.tif]

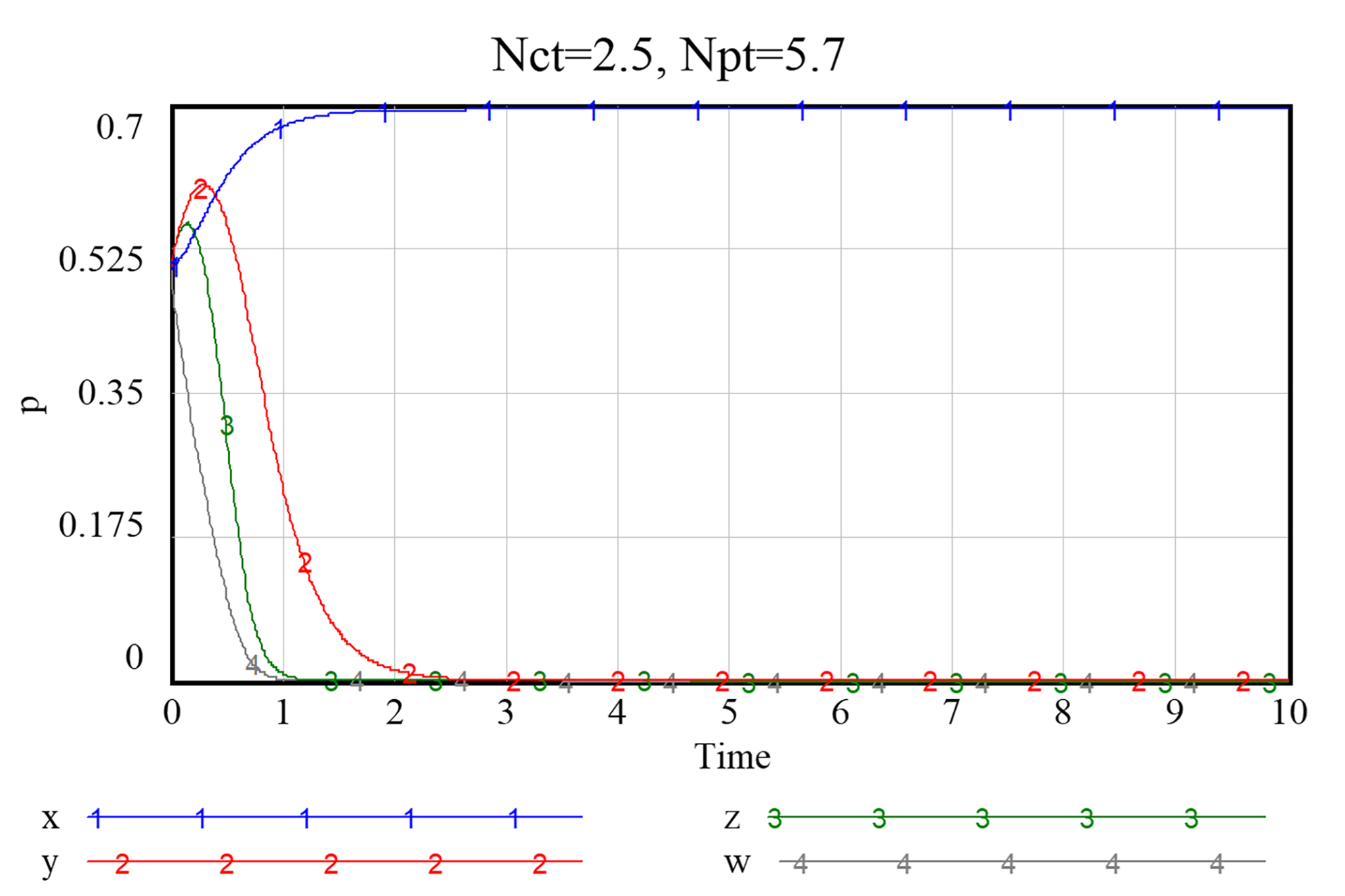

Supplement: S1 Fig — (ZIP) [file pone.0297696.s002.zip › S1_Figs/Fig 16.tif]

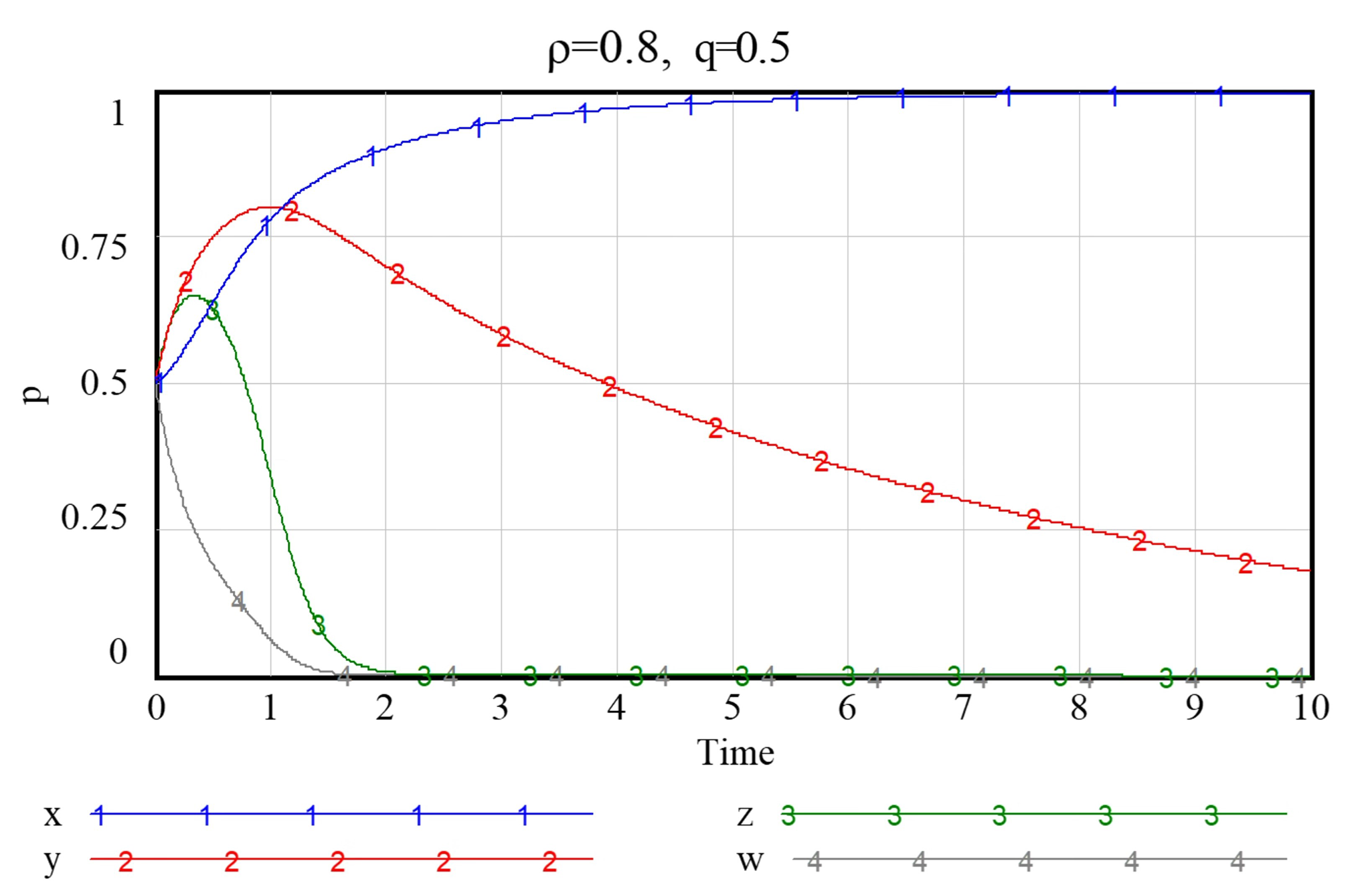

Supplement: S1 Fig — (ZIP) [file pone.0297696.s002.zip › S1_Figs/Fig 17.tif]

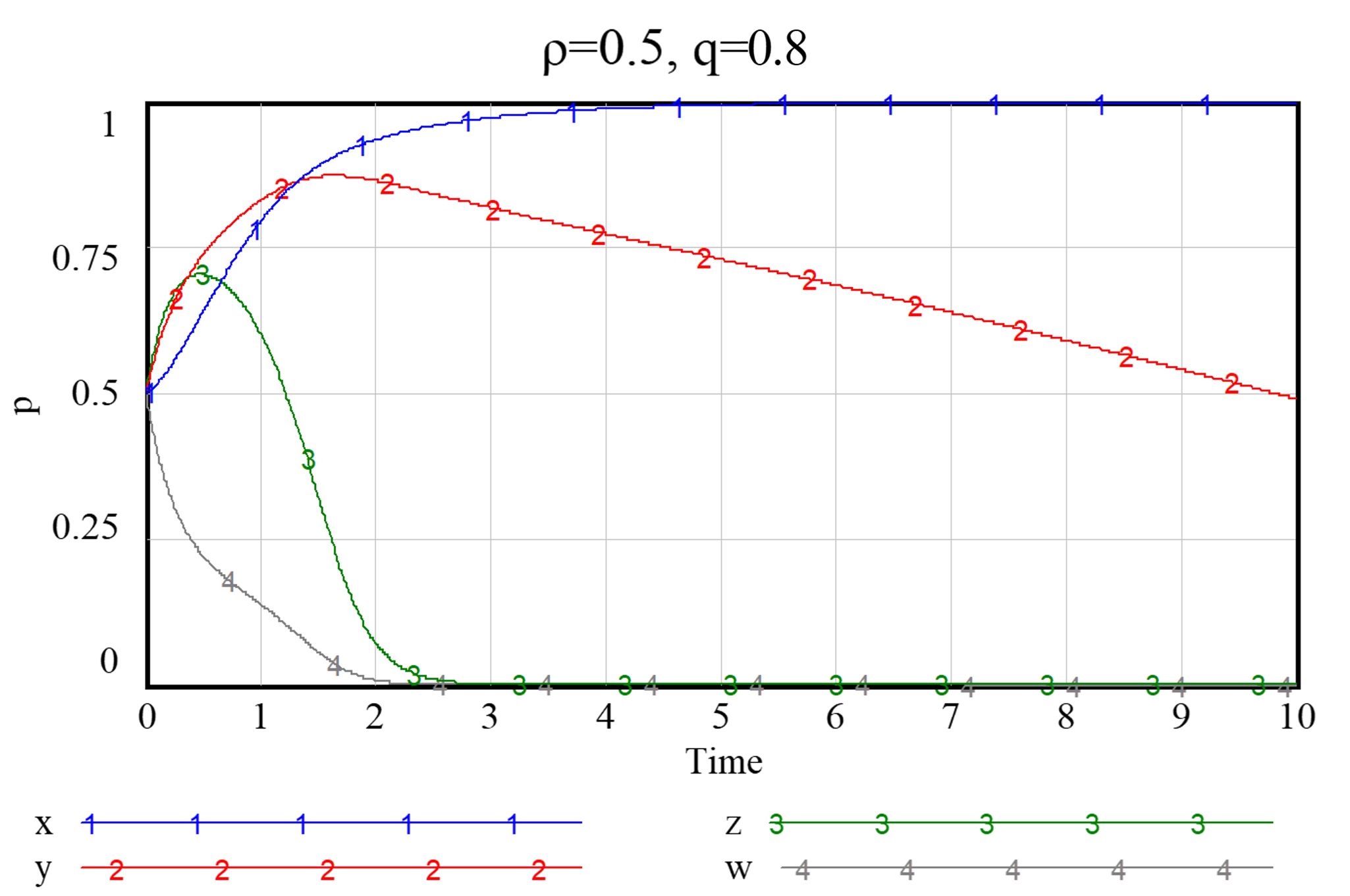

Supplement: S1 Fig — (ZIP) [file pone.0297696.s002.zip › S1_Figs/Fig 18.tif]

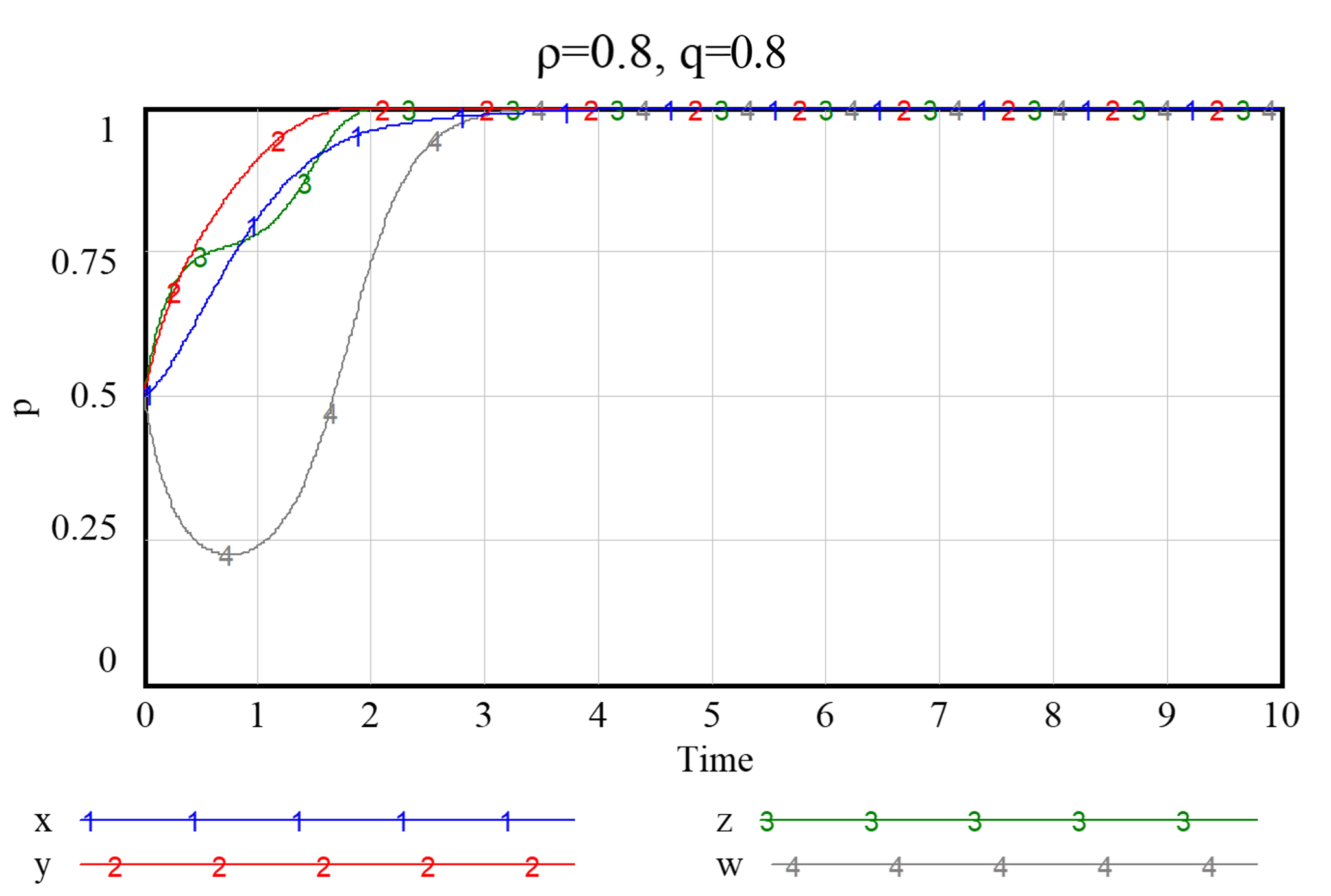

Supplement: S1 Fig — (ZIP) [file pone.0297696.s002.zip › S1_Figs/Fig 19.tif]

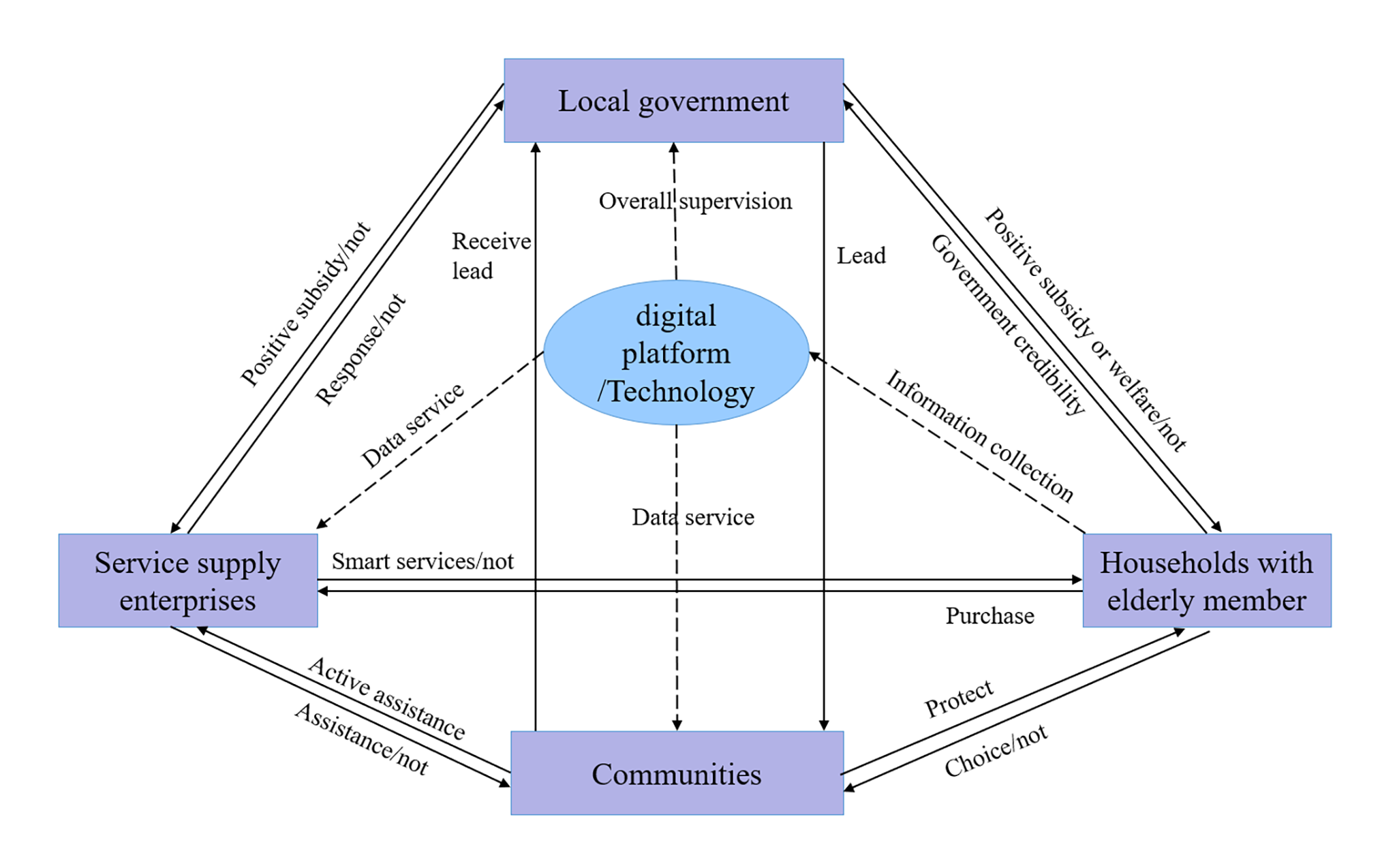

Supplement: S1 Fig — (ZIP) [file pone.0297696.s002.zip › S1_Figs/Fig 2.tif]

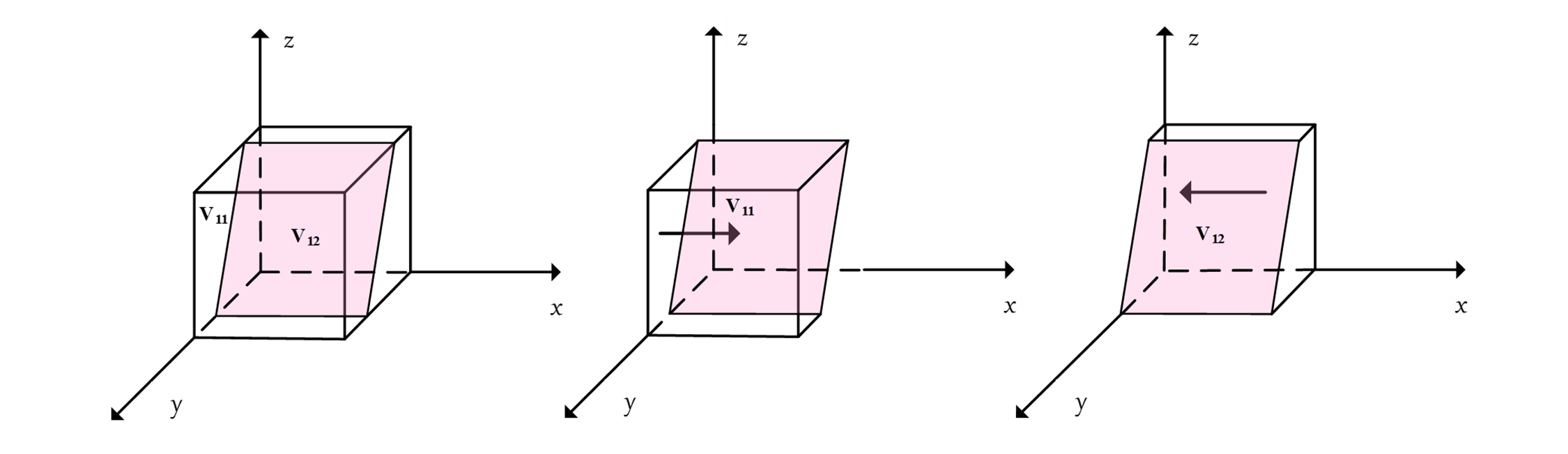

Supplement: S1 Fig — (ZIP) [file pone.0297696.s002.zip › S1_Figs/Fig 3.tif]

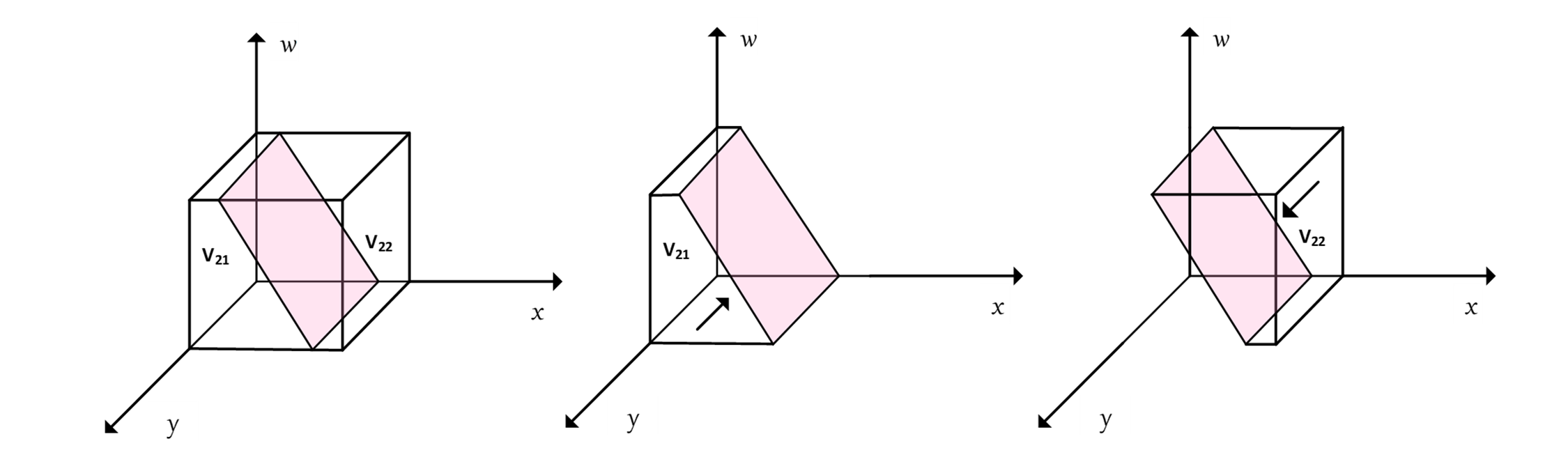

Supplement: S1 Fig — (ZIP) [file pone.0297696.s002.zip › S1_Figs/Fig 4.tif]

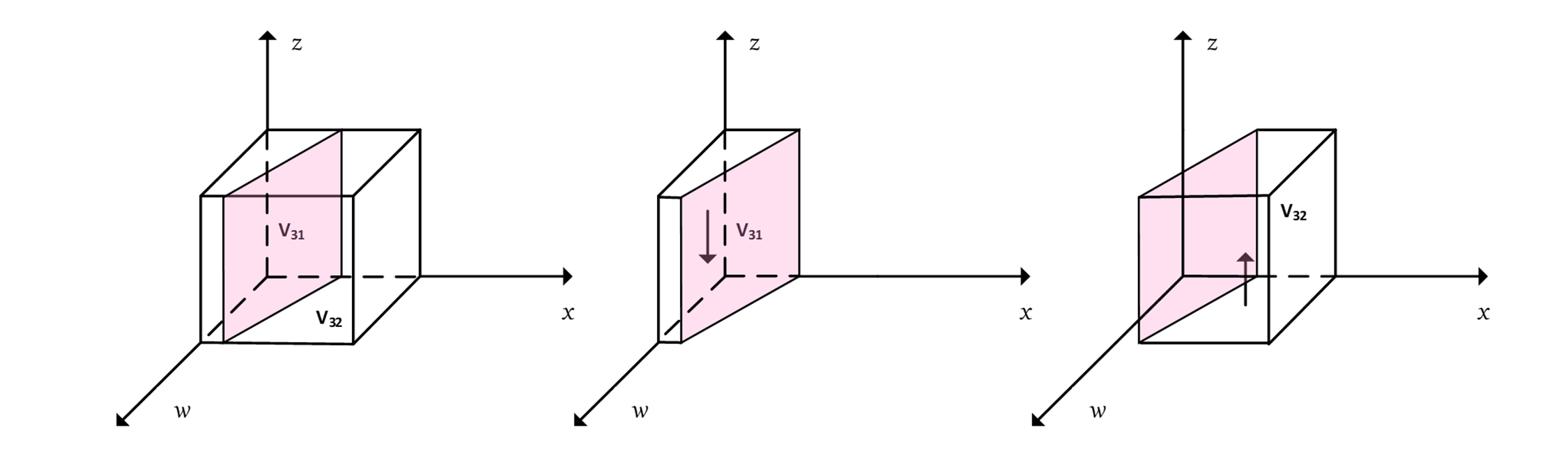

Supplement: S1 Fig — (ZIP) [file pone.0297696.s002.zip › S1_Figs/Fig 5.tif]

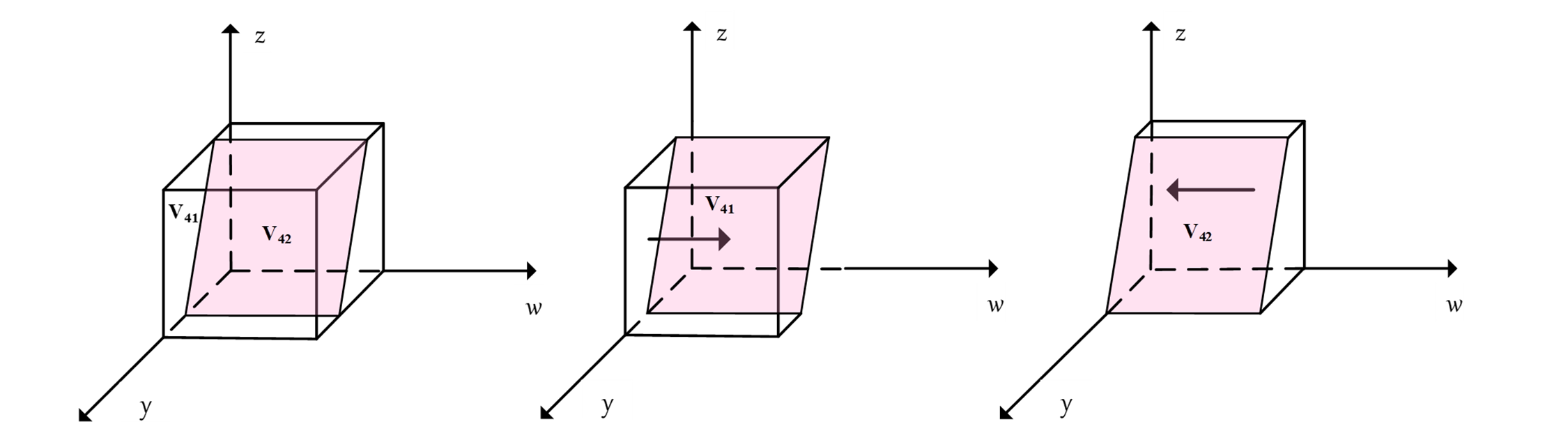

Supplement: S1 Fig — (ZIP) [file pone.0297696.s002.zip › S1_Figs/Fig 6.tif]

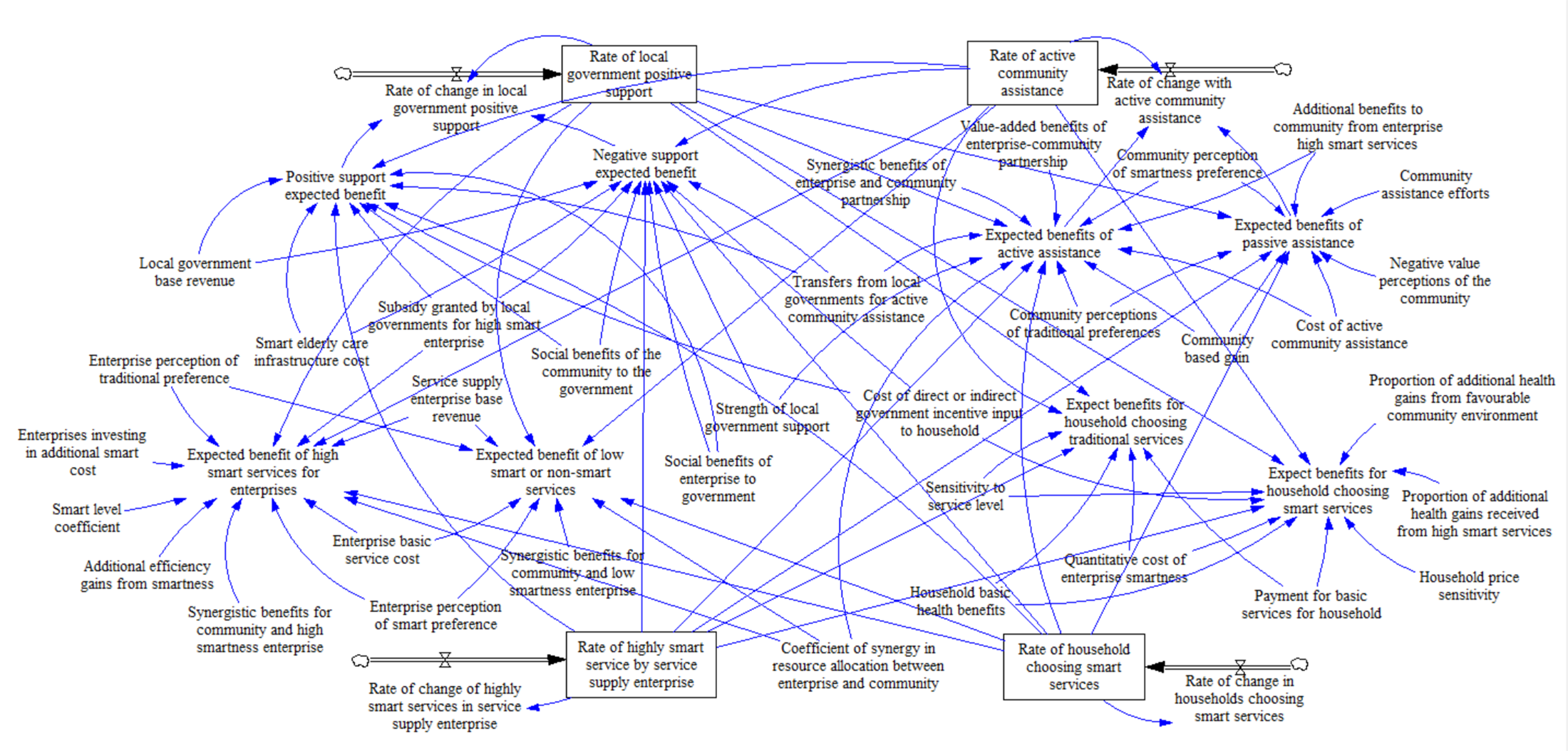

Supplement: S1 Fig — (ZIP) [file pone.0297696.s002.zip › S1_Figs/Fig 7.tif]

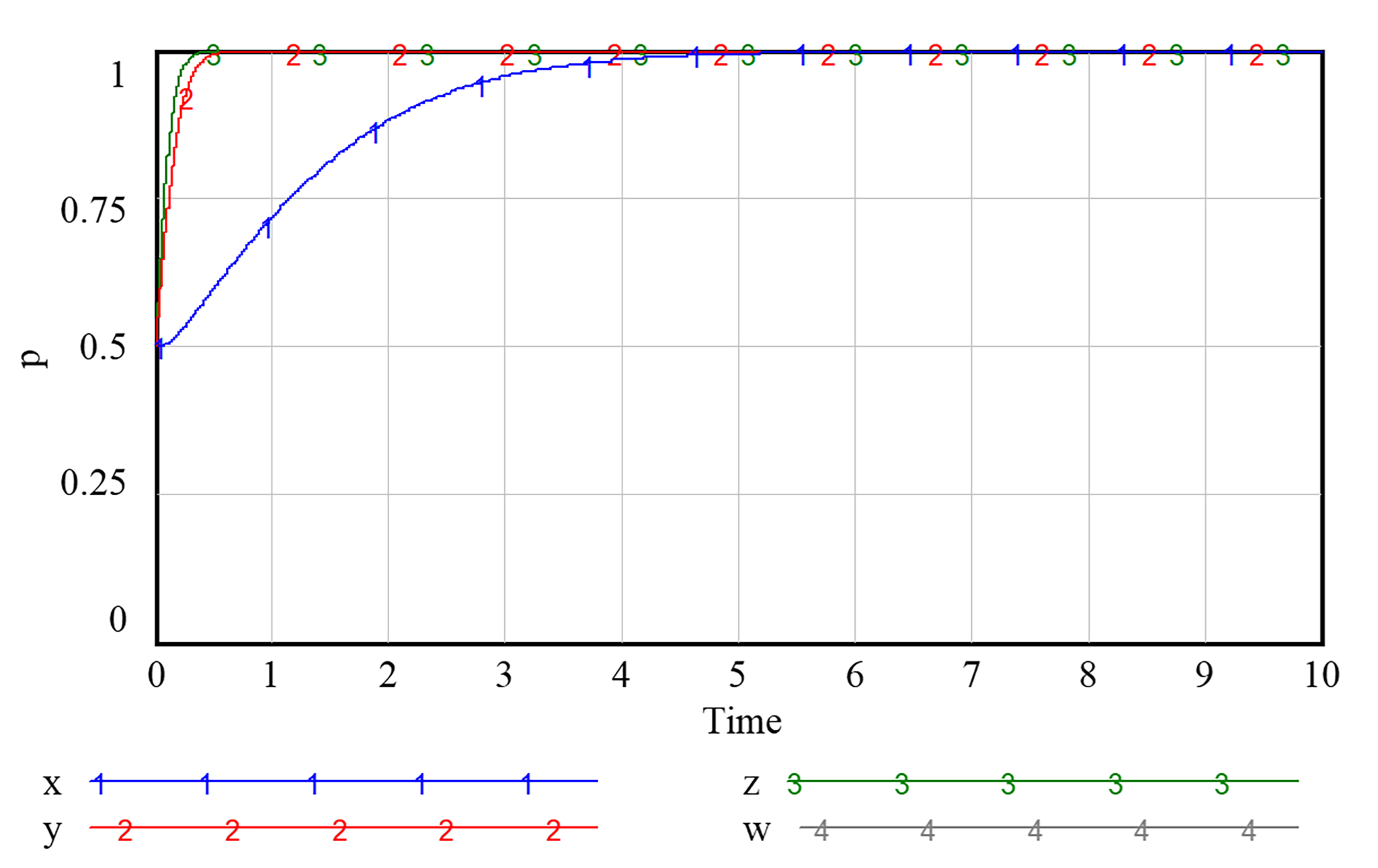

Supplement: S1 Fig — (ZIP) [file pone.0297696.s002.zip › S1_Figs/Fig 8.tif]

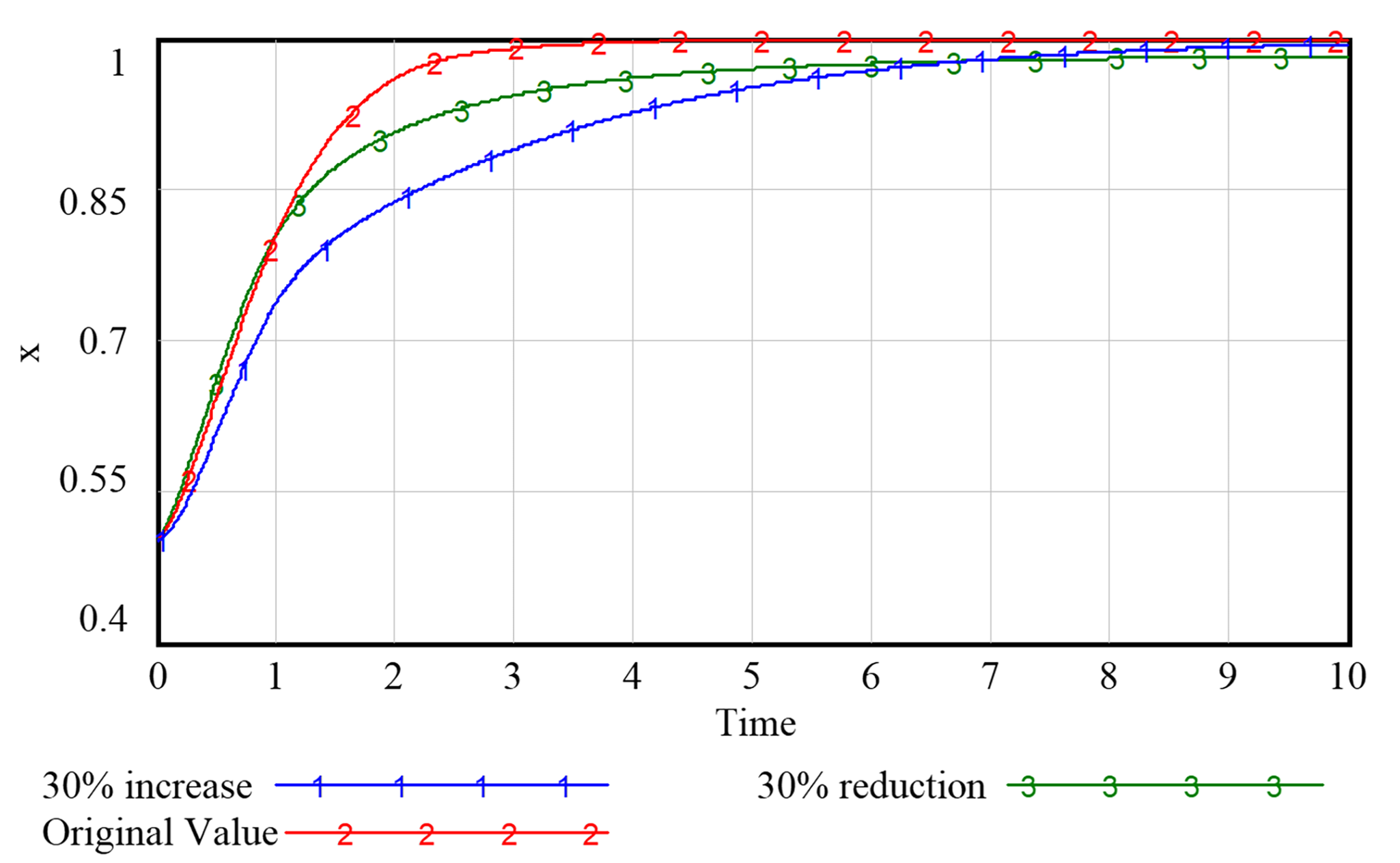

Supplement: S1 Fig — (ZIP) [file pone.0297696.s002.zip › S1_Figs/Fig 9.tif]
